# Magnetic Resonance Relaxation Anisotropy: Physical Principles and Uses in Microstructure Imaging

**DOI:** 10.1016/j.bpj.2017.02.026

**Published:** 2017-04-11

**Authors:** Michael J. Knight, Serena Dillon, Lina Jarutyte, Risto A. Kauppinen

**Affiliations:** 1 School of Experimental Psychology, University of Bristol, Bristol, United Kingdom; 2 ReMemBr group, Institute for Clinical Neurosciences, University of Bristol, Bristol, United Kingdom; 3 Clinical Research and Imaging Centre, University of Bristol, Bristol, United Kingdom

## Abstract

Magnetic resonance imaging (MRI) provides an excellent means of studying tissue microstructure noninvasively since the microscopic tissue environment is imprinted on the MRI signal even at macroscopic voxel level. Mesoscopic variations in magnetic field, created by microstructure, influence the transverse relaxation time (*T*_*2*_) in an orientation-dependent fashion (*T*_*2*_ is anisotropic). However, predicting the effects of microstructure upon MRI observables is challenging and requires theoretical insight. We provide a formalism for calculating the effects upon *T*_*2*_ of tissue microstructure, using a model of cylindrical magnetic field perturbers. In a cohort of clinically healthy adults, we show that the angular information in spin-echo *T*_*2*_ is consistent with this model. We show that *T*_*2*_ in brain white matter of nondemented volunteers follows a U-shaped trajectory with age, passing its minimum at an age of ∼30 but that this depends on the particular white matter tract. The anisotropy of *T*_*2*_ also interacts with age and declines with increasing age. Late-myelinating white matter is more susceptible to age-related change than early-myelinating white matter, consistent with the retrogenesis hypothesis. *T*_*2*_ mapping may therefore be incorporated into microstructural imaging.

## Introduction

The power of magnetic resonance imaging (MRI) is in its capability to deliver information on microstructure, meaning that one may manipulate the signal and imprint the signature of microscopic structures upon it. By such means, the existence and nature of objects very much smaller than a voxel may be inferred. This is nonetheless an evolving technology and advances in hardware, software, and theoretical understanding continue to extend its utility.

Understanding the microstructural changes taking place in the human brain with age is of great importance in identifying and treating diseases associated with aging, such as various classes of dementia and stroke. The dementia challenge is a particularly large one because we are currently limited to making diagnoses only once clinical presentation is severe. However, the identification of pathology before potentially irreversible loss of tissue must identify the chemical or microstructural causes in treatable tissue. Here, the availability of methods sensitive to widespread but subtle changes is particularly important. Our objective in this article is to develop and apply the phenomenon of transverse relaxation time (*T*_*2*_) anisotropy to reveal details of human white matter (WM).

Microstructure influences the range of resonance frequencies that a diffusing nuclear spin may sample, therefore influencing the coherence and decoherence of spin phase (thus signal amplitude) as well as total accumulated signal phase. The signature of microstructure is thereby imprinted on both MRI signal amplitude and phase. Several modalities exploit these two distinct phenomena, and in different ways. In diffusion imaging, external magnetic field gradients are applied. Coherence is lost more rapidly if an applied field gradient is parallel to a direction in which there is less restriction to translational diffusion on a micrometer scale such that a broad range of resonance frequencies is sampled (1). Therefore, one may infer the existence and nature of structures of micrometer size (2). In the absence of applied field gradients, microstructure nevertheless creates an inhomogeneous local magnetic field on a mesoscopic scale (3, 4, 5, 6, 7), due to differences in magnetic susceptibility within the microstructural components in the system. This influences the signal in gradient-echo and spin-echo imaging, as spins in a voxel sample a range of resonance frequencies—similarly to the application of a field gradient, but with the inhomogeneity arising from the system under study, rather than externally applied. In gradient-echo MRI, the signal accumulates phase, which has given rise to quantitative susceptibility mapping (QSM) (8, 9) and susceptibility tensor imaging (STI) (10). In spin-echo MRI, translational diffusion through the inhomogeneous field labels the *T*_*2*_ with the signature of the microstruturally induced local magnetic field (11). We have recently demonstrated this to be so in human WM, in which the spin-echo *T*_*2*_ of human WM shows a pattern of anisotropy by which its maximum occurs when an ordered system is parallel to the applied field *B*_0_ and minimized when perpendicular (12). We have also provided a theoretical framework by which it may be explained (13)—opening the door to applications of *T*_*2*_ anisotropy. However, relating microstructure to the perturbations to magnetic fields resulting from it, and thus to measurements of coherence lifetimes and diffusion-mediated dephasing, remains challenging. The link between microstructure and its influence on most MRI-observable quantities remains a challenging one to make especially in systems such as the brain.

In this article, we develop the principles of spin-echo *T*_*2*_ anisotropy and apply it to reveal details of human WM aging. By doing so, we reveal that, in a cohort of healthy persons, *T*_*2*_ anisotropy is sensitive not just to the particular WM tract, but the regional age effects. In particular, late-myelinating WM has markedly lower anisotropy and loses its anisotropy more rapidly in later life than early-myelinating WM. These findings are consistent with the retrogenesis theory (14). As such, we seek to establish relaxation anisotropy as a tool in the arsenal of microstructural imaging modalities.

### Theory section: the *b*-tensor field and coherence lifetime anisotropy

In a spin-echo experiment, where phase terms are entirely refocused, if there exists a resonance frequency inhomogeneity $\omega_{I}\left(x\right)$, due to mesoscopic susceptibility differences, applied field gradients or other sources, the signal amplitude evolves according to (13)

(1) $$S\left(t\right)=\left|\int A_{0}\left(x\right)\text{exp}\left(-b\left(x\right)\cdot D\left(x\right)\right)\text{exp}\left(-R_{2}^{iso}\left(x\right)t\right)dx\right|,$$

where *A*_0_ is the signal amplitude at (time) *t* = 0, D(x) is the translational diffusion tensor field, $R_{2}^{iso}\left(x\right)$ is the (isotropic) transverse relaxation rate coefficient scalar field, and we have introduced **b**(**x**) as the *b*-tensor field. The elements of the *b*-tensor field are defined as

(2) $$b_{jk}\left(x\right)=\frac{\rho^{2}t^{3}}{3}\frac{\partial \omega_{I}\left(x\right)}{\partial x_{j}}\frac{\partial \omega_{I}\left(x\right)}{\partial x_{k}},$$

where *ρ* is the coherence order (15). This is analogous to the theory common in diffusion imaging and replaces the *b*-value, to which it reduces if the frequency inhomogeneity $\omega_{I}\left(x\right)$ is linear (such as due solely to applied field gradients). We have also defined, in admittedly flexible notation:

(3) $$b\left(x\right)\cdot D\left(x\right)=\sum_{jk}b_{jk}\left(x\right)D_{jk}\left(x\right).$$

The fact that we have a *b*-tensor field implies that, provided $\omega_{I}\left(x\right)$ exists, diffusion-mediated decoherence, and therefore *T*_*2*_, is anisotropic. That is, the magnetic field created by tissue microstructure in response to the applied field transforms with orientation relative to the applied field, and the form of the spin phase decoherence transforms with it.

The frequency inhomogeneity function, $\omega_{I}\left(x\right)$, may be decomposed into a sum of terms representing the response of the system under observation to the applied field, and any deliberate inhomogeneity due to the use of applied field gradients:

(4) $$\begin{array}{c} \omega_{I}\left(x\right)=\Delta \omega \left(x\right)+\gamma G\cdot x\\ =\Delta \omega \left(x\right)+\omega_{D}\left(x\right) \end{array},$$

where Δω(x) is the frequency difference from the Larmor frequency arising due to magnetic susceptibility differences within the system, **G** is a field gradient, and $\omega_{D}\left(x\right)$ is the linear frequency shift arising due to applied (typically pulsed) field gradients. There is the following interaction between the effects of the frequency difference function and applied field gradients:

(5) $$b\cdot D=\frac{\rho^{2}t^{3}}{3}\sum_{j,k}\frac{\partial \left(\Delta \omega +\omega_{D}\right)}{\partial x_{j}}\frac{\partial \left(\Delta \omega +\omega_{D}\right)}{\partial x_{k}}D_{jk}=\frac{\rho^{2}t^{3}}{3}\left[\begin{array}{c} {\left[\nabla \Delta \omega \right]}^{T}\cdot D\nabla \Delta \omega \\ +{\left[\nabla \omega_{D}\right]}^{T}\cdot D\nabla \omega_{D}\\ +\left({\left[\nabla \Delta \omega \right]}^{T}\cdot D\nabla \omega_{D}+{\left[\nabla \omega_{D}\right]}^{T}\cdot D\nabla \Delta \omega \right) \end{array}\right].$$

On the second line of this equation, from top to bottom, the three terms represent dephasing due to the system’s response to the applied field only, dephasing due to the applied field gradients only, and dephasing due to the interaction between those effects. The superscript *T* represents the transpose operation. Additional details on this expansion are provided in the Supporting Material.

### Walled cylinder model

For the exploration of the effects of susceptibility differences, a model is useful. With a view to understanding the effects of myelinated axons upon diffusion-mediated decoherence in MRI of the human brain, we use a model of cylindrical field perturbers whose walls contain a material with a different magnetic susceptibility from their surroundings. The system-induced frequency difference Δω(x) may be calculated for any geometry of a set of cylindrical field perturbers (5) as

(6) $$\Delta \omega \left(x\right)=\sum_{m}\{\begin{array}{ll} \frac{\omega_{0}\chi_{m}}{2}{\text{sin}}^{2}\;\theta_{m}\text{cos}\;2\phi \left(\frac{r_{cm}^{2}}{r^{2}}\right), & r\geq r_{cm}\\ \frac{\omega_{0}\chi_{m}}{2}\left({\text{cos}}^{2}\;\theta_{m}-\frac{1}{3}-{\text{sin}}^{2}\;\theta_{m}\text{cos}\;2\phi \left(\frac{r_{cm}^{2}-r_{Lm}^{2}}{r^{2}}\right)\right), & r_{Lm}\leq r<r_{cm}\\ 0, & r<r_{Lm} \end{array},$$

where *ω*_0_ is the Larmor frequency, *θ*_*m*_ is the polar angle between the long axis of the cylinder *m* and *B*_0_, and the coordinates *ϕ*, *r* represent position in a cylindrical system with the *z*-axis parallel to the cylinder long axis and *B*_0_ defined in the *xz* plane. *χ*_*m*_ is the susceptibility difference (with the susceptibility tensor assumed isotropic) between the wall of the cylinder and outside, *r*_*cm*_ is the cylinder outer radius, and *r*_*Lm*_ the lumen radius of cylinder *m*. The summation is taken over all cylinders, each being indexed by *m*.

The diffusion tensor field is treated such that each cylinder lumen has its own diffusion tensor, each cylinder wall has its own diffusion tensor and the surroundings have a unique diffusion tensor. The *b*-tensor field may be represented as a sum over perturbers for each region as

(7) $$b=\sum_{m}b^{\left(m\right)}=b_{A}+b_{B}+b_{C}+b_{E}+b_{F}+b_{G}.$$

In this “alphabet” of terms, we recognize *A* and *B* as the dephasing due to the system’s response to the applied field only (due to susceptibility differences), *C*, *E*, and *F* as the dephasing due to the applied field gradients, and *G* and *H* due to the interaction between those two phenomena. To simplify proceedings, we impose the condition that the diffusion tensor outside the perturbers is isotropic, and that the diffusion tensor for each perturber wall and lumen is axially symmetric with its unique axis parallel to that particular perturber’s axis. Then we obtain

(8) $$b_{A}^{\left(m\right)}\cdot D_{out}^{\left(m\right)}=\frac{\rho^{2}t^{3}}{3}\frac{\chi_{m}^{2}\omega_{0}^{2}r_{cm}^{4}{\text{sin}}^{4}\;\theta_{m}}{r^{6}}D_{out},$$

where $D_{out}$ is the isotropic diffusion coefficient of the space outside any perturbers. We also obtain

(9) $$b_{B}^{\left(m\right)}\cdot D_{out}^{\left(m\right)}=\frac{\rho^{2}t^{3}}{3}\frac{\chi_{m}^{2}\omega_{0}^{2}{\left(r_{cm}^{2}-r_{Lm}^{2}\right)}^{2}{\text{sin}}^{4}\;\theta_{m}}{r^{6}}D_{wall,R}^{\left(m\right)},$$

where $D_{wall,R}^{\left(m\right)}$ is the radial diffusivity in the wall of perturber *m*, and

(10) $$b_{G}^{\left(m\right)}\cdot D_{out}^{\left(m\right)}=\frac{2\rho^{2}t^{3}\gamma \omega_{0}}{3}\frac{\chi_{m}r_{cm}^{2}{\text{sin}}^{2}\;\theta_{m}}{r^{3}}D_{out}\left(G_{x}\text{cos}\;3\phi +G_{y}\text{sin}\;3\phi \right),$$

(11) $$b_{H}^{\left(m\right)}\cdot D_{lumen}^{\left(m\right)}=\frac{2\rho^{2}t^{3}\gamma \omega_{0}}{3}\frac{\chi_{m}\left(r_{cm}^{2}-r_{Lm}^{2}\right){\text{sin}}^{2}\;\theta_{m}}{r^{3}}D_{lumen,R}^{\left(m\right)}\left(G_{x}\text{cos}\;3\phi +G_{y}\text{sin}\;3\phi \right).$$

The *C*, *E*, and *F* terms are the same as in conventional treatments (applied field gradients only) and may be found in the Supporting Material, as well as more general expressions.

This theory predicts a sin^4^ *θ* dependence for diffusion-mediated decoherence due to susceptibility differences and a sin^2^ *θ* dependence for the interaction between susceptibility differences and applied field gradients. For a voxel in which perturbers share a common axis of alignment (such as through which a single WM fiber tract passes), and in the absence of “significant” effects of applied field gradients, we can therefore anticipate an anisotropy of spin-echo *R*_2_, scaled simply by ${\text{sin}}^{4}\;\theta$, with θ the common angle between fiber and *B*_0_ as

(12) $$S\left(t\right)\approx \int A_{0}\;\text{exp}\left(-\alpha t^{3}\;{\text{sin}}^{4}\;\theta \right)\text{exp}\left(-R_{2}^{iso}t\right)dx.$$

Therefore we arrive at the following simple “semi-heuristic” expression for spin-echo *R*_2_:

(13) $$R_{2}=R_{2}^{iso}+A\;{\text{sin}}^{4}\;\theta ,$$

where the “amplitude of anisotropy” *A* depends on the set of echo times at which the signal is sampled (due to the cubic time dependence of signal decay). *A* is also scaled by the square of susceptibility differences between cylinder walls and surroundings, and the square of Larmor frequency (therefore applied field). ${\text{sin}}^{4}\;\theta$ may be approximated by calculating the angle between the principal eigenvector of the diffusion tensor and the applied magnetic field. We can equivalently express this in terms of *T*_*2*_ as

(14) $$T_{2}=\frac{T_{2}^{∥}}{1+AT_{2}^{∥}\;{\text{sin}}^{4}\;\theta },$$

where $T_{2}^{∥}=1/R_{2}^{iso}$ is the *T*_*2*_ parallel to *B*_0_. When *θ* = 90° we obtain the definition of the quantity $T_{2}^{⊥}$, from which we can define the “peak-to-trough” distance in *T*_*2*_ between parallel and perpendicular orientations as

(15) $$\begin{array}{c} T_{2}^{\Delta }=T_{2}^{∥}-T_{2}^{⊥}\\ =\frac{AT_{2}^{∥2}}{1+AT_{2}^{∥}} \end{array}.$$

## Materials and Methods

### Simulations

A set of classes were written to perform simulations of diffusion-mediated decoherence using MATLAB 2015b (The MathWorks, Natick, MA). To examine the combined effects of susceptibility differences and applied field gradients, we performed simulations using a geometry of a single walled cylinder. To examine the effects that crossing-fiber populations have on anisotropy of *T*_*2*_ and diffusion parameters in the presence of field inhomogeneities, we created a perturber geometry of 32 walled cylinders. Either all 32 were parallel, or 16 were grouped and parallel in one direction, and the other 16 were parallel but grouped at an orientation 90° to the first group. In all cases, simulations were performed without applied field gradients (to determine *T*_*2*_ anisotropy) and with six noncollinear gradients to explore the effect on diffusion tensor parameters. Complete simulation parameters are available in the Supporting Material.

### Experimental MRI

A total of 40 participants were recruited for this study (25 females, aged 23–71). They were required to have no known neurological disorder, past or present. All participants gave informed consent, and ethical approval was granted by the University of Bristol Faculty of Science Research Ethics Committee. All data were acquired using a Siemens Magnetom Skyra 3T system (Siemens Healthcare, Erlangen, Germany) equipped with a 32-channel head coil 2-channel parallel transmit body coil. The acquisition included a three-dimensional (3D) T_1_-weighted MPRAGE (sagittal, 0.86 × 0.86 × 0.86 mm^3^), two-dimensional (2D) multiecho spin-echo (axial, 1.15 × 1.15 × 1.98 mm^3^) and 2D multiband diffusion tensor imaging (DTI) (16) (axial, 1.88 × 1.88 × 1.98 mm^3^). Complete acquisition parameters are listed in the Supporting Material.

*T*_*2*_ maps were computed by a voxel-wise fit of a monoexponential function in a logarithmic space, excluding the first echo. This was done since the pulse sequence allows the passage of both spin and stimulated echoes due to the use of identical crusher gradients astride each refocusing pulse, though the first echo contains only spin echo contributions.

Diffusion tensor images were computed using FMRIB Software Library (FSL). Distortions caused by eddy currents were minimized using the program eddy (17), and gross distortions due to interfaces between materials with different magnetic susceptibility corrected with the program topup (18), before fitting diffusion tensors with dtifit. A single effective diffusion tensor was assumed for each voxel.

For the determination of age-dependent effects in the major WM tracts, the tract-based spatial statistics (TBSS) framework was used to identify a WM skeleton, implemented in FSL (19, 20). Fractional anisotropy (FA) images were registered to the FMRIB58_FA standard template and the FA skeleton determined at a threshold of 0.2 after which the radial diffusivity (RD), mean diffusivity (MD), axial diffusivity (AxD), and *T*_*2*_ maps were also skeletonized using the tbss_non_FA command.

### Analysis of *T*_*2*_ anisotropy

Anisotropy of *T*_*2*_ was examined by two approaches. First, we used the method we have previously published to provide a heuristic demonstration as a surface plot of *T*_*2*_ as a function of FA and the angle *θ* (between the principal direction of diffusion and *B*_0_). In this method, FA and *θ* are bin-ranged to create 2D bins. All *T*_*2*_ observations falling into a bin are averaged. A surface plot may be thereby produced. Data are required in a common space, chosen for each participant as that of their DTI data, resampled to 1 mm isotropic resolution.

In the second approach, we created a regression model that could be fitted to the data, motivated by the theory presented in this article and our recent work. It was more practical to work with *R*_2_ than *T*_*2*_, as *R*_2_ terms are effectively additive and linear in the anisotropy effect. A “full” model was constructed, modeling the effects of FA, MD, and age upon *R*_2_ up to second-order polynomials and anisotropy to first-order ones (the latter as per the theory section). All interaction terms were retained. A “reduced” model was also used, which did not include any *T*_*2*_ anisotropy terms, and compared with the full model. The full model was also used to examine differences between early-myelinating and late-myelinating WM fiber tracts. Tracts of the Johns Hopkins University (JHU) WM atlas (21, 22) were classified simply as “late-myelinating” or “early-myelinating” according to whether they have detectable levels of myelination at birth (23). Each voxel of the TBSS skeleton was therefore given such a label according to the most probable tract as identified by the JHU atlas, and the regression model applied separately for the two groups of data. Before fitting, data were demeaned and normalized by standard deviation (converted to *z*-scores), as the variables are on different scales. The regression analyses used the LinearModel class of MATLAB 2015b. Full expressions are given in the Supporting Material.

## Results

### Interaction between system interactions and applied field gradients

**Figure 1:**
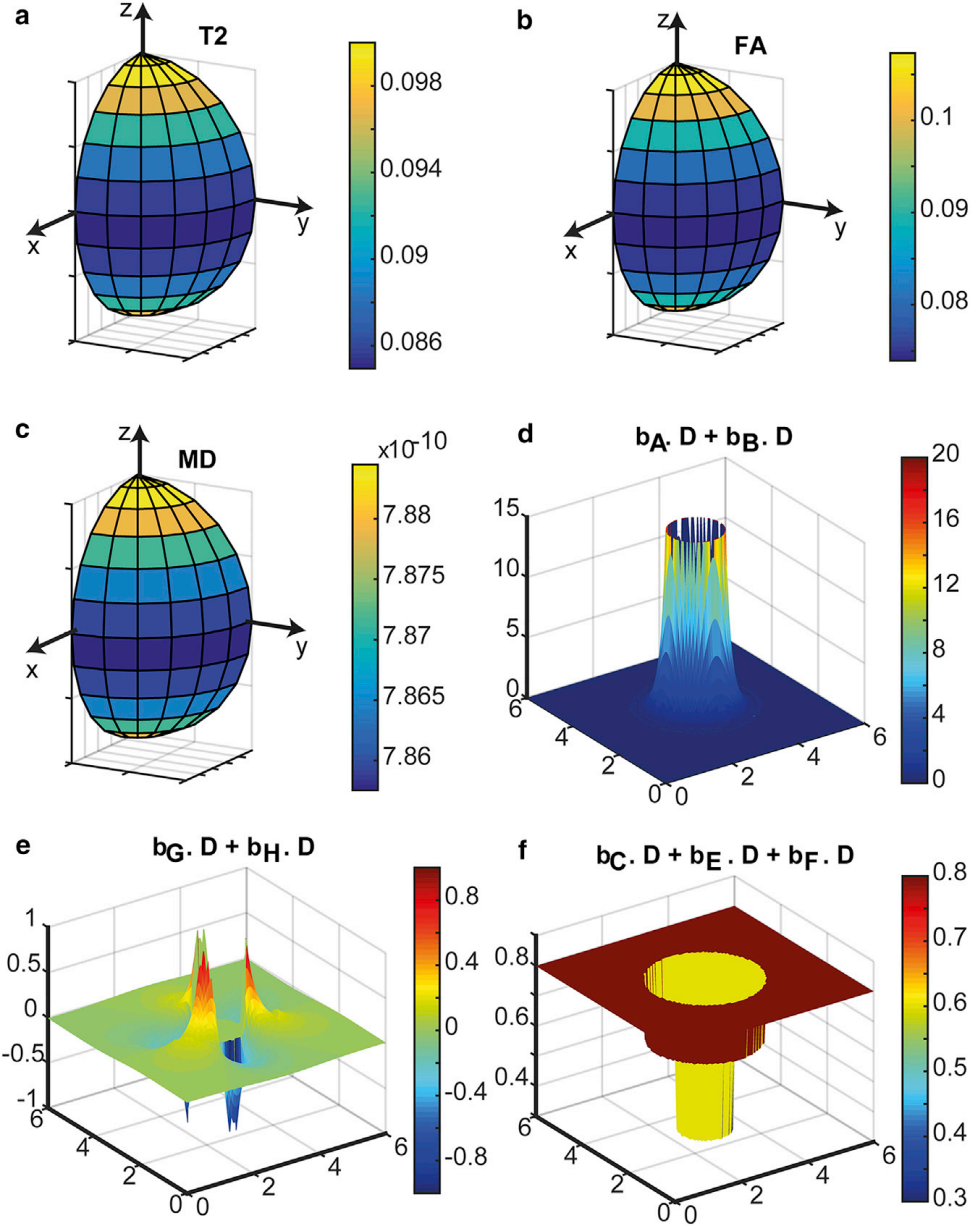
The effects of diffusion-mediated dephasing in the presence of susceptibility differences and applied field gradients for a single cylinder parallel to the *z* axis. (*a*)–(*c*) show the *T*_*2*_ (scale bar units s), FA (scale bar unitless), and MD (scale bar m^2^ s^−1^) respectively simulated for various polar and azimuthal angles relative to *B*_0_. (*d*)–(*f*) show the products of the *b*-tensor field and the diffusion tensor field for the three categories of dephasing. (*d*) shows the effects of susceptibility differences only, (*e*) shows the interaction between susceptibility differences and an applied field gradient parallel to the *x* axis, and (*f*) shows the effects of the applied field gradient parallel to *x* only. Note that different scales are used in each panel. To see this figure in color, go online.

The results of simulations for a single “thick-walled” cylinder are shown in Fig. 1. In Fig. 1, *a*–*c*, the *T*_*2*_ as a function of orientation are shown. The *T*_*2*_ expresses the anticipated orientation dependence, with its minimum when the cylinder is perpendicular to *B*_0_, for at such an orientation the magnetic field is rendered most inhomogeneous if the wall has a different susceptibility from the surroundings. Accordingly, the broadest distribution of resonance frequencies is sampled by each spin, and so decoherence most severe. The observable FA for the system is reduced perpendicular to *B*_0_ (Fig. 1 *b*), while MD is increased (Fig. 1 *c*). This is because dephasing in the vicinity of the wall is increased when perpendicular to *B*_0_, giving the impression of increased diffusivity in all directions. This, of course, increases observable MD, but also decreases normalized differences between eigenvalues of the diffusion tensor, and therefore decreases the observable FA. The interaction terms between the applied field gradients and diffusion-mediated decoherence due to susceptibility differences have only a small influence, slightly reducing the overall rate of decoherence. By inspection of Fig. 1 *e*, such terms may be positive or negative, so once averaged over the domain of simulation, the effects are (somewhat) suppressed.

### The effects of crossing fibers on *T*_*2*_ anisotropy

**Figure 2:**
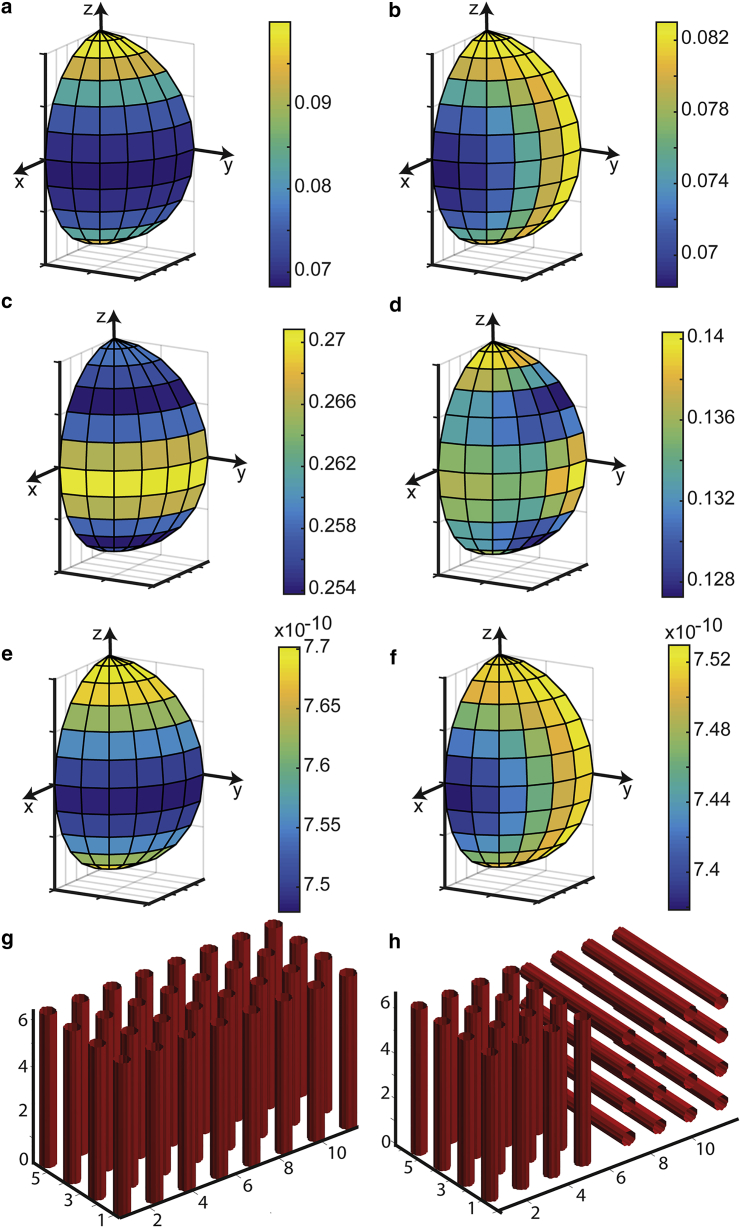
Simulations of diffusion-mediated decoherence, and its effects on *T*_*2*_ and diffusion tensor parameters for a single-fiber population and crossing fibers at 90°. (*a*), (*c*), and (*e*) show simulations in the single-fiber case; (*b*), (*d*), and (*f*) show simulations in the crossing-fiber case. (*a*) and (*b*) show *T*_*2*_ on a sphere (scale bar units: s), (*c*) and (*d*) show FA on spheres (scale bar unitless), (*e*) and (*f*) show MD on spheres (scale bar units: m^2^ s^−1^), and (*g*) and (*h*) show the perturber geometry. In (*g*) and (*h*), coordinates are given in units of micrometer. To see this figure in color, go online.

We compare the results of simulating diffusion-mediated decoherence for a single-fiber population and crossing-fiber system in Fig. 2. For the single-fiber population, the anisotropy of *T*_*2*_ follows the familiar pattern of depending only on the angle between the longitudinal axis of the perturbers and *B*_0_ (Fig. 1 *c*). For the crossing-fiber system, both the polar and azimuthal angle between the system of perturbers and *B*_0_ contribute to the *b*-tensor field. The *T*_*2*_ is minimized when both sets of perturbers are perpendicular to *B*_0_, which occurs at *θ* = 90°, *ϕ* = 0°. *T*_*2*_ is maximized when either population is parallel to *B*_0_, but the other is then perpendicular so *T*_*2*_ remains lower than the single-fiber system. The maximum *T*_*2*_ in the crossing-fiber system is therefore lower than in the single-fiber system.

Examining the FA in the single-fiber case, it is maximized when the perturbers are perpendicular to *B*_0_ (Fig. 2 *c*), though its minimum is not at the parallel orientation. Examining MD (Fig. 2 *e*), it follows a similar anisotropy to *T*_*2*_ (in the noncrossing case). This is for the same reasons as in the single-cylinder case. The main reason for which FA is reduced in the crossing fiber relative to the single-fiber case is of course the lack of a unique axis of order. The anisotropy of FA in this case is rather complicated, but again we see FA maximized (raised above the “true” value) if one or the other set of perturbers is perpendicular to *B*_0_ (Fig. 2 *d*). The MD anisotropy (Fig. 2 *f*) is very similar to the *T*_*2*_ anisotropy, the MD being reduced from its true value when the contribution to dephasing from all contributions to the *b*-tensor field is maximized. This is at *θ* = 90°, *ϕ* = 0°. In the Supporting Material, we provide similar plots to Fig. 2, for fiber crossing angles other than 0° and 90°.

### Experimental demonstration of *T*_*2*_ anisotropy

**Figure 3:**
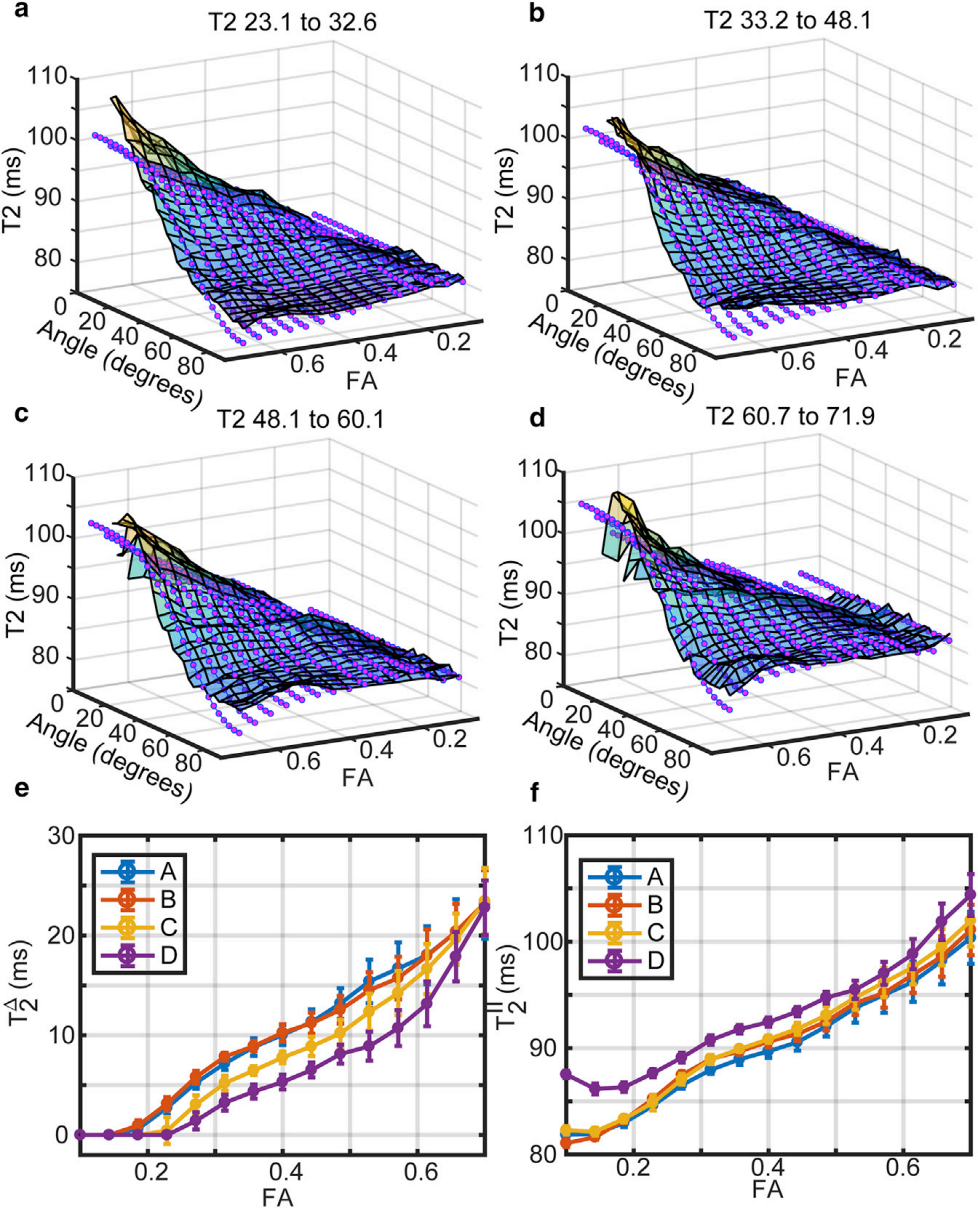
Experimental demonstration of *T*_*2*_ anisotropy in human brain white matter. (*a*)–(*d*) show surfaces of the average *T*_*2*_ in 2D bins according to FA and the angle between the principal axis of the diffusion tensor and *B*_0_ in four age groups indicated above the panels. The opaque surfaces are the experimental observations; the dots the fit of Eq. 13. A general tendency toward increased *T*_*2*_ and a decreased effect of anisotropy with increasing age is visible. (*e*) and (*f*) show the fitted $T_{2}^{\Delta }$ and parallel (isotropic) $T_{2}^{∥}$ derived from fitting Eq. 13 at each FA bin-range center value. Error bars are the 95% confidence intervals for the fit. The legend entries A, B, C, and D correspond to the respective age groups of (*a*)–(*d*). To see this figure in color, go online.

In Fig. 3, the results of a heuristic approach to extracting the effect of *T*_*2*_ anisotropy are shown, along with the fit of Eq. 13. In this analysis, participants were grouped into four age ranges with 10 participants in each subgroup. Therefore, any variance in the data because of factors such as age range and MD is absorbed by averaging over many observations at each selected 2D bin of θ and FA. There is a clear effect of anisotropy, consistent with our previous findings, with high FA (a high degree of order) corresponding to a high degree of *T*_*2*_ anisotropy. We can also see that the entire surface plot shifts up with age (*T*_*2*_ increases generally with age in WM), though the “peak-to-trough” (effect of anisotropy) decreases with increasing age, implying an increase in the isotropic *T*_*2*_ with age. The peak-to-trough distance is quantifiable, giving a convenient parameterization of the overall “effect of anisotropy.” This quantity, $T_{2}^{\Delta }$, is plotted in Fig. 1 *e*. In the youngest age group of 23.1–32.6 years, for the FA bin range 0.379–0.421, it has the value 10.0 ± 1.02 ms, whereas in the oldest group of 60.7–71.9 years, it is 5.31 ± 0.75 ms. In the FA bin range 0.592–0.654, it has the value 18.02 ± 2.91 ms in the youngest age group and 13.14 ± 2.24 in the oldest. The isotropic (parallel) $T_{2}^{∥}$ is plotted as a function of FA in Fig. 3 *f*, showing its increase with age and FA. It is seen that in total, at high FA in particular, *T*_*2*_ varies by up to 20 ms with θ; the angle between the principal direction of translation diffusion and *B*_0_. The effect of anisotropy may therefore explain a significant amount of variance in the overall distribution of *T*_*2*_ for WM.

### A regression model for *T*_*2*_: Demonstration of anisotropy

**Figure 4:**
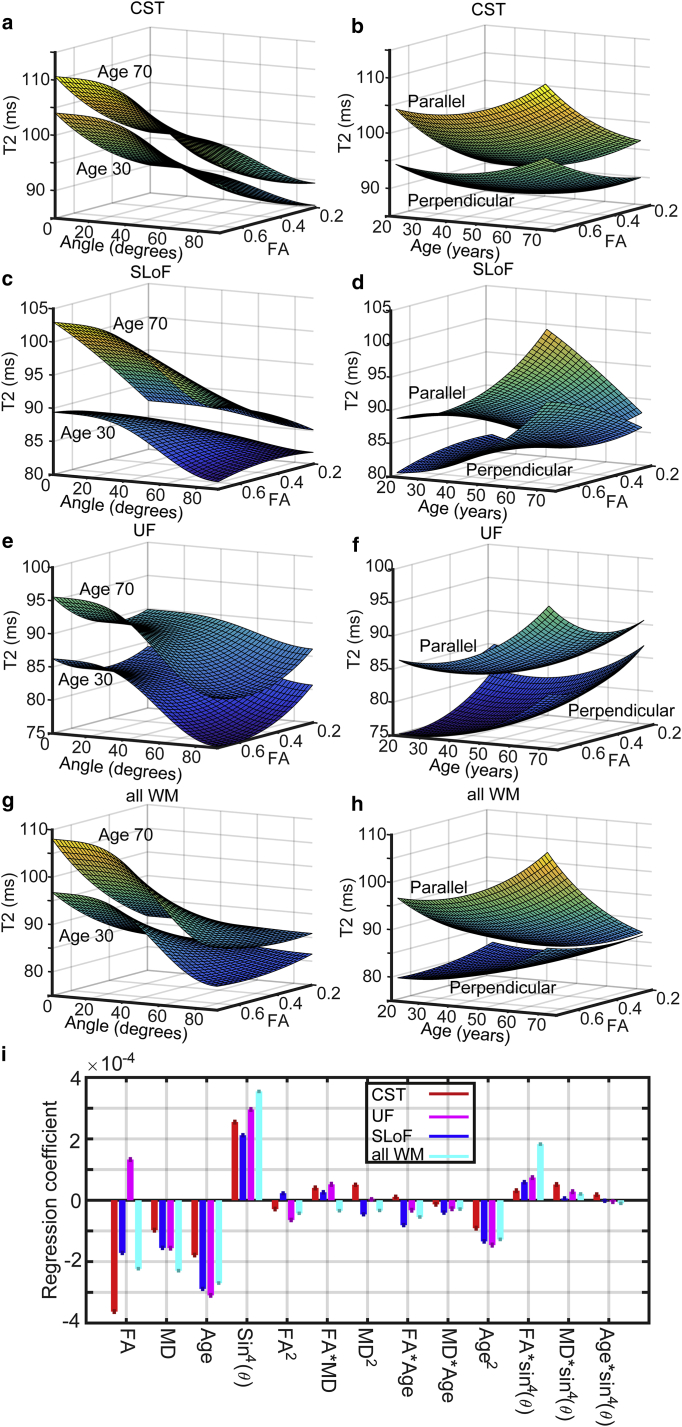
A regression model for *T*_*2*_ in a cohort of healthy persons. The model used FA, MD, age, and sin^4^*θ* as explanatory variables of *R*_2_ but is plotted in terms of *T*_*2*_. The model shows predictions of *T*_*2*_ for a fixed value of MD of 0.78 × 10^−9^ m^2^ s^−1^ (the median across the data set). (*a*), (*c*), (*e*), and (*g*) show the dependence on FA and *θ* (angle), the upper surface at an age of 70, and the lower at an age of 30 (as indicated on each panel). (*b*), (*d*), (*f*), and (*h*) show the dependence of *T*_*2*_ on FA and age at angles of 0 (*top surface*) and 90° (*lower surface*), labeled parallel and perpendicular, respectively. (*a*) and (*b*) are for the CST, (*c*) and (*d*) are for the SLoF, (*e*) and (*f*) are for the UF, and (*g*) and (*h*) are for anything simultaneously within the WM skeleton and JHU WM atlas. (*i*) shows the regression coefficients (fitted as *z*-scores) for each term in the model and for each region. To see this figure in color, go online.

To more thoroughly examine interactions between factors such as age, MD, etc., and perform a statistical test of whether anisotropy contributes to our data we focused our attention on three major WM tracts: the corticospinal tracts (CSTs), which are early-myelinating association fibers; the superior longitudinal fasciculus (SLoF), containing later-myelinating fibers (but close to the CST), and the uncinate fasciculus (UF), containing late-myelinating fibers but in an anatomically distinct region and consistently implicated as suffering early change in dementia and cognitive decline (24). In all cases, a model including an effect of *T*_*2*_ anisotropy was better able to describe the data than a model without, with the *p*-values for the amplitude of anisotropy and many interaction terms involving it zero to the limit of machine precision. Summary statistics are tabulated in the Supporting Material (Table S1). A key result is that *T*_*2*_ anisotropy declines with increasing age and increases with increasing FA, which is consistent with Fig. 3. An exception, however, was the UF, in which an interaction between age and anisotropy could not be detected (*p* > 0.05 for the interaction term), such that in this tract *T*_*2*_ anisotropy is less affected by age. Therefore, *T*_*2*_ anisotropy also changes differently with age depending on brain region or WM tract. A visualization of the model is provided in Fig. 4. The effect of anisotropy is clear, reflected in the high statistical significance for its inclusion and regression coefficients for the amplitude of anisotropy shown in Fig. 4 *i*. For example, the model describes the data for the entire WM skeleton with an *R*_2_ of 0.27, compared with 0.14 without an effect of anisotropy modeled, with the regression coefficient for the amplitude of anisotropy (shown in Fig. 4 *i*) the largest of all terms (because the data were transformed to *z*-scores, coefficients are on a comparable scale). Like the experimental form (averaging over MD and bin-ranged over FA), the effect increases with increasing FA and decreases with increasing age. It is clear that the variation in *T*_*2*_ explained by anisotropy is similarly significant to that explained by age. The minimum *T*_*2*_ is typically passed at age ∼30 (though varies a little according to interaction effects).

### Demonstration of faster aging and lower anisotropy in late-myelinating tracts

**Figure 5:**
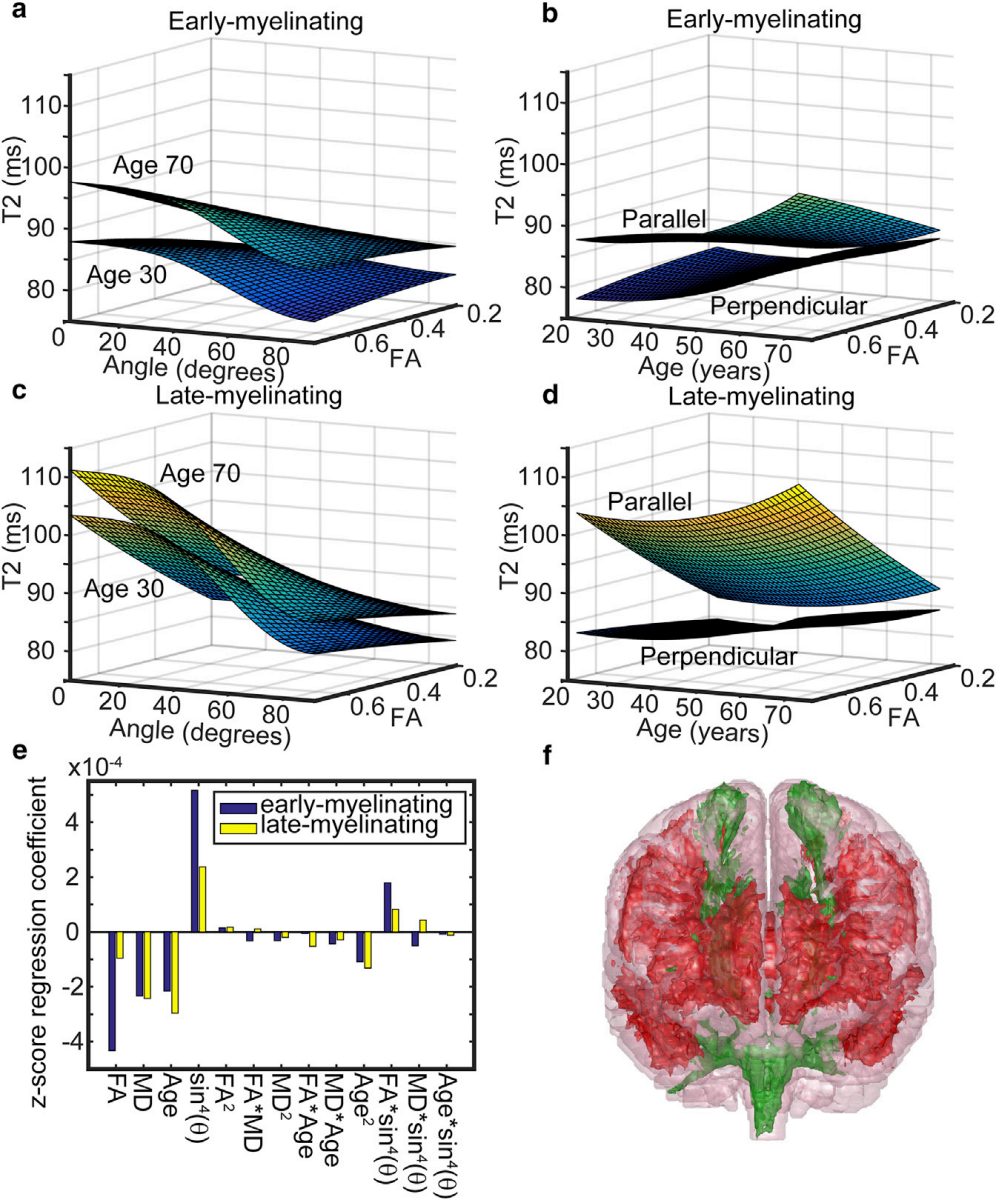
Regression model for *T*_*2*_ separated into late-myelinating and early-myelinating regions of the TBSS-identified WM skeleton. The form of the fitted model is plotted at a constant MD of 0.78 × 10^−3^ mm^2^ s^−1^ (the median across the data set). (*a*) and (*c*) show the dependence on FA and *θ* (angle), the upper surface at an age of 70 years, and the lower at an age of 30 years for early- and late-myelinating WM, respectively. (*b*) and (*d*) show the dependence of *T*_*2*_ on FA and age at angles of 0 (*top surface*) and 90° (*lower surface*) for early- and late-myelinating WM, respectively. (*e*) shows fitted regression coefficients (the data were first demeaned and scaled by standard deviation). (*f*) shows the locations of the early- (*green* in online version) and late-myelinating (*red* in online version) regions, as modeled for the analysis. To see this figure in color, go online.

Each voxel of the WM skeleton was labeled as early-myelinating or late-myelinating, to create two data sets. By fitting the regression model including the effect of *T*_*2*_ anisotropy to these two data sets separately, we were able to examine differences between the aging characteristics of the two classes of WM. The results are shown in Fig. 5. From these models, and in particular inspecting the regression coefficients in Fig. 5 *e*, we can make several observations. First, although in both WM groups the *T*_*2*_ increases with age, the effect of age is greater in the late-myelinating WM. This is so for the first- and second-order coefficients. Therefore, *T*_*2*_ increases with age more rapidly in late-myelinating WM, especially in later life. Second, the effect of anisotropy is markedly larger in the early-myelinating WM. As such, the microstructure conferring the property of anisotropy upon *T*_*2*_ is more prevalent or better-preserved with age in early-myelinating WM.

## Discussion

We have developed a formalism of nuclear spin phase decoherence due to mesoscopic magnetic field inhomogeneities and explored the effects on measurements of diffusion and relaxation anisotropy.

### Relaxometry in microstructure imaging

The fact that *T*_*2*_ is influenced by fiber orientation and the presence of crossing fibers may provide another domain in which relaxometry can contribute to microstructure imaging. Knowledge of the response of *T*_*2*_ (or other relaxometric parameters) to applied field gradients and its dependence on microstructure and orientation may provide the basis for novel microstructural imaging modalities and restraints in testing experimental models. Recent work has already begun to explore the utility of other relaxometry modalities in microstructure imaging. In particular, it has been shown that T_1_ relaxometry is able to quantify differential WM tract characteristics (25), including unique T_1_ values for each fiber of crossing fiber populations (26).

We showed by two means that there is an effect of orientation on a voxel’s *T*_*2*_ in human WM, and that the effect is consistent with the theory provided. Therefore, a model of cylindrical field perturbers creating mesoscopic magnetic field inhomogeneities appears to be suitable for describing *T*_*2*_ measured using a multiecho spin-echo pulse sequence in the human brain in vivo. The use of such a model extends our previous observation (12), and extends the use of a cylindrical field perturber model in frequency difference mapping (5) and QSM (6).

We have also shown an interaction between age and anisotropy. As we age, the extent to which anisotropy influences *T*_*2*_ decreases. Therefore, we anticipate that contributors to mesoscopic magnetic field inhomogeneities are removed with age. Exactly what those contributors are is a largely unexplored field. The effects of age and anisotropy upon *T*_*2*_ are not just tract-specific but depend on myelogenesis. We have shown that early-myelinating WM, at least within the major fiber bundles, is more “robust” to aging than late-myelinating WM when parameterized by *T*_*2*_. Specifically, *T*_*2*_ increases more rapidly in this cross-sectional cohort with age in late-myelinating WM, accelerating even more with age, while its anisotropy is less than that of early-myelinating WM. There is also a weaker interaction between age and *T*_*2*_ anisotropy in early than late-myelinating WM. This is suggestive that *T*_*2*_ mapping and the development of modalities for the mapping of its anisotropy may be powerful means of examining subtle differences in the “types” of aging that distinct categories of WM undergo. This is significant in the context of the retrogenesis hypothesis, which posits that late-myelinating WM is the most susceptible to damage in later life. There is evidence from a number of studies using DTI scalars and tractography that this is the case, and the hypothesis may also explain the disproportionate damage to WM observed in Alzheimer’s disease, which may precede significant loss of gray matter. The observation of an increased rate of age-related change in *T*_*2*_ of late-myelinating WM in a healthy population may therefore suggest a capacity to detect “silent” pathology before clinical presentation. The same may be true of the detection of larger interactions between age and anisotropy (driven by microstructure) in late-myelinating WM.

Brain aging at the microstructural level is not well understood (27). A number of studies have used DTI to monitor the changes in DTI scalars with age (28). Overall, DTI data converge on widespread decreases in FA in WM with age and widespread increases in diffusivities, after a peak is passed in the third or fourth decade of life, and with intertract differences (29, 30, 31). Consistent with the theory of retrogenesis (24), there is some evidence that early-myelinating WM shows slower rates of decline when parameterized by DTI scalars. Longitudinal data similarly demonstrate widespread but subtle WM microstructural changes with age, and not explicable by loss of cortical gray matter (GM) (32, 33). This is significant, for despite a shortage of empirical data (34) it has been suggested that GM loss may be causative of WM microstructural change (35), such as through Wallerian degeneration. *T*_*2*_^∗^ relaxometry has also been applied to characterize age-related changes in the brain, showing decreases with age in various subcortical gray matter structures likely to generally increase in iron content with age, and limited WM regions (36). In another study, focal *T*_*2*_^∗^ increases and decreases were seen in various WM regions (37).

### Relation to *T*_*2*_^∗^ and other literature

The effect of anisotropy is a weak one, scaling with the square of *B*_0_, and requires some level of care to measure. Either many observations must be averaged as in the surface plots of Fig. 3 or a regression model must be fitted to a data set of appreciable size. In addition, it is likely that poor *B*_1_ and *B*_0_ homogeneity will confound its measurement. With these considerations, we might account for why the effect has received scant attention. A previous study sought to detect anisotropy in ex vivo bovine optic nerve at 1.5 T but reported no anisotropy (38). They did, however, report that decoherence was more rapid at early times, as predicted by our model if anisotropy be present, but used a multiexponential fit rather than Eq. 12.

### Future applications

We have sought primarily to describe and explain the biophysical phenomenon of coherence lifetime anisotropy due to restricted translational diffusion through inhomogeneous magnetic fields created by biological tissues on mesoscopic (cellular) scales. There are several opportunities for exploiting such a phenomenon in both basic research and clinical applications, if routine measurement can be brought into reality. The key applications we foresee are those involving “widespread but subtle” change on a cellular scale, in which pathology is not highly localized, or does not perturb some MR-observable parameter to such an extent as to place it within the detection limit of the human visual system. Examples may include various classes of dementia, or recovery/adaptation following stroke. We may realistically hope to provide quantitative markers of cellular-level tissue change in advance of tissue death, thus bringing forward the window of opportunity for detecting disease pathology. It may also be possible to refine estimates of quantities such as axonal packing or diameters, thereby monitoring both generation and degeneration of WM. This may be useful in determining the efficacy of some treatment (and thus guiding treatment on an individualized basis) as well as providing quantitative biomarkers useful in the development of new therapeutics aimed at preventing axonal degeneration or promoting axonal generation. This, we hope, will follow a similar pathway to application as other phenomena occurring in, or measurable by, magnetic resonance, such as diffusion anisotropy, magnetic susceptibility (and its anisotropy) and perfusion.

### Limitations

Constructing a meaningful microstructure-driven model for *T*_*2*_ is similarly challenging to doing so for any other parameter observable by MRI. The model is surely incomplete. However, there have been relatively few attempts at determining the effects of microstructure upon spin-echo *T*_*2*_, which justifies to some extent the choice of a simple model. We were limited in experimental data by using a cross-sectional cohort. However, seeking to determine the effects of age across a broad range (49 years) does not lend itself easily to longitudinal studies, which will be necessary to fully test the predictions emerging from this work. The data acquisition and processing also suffered imperfections. The *T*_*2*_ mapping was monoexponential and only made use of echoes recorded from 24 to 120 ms, the first at 12 ms being discarded since the pulse sequence used the same crushers astride each refocusing pulse, the first echo therefore being a “pure” spin echo and disproportionately low in intensity. However, this means that we are sensitive only to relatively slow-decaying coherence. This means we could not fit Eq. 12 directly to data. Neither could we fit models in which the effects of susceptibility differences are assumed not to contribute but the isotropic *T*_*2*_ is assumed to be different in the vicinity of the myelin sheath, such as multiexponential decoherence models. We have yet to address experimentally the issue of crossing fibers, instead simply limiting the analysis to the major fiber bundles identified by TBSS (in which crossing fibers are still likely to be a confound).

## Conclusions

In conclusion, it is most likely that the MRI *T*_*2*_ is influenced by microstructure, and we have proposed a means to make that influence tractable, adding *T*_*2*_ mapping to the range of microstructure imaging modalities. By understanding the physical basis of microstructural modulation of the MRI signal, we hope to make challenging matters of human health and disease tractable. As a demonstration, we have shown that the *T*_*2*_ and its anisotropy are differently affected by age, and that late-myelinating WM is more susceptible to the effects of age.

## Author Contributions

M.J.K. designed the experiments, collected and analyzed data, and wrote the manuscript. S.D. and L.J. collected the data. R.A.K. wrote the manuscript.

## Supporting Material

Document S1. Supporting Materials and Methods, Fig. S1, and Tables S1 and S2

Document S2. Article plus Supporting Material
