# Supplementary material for: Magnetic Resonance Relaxation Anisotropy: Physical Principles and Uses in Microstructure Imaging
Source: Biophys J. 2017 Apr 11;112(7):1517–28. doi: 10.1016/j.bpj.2017.02.026 (PMC5390049; doi:10.1016/j.bpj.2017.02.026)
Supplement: Document S1. Supporting Materials and Methods, Fig. S1, and Tables S1 and S2 [file mmc1.pdf]

Biophysical Journal, Volume *112*

Supplemental Information

Magnetic Resonance Relaxation Anisotropy: Physical Principles and Uses in Microstructure Imaging

Michael J. Knight, Serena Dillon, Lina Jarutyte, and Risto A. Kauppinen

Supplementary information

Mathematical details

We wish to calculate the form of the time-dependent spin phase decoherence due to generalised but small mesoscopic magnetic field inhomogeneities. The master equation is the Bloch-Torrey equation (1-3) for transverse magnetisation with a generalised but small magnetic field inhomogeneity(4):

$$\frac{\partial}{\partial t}M^{+}(t,\mathbf{x}) = \left(-i\omega_0 - i\omega_{cs} - i\omega_I(\mathbf{x}) - R_2(\mathbf{x}) + \nabla\cdot\mathbf{D}(\mathbf{x})\nabla\right)M^{+}(t,\mathbf{x}) \tag{1}$$

Here, $M^{+}(t,\mathbf{x})$ is the complex-valued transverse magnetisation as a function of time *t* and spatial coordinate **x**, ω_0 is the Larmor frequency, ω_{cs} is the isotropic part of the chemical shift anisotropy tensor, $\omega_I(\mathbf{x})$ is a frequency inhomogeneity function, $R_2(\mathbf{x})$ is the (isotropic) transverse relaxation rate coefficient scalar field, ∇ is the gradient operator, and $\mathbf{D}(\mathbf{x})$ the translational diffusion tensor field. In our original paper, we showed that the signal, in a demodulated frame rotated with the chemically shifted Larmor frequency, would evolve according to (2)

$$S(t) = \left|\int A_0(\mathbf{x})\exp\left\{\frac{i\rho t^2}{2}\left[\nabla\cdot\mathbf{D}(\mathbf{x})\nabla\right]\omega_I(\mathbf{x})\right\}\exp\left\{-\frac{\rho^2 t^3}{3}\left[\nabla\omega_I\cdot\mathbf{D}(\mathbf{x})\nabla\right]\omega_I(\mathbf{x})\right\}\exp\left(-R_2(\mathbf{x})t\right)d\mathbf{x}\right|$$

If we are performing a spin-echo experiment, assuming that phase terms may be entirely refocussed, this reduces to

$$S(t) = \left|\int A_0(\mathbf{x})\exp\left\{-\frac{\rho^2 t^3}{3}\left[\nabla\omega_I(\mathbf{x})\cdot\mathbf{D}(\mathbf{x})\nabla\right]\omega_I(\mathbf{x})\right\}\exp\left(-R_2(\mathbf{x})t\right)d\mathbf{x}\right| \tag{3}$$

Where, in both cases, ρ is the coherence order and A_0 the signal amplitude at t=0. We will henceforth restrict the discussion to spin-echo experiments and neglect phase terms.

We can express this more simply as

$$S(t) = \left|\int A_0 \exp\left(-\mathbf{b}\cdot\mathbf{D}\right)\exp\left(-R_2 t\right)d\mathbf{x}\right| \tag{4}$$

In which the b-tensor field **b** has been introduced. The quantity **b.D** represents the sum of element-wise products evaluated at coordinate **x**. The b-tensor field is defined as

$$b_{jk}(\mathbf{x}) = \frac{\rho^2 t^3}{3}\frac{\partial \omega_I(\mathbf{x})}{\partial x_j}\frac{\partial \omega_I(\mathbf{x})}{\partial x_k} \tag{5}$$

In which $\omega_I(\mathbf{x})$ is an inhomogeneous contribution to the resonance frequency experienced by the nuclear species under observation at coordinate **x**. This generalises the b-value used in diffusion imaging if the frequency inhomogeneity is linear:

$$b_{jk} = \frac{t^3}{3}\gamma^2 G_j G_k \tag{6}$$

With G_j, G_k elements of an applied (linear) magnetic field gradient.

Elements of the b-tensor field

The b-tensor expansion may be equivalently expressed:

$$\begin{aligned}\frac{\rho^2 t^3}{3}\left[\nabla\omega_I(\mathbf{x})\cdot\mathbf{D}(\mathbf{x})\nabla\right]\omega_I(\mathbf{x}) &= \frac{\rho^2 t^3}{3}\sum_m\sum_{j,k}\frac{\partial\omega_I^{(m)}(\mathbf{x})}{\partial x_j}\frac{\partial\omega_I^{(m)}(\mathbf{x})}{\partial x_k}D_{jk}^{(m)}(\mathbf{x})\\ &= \sum_m\sum_{j,k} b_{jk}^{(m)}(\mathbf{x})D_{jk}^{(m)}(\mathbf{x})\\ &= \sum_m \mathbf{b}^{(m)}\cdot\mathbf{D}^{(m)}\\ &= \mathbf{b}\cdot\mathbf{D}\end{aligned} \tag{7}$$

Or, as an expansion into the contributions of the susceptibility and applied field gradient effects:

$$\begin{aligned}\mathbf{b}\cdot\mathbf{D} &= \frac{\rho^2 t^3}{3}\sum_{j,k}\frac{\partial(\Delta\omega+\omega_D)}{\partial x_j}\frac{\partial(\Delta\omega+\omega_D)}{\partial x_k}D_{jk}\\ &= \frac{\rho^2 t^3}{3}\sum_{j,k}\left[\frac{\partial\Delta\omega}{\partial x_j}\frac{\partial\Delta\omega}{\partial x_k}+\frac{\partial\omega_D}{\partial x_j}\frac{\partial\omega_D}{\partial x_k}+\frac{\partial\Delta\omega}{\partial x_j}\frac{\partial\omega_D}{\partial x_k}+\frac{\partial\omega_D}{\partial x_j}\frac{\partial\Delta\omega}{\partial x_k}\right]D_{jk}\\ &= \frac{\rho^2 t^3}{3}\begin{bmatrix}\left[\nabla\Delta\omega\right]^T\cdot\mathbf{D}\nabla\Delta\omega\\ +\left[\nabla\omega_D\right]^T\mathbf{D}\nabla\omega_D\\ +\left(\left[\nabla\Delta\omega\right]^T\cdot\mathbf{D}\nabla\omega_D+\left[\nabla\omega_D\right]^T\cdot\mathbf{D}\nabla\Delta\omega\right)\end{bmatrix}\end{aligned} \tag{8}$$

Where the superscript *T* represents the transpose operation.

The cylindrical model

In the walled cylinder model, the frequency inhomogeneity function takes the form

$$\Delta\omega(\mathbf{x}) = \sum_m \begin{cases} \frac{\omega_0\chi_m}{2}\sin^2\theta_m \cos 2\phi\left(\frac{r_{cm}^2}{r^2}\right), & r \geq r_{cm} \\ \frac{\omega_0\chi_m}{2}\left(\cos^2\theta_m - \frac{1}{3} - \sin^2\theta_m \cos 2\phi\left(\frac{r_{cm}^2 - r_{Lm}^2}{r^2}\right)\right), & r_{Lm} \leq r < r_{cm} \\ 0, & r < r_{Lm} \end{cases} \quad (9)$$

Where ω_0 is the Larmor frequency, θ is the polar angle between the long axis of the cylinder *j* and B_0, and the coordinates ϕ ,*r* represent position in a cylindrical system with the z-axis parallel to the cylinder long axis and B_0 defined in the *xz* plane. This is the cylinder principal axis system (PAS). χ is the susceptibility difference (with the susceptibility tensor assumed isotropic) between the wall of the cylinder and outside, r_c is the cylinder outer radius radius, r_L the lumen radius. This frequency difference is present as long as the system is subject to an applied magnetic field B_0. The diffusion tensor field is treated such that each cylinder lumen has its own diffusion tensor, each cylinder wall (if applicable) has its own diffusion tensor and the surroundings have a unique diffusion tensor. We can therefore write

$$D_{jk}^{(m)} = \begin{cases} D_{jk}^{(m,out)}, & r \geq r_{cj} \\ D_{jk}^{(m,wall)}, & r_{Lj} \leq r < r_{cj} \\ D_{jk}^{(m,lumen)}, & r < r_{Lj} \end{cases} \quad (10)$$

Although we treat the region outside the cylinders as having a single diffusion tensor, it may be that the cylinders have different orientations, such that the mth representation is in a different frame (PAS of cylinder m). For the *b*-tensor field, this leads to the “alphabet” of terms:

$$\begin{aligned} \mathbf{b} &= \sum_m \mathbf{b}^{(m)} \\ &= \mathbf{b}_A + \mathbf{b}_B + \mathbf{b}_C + \mathbf{b}_E + \mathbf{b}_F + \mathbf{b}_G \end{aligned} \quad (11)$$

Where

$$
\begin{aligned}
\mathbf{b}_A &= \frac{\rho^2 t^3}{3}\sum_m \left[\nabla\Delta\omega_{out}^{(m)}\right]^T \nabla\Delta\omega_{out}^{(m)} \\
\mathbf{b}_B &= \frac{\rho^2 t^3}{3}\sum_m \left[\nabla\Delta\omega_{wall}^{(m)}\right]^T \nabla\Delta\omega_{wall}^{(m)} \\
\mathbf{b}_{C,E,F} &= \frac{\rho^2 t^3}{3}\sum_m \left[\nabla\omega_{D}^{(m)}\right]^T \nabla\omega_{D}^{(m)} \\
\mathbf{b}_G &= \frac{\rho^2 t^3}{3}\sum_m \left(\left[\nabla\Delta\omega_{out}^{(m)}\right]^T \nabla\omega_{D}^{(m)} + \left[\nabla\omega_{D}^{(m)}\right]^T \nabla\Delta\omega_{out}^{(m)}\right) \\
\mathbf{b}_H &= \frac{\rho^2 t^3}{3}\sum_m \left(\left[\nabla\Delta\omega_{wall}^{(m)}\right]^T \nabla\omega_{D}^{(m)} + \left[\nabla\omega_{D}^{(m)}\right]^T \nabla\Delta\omega_{wall}^{(m)}\right)
\end{aligned}
\tag{12}
$$

The full forms for the cylindrical model are:

$$
\mathbf{b}_A = \frac{\rho^2 t^3}{3}\sum_m \frac{\chi_m^2\omega_0^2 r_{cm}^4 \sin^4\theta_m}{r^6}\begin{bmatrix} \cos 2\phi & \cos 2\phi \sin 2\phi & 0 \\ \cos 2\phi \sin 2\phi & \sin 2\phi & 0 \\ 0 & 0 & 0 \end{bmatrix} \tag{13}
$$

$$
\mathbf{b}_B = \frac{\rho^2 t^3}{3}\sum_m \frac{\chi_m^2\omega_0^2 \left(r_{cm}^2 - r_{Lm}^2\right)^2 \sin^4\theta_m}{r^6}\begin{bmatrix} \cos 2\phi & \cos 2\phi \sin 2\phi & 0 \\ \cos 2\phi \sin 2\phi & \sin 2\phi & 0 \\ 0 & 0 & 0 \end{bmatrix} \tag{14}
$$

The b-tensor field for C,E,F is simply the b-value as used in the ordinary theory of diffusion-weighted imaging with the diffusion tensor represented for the appropriate compartment, so expressions may be found elsewhere.

$$
\mathbf{b}_G = \frac{\rho^2 t^3 \gamma\omega_0}{3}\sum_m \frac{\chi_m r_{cm}^2 \sin^2\theta_m}{r^3}\mathbf{W} \tag{15}
$$

$$
\mathbf{b}_H = \frac{\rho^2 t^3 \gamma\omega_0}{3}\sum_m \frac{\chi_m \left(r_{cm}^2 - r_{Lm}^2\right)\sin^2\theta_m}{r^3}\mathbf{W} \tag{16}
$$

Where

$$
\mathbf{W} = \begin{bmatrix} 2\cos 2\phi\left(G_x\cos\phi + G_y\sin\phi\right) & -\left(G_x\sin\phi + G_y\cos\phi\right) & G_z\cos 2\phi \\ -\left(G_x\sin\phi + G_y\cos\phi\right) & 2\sin 2\phi\left(-G_x\sin\phi + G_y\cos\phi\right) & G_z\sin 2\phi \\ G_z\cos 2\phi & G_z\sin 2\phi & 0 \end{bmatrix} \tag{17}
$$

These expressions are for cylindrical perturber geometry, but valid for any diffusion tensor. In the main text, we restricted the discussion to axially symmetric diffusion tensors with their unique axis parallel to that of the perturber to which they correspond, and isotropic diffusion outside the perturber region.

For computational efficiency, our simulator calculates the terms:

$$\begin{aligned}\frac{\rho^2 t^3}{3}\left[\nabla\omega_I\cdot\mathbf{D}\nabla\right]\omega_I &= \mathbf{b}\cdot\mathbf{D}\\ &= \frac{\rho^2 t^3}{3}\left(A+B+C+E+F+G+H\right)\end{aligned} \tag{18}$$

Where

$$\begin{aligned}
A &= \sum_m \nabla\Delta\omega_{out}^{(m)}\mathbf{D}_{out}^{(m)}\nabla\Delta\omega_{out}^{(m)}\\
B &= \sum_m \nabla\Delta\omega_{wall}^{(m)}\mathbf{D}_{wall}^{(m)}\nabla\Delta\omega_{wall}^{(m)}\\
C &= \nabla\omega_D\mathbf{D}_{out}\nabla\omega_D\\
E &= \sum_m \nabla\omega_D^{(m)}\mathbf{D}_{wall}^{(m)}\nabla\omega_D^{(m)}\\
F &= \sum_m \nabla\omega_D^{(m)}\mathbf{D}_{lumen}^{(m)}\nabla\omega_D^{(m)}\\
G &= \sum_m \left(\nabla\Delta\omega_{out}^{(m)}\mathbf{D}_{out}^{(m)}\nabla\omega_D^{(m)}+\nabla\omega_D^{(m)}\mathbf{D}_{out}^{(m)}\nabla\Delta\omega_{out}^{(m)}\right)\\
H &= \sum_m \left(\nabla\Delta\omega_{wall}^{(m)}\mathbf{D}_{wall}^{(m)}\nabla\omega_D^{(m)}+\nabla\omega_D^{(m)}\mathbf{D}_{wall}^{(m)}\nabla\Delta\omega_{wall}^{(m)}\right)
\end{aligned} \tag{19}$$

These correspond to $\mathbf{b}_A$ etc from the main text. Thus equipped, we may write out all the above dephasing terms fully as:

$$A=\sum_m \frac{\chi_m^2\omega_0^2 r_{cm}^4\sin^4\theta_m}{r^6}\left[\begin{array}{l}\left(2\cos^2\phi-1\right)^2\left(D_{11}^{(m)}\cos^2\phi+D_{22}^{(m)}\sin^2\phi+2D_{12}^{(m)}\sin\phi\cos\phi\right)+\\ 4\cos^2\phi\sin^2\phi\left(D_{11}^{(m)}\sin^2\phi+D_{22}^{(m)}\cos^2\phi-2D_{12}^{(m)}\sin\phi\cos\phi\right)+\\ 2\cos\phi\sin\phi\left(-D_{11}^{(m)}\frac{\sin2\phi}{2}+D_{22}^{(m)}\frac{\cos2\phi}{2}+D_{12}^{(m)}\cos2\phi\right)\end{array}\right] \tag{20}$$

Where the elements $D_{jk}^{(m)}$ are of the diffusion tensor for the region outside the perturbers in Cartesian representation transformed into in the “cylinder PAS” of perturber *m*.

$$B=\sum_m \frac{\chi_m^2\omega_0^2\left(r_{cm}^2-r_{Lm}^2\right)^2\sin^4\theta_m}{r^6}\left[\begin{array}{l}\left(2\cos^2\phi-1\right)^2\left(D_{11}^{(m)}\cos^2\phi+D_{22}^{(m)}\sin^2\phi+2D_{12}^{(m)}\sin\phi\cos\phi\right)+\\ 4\cos^2\phi\sin^2\phi\left(D_{11}^{(m)}\sin^2\phi+D_{22}^{(m)}\cos^2\phi-2D_{12}^{(m)}\sin\phi\cos\phi\right)+\\ 2\cos\phi\sin\phi\left(-D_{11}^{(m)}\frac{\sin 2\phi}{2}+D_{22}^{(m)}\frac{\cos 2\phi}{2}+D_{12}^{(m)}\cos 2\phi\right)\end{array}\right]$$

(21)

Where the elements $D_{jk}^{(m)}$ are of the diffusion tensor for the wall of perturber *m* in the cylinder PAS of perturber *m*, in Cartesian representation.

$$C=\gamma^2\left(D_{11}G_x^2+D_{22}G_y^2+D_{33}G_z^2+2D_{12}G_xG_y+2D_{13}G_xG_z+2D_{23}G_yG_z\right) \quad (22)$$

Where the diffusion tensor is for the region outside the perturbers, may have a single reference frame provided the pulsed field gradient G is also in that frame and is in Cartesian representation.

$$E=\gamma^2\sum_m\left(D_{11}^{(m)}G_x^2+D_{22}^{(m)}G_y^2+D_{33}^{(m)}G_z^2+2D_{12}^{(m)}G_xG_y+2D_{13}^{(m)}G_xG_z+2D_{23}^{(m)}G_yG_z\right) \quad (23)$$

Where the elements $D_{jk}^{(m)}$ are of the diffusion tensor for the wall of perturber *m* in the cylinder PAS of perturber *m*, in Cartesian representation. The pulsed field gradient **G** should also be represented in that frame.

$$F=\gamma^2\sum_m\left(D_{11}^{(m)}G_x^2+D_{22}^{(m)}G_y^2+D_{33}^{(m)}G_z^2+2D_{12}^{(m)}G_xG_y+2D_{13}^{(m)}G_xG_z+2D_{23}^{(m)}G_yG_z\right) \quad (24)$$

Where the elements $D_{jk}^{(m)}$ are of the diffusion tensor for the lumen of perturber *m* in the cylinder PAS of perturber *m*, in Cartesian representation. The pulsed field gradient **G** should also be represented in that frame.

$$G=2\gamma\omega_0\sum_m\frac{\chi_m r_{cm}^2}{r^3}\sin^2\theta_m\left(\begin{array}{l}\cos 3\phi\left(G_xD_{11}^{(m)}+G_yD_{12}^{(m)}+G_zD_{13}^{(m)}\right)+\\ \sin 3\phi\left(G_xD_{12}^{(m)}+G_yD_{22}^{(m)}+G_zD_{23}^{(m)}\right)\end{array}\right) \quad (25)$$

Where the elements $D_{jk}^{(m)}$ are of the diffusion tensor for the region outside the perturbers in Cartesian representation transformed into in the “cylinder PAS” of perturber *m*. The elements of pulsed field gradient **G** must be represented in the same frame.

$$H = 2\gamma\omega_0 \sum_m \frac{\chi_m \left(r_{cm}^2 - r_{Lm}^2\right)}{r^3} \sin^2\theta_m \begin{pmatrix} \cos 3\phi \left(G_x D_{11}^{(m)} + G_y D_{12}^{(m)} + G_z D_{13}^{(m)}\right) + \\ \sin 3\phi \left(G_x D_{12}^{(m)} + G_y D_{22}^{(m)} + G_z D_{23}^{(m)}\right) \end{pmatrix} \quad (26)$$

Where the elements $D_{jk}^{(m)}$ are of the diffusion tensor for the wall of perturber *m* in the cylinder PAS of perturber *m*, in Cartesian representation. The pulsed field gradient **G** should also be represented in that frame.

Simplified model

In the main text we use a simplified model in which diffusion outside the perturber region is isotropic, and in any perturber wall or lumen is axially symmetric with the unique axis parallel to the cylinder axis. The reduced expressions for the *b*-tensor alphabet, multiplied by the diffusion tensor, then become:

$$\mathbf{b}_A^{(m)} \cdot \mathbf{D}_{out}^{(m)} = \frac{\rho^2 t^3}{3} \frac{\chi_m^2 \omega_0^2 r_{cm}^4 \sin^4\theta_m}{r^6} D_{out} \quad (27)$$

where D_{out} is the isotropic diffusion coefficient of the space outside any perturbers.

$$\mathbf{b}_B^{(m)} \cdot \mathbf{D}_{out}^{(m)} = \frac{\rho^2 t^3}{3} \frac{\chi_m^2 \omega_0^2 \left(r_{cm}^2 - r_{Lm}^2\right)^2 \sin^4\theta_m}{r^6} D_{wall,R}^{(m)} \quad (28)$$

where $D_{wall,R}^{(m)}$ is the radial diffusivity in the wall of perturber *m*.

$$\mathbf{b}_C^{(m)} \cdot \mathbf{D}_{out}^{(m)} = \frac{\rho^2 t^3 \gamma^2}{3} D_{out} \left(G_x^2 + G_y^2 + G_z^2\right) \quad (29)$$

$$\mathbf{b}_E^{(m)} \cdot \mathbf{D}_{wall}^{(m)} = \frac{\rho^2 t^3 \gamma^2}{3} \left(D_{wall,R}^{(m)} \left(G_x^2 + G_y^2\right) + D_{wall,A}^{(m)} G_z^2\right) \quad (30)$$

Where $D_{wall,A}^{(m)}$ is the axial diffusivity of the wall of perturber *m*.

$$\mathbf{b}_F^{(m)} \cdot \mathbf{D}_{lumen}^{(m)} = \frac{\rho^2 t^3 \gamma^2}{3} \left(D_{lumen,R}^{(m)} \left(G_x^2 + G_y^2\right) + D_{lumen,A}^{(m)} G_z^2\right) \quad (31)$$

Where $D_{lumen,R}^{(m)}$ and $D_{lumen,A}^{(m)}$ are the radial and axial diffusivities in the lumen of perturber *m*.

$$\mathbf{b}_G^{(m)} \cdot \mathbf{D}_{out}^{(m)} = \frac{2\rho^2 t^3 \gamma\omega_0}{3} \frac{\chi_m r_{cm}^2 \sin^2\theta_m}{r^3} D_{out} \left(G_x \cos 3\phi + G_y \sin 3\phi\right) \quad (32)$$

$$\mathbf{b}_H^{(m)} \cdot \mathbf{D}_{lumen}^{(m)} = \frac{2\rho^2 t^3 \gamma\omega_0}{3} \frac{\chi_m \left(r_{cm}^2 - r_{Lm}^2\right)\sin^2\theta_m}{r^3} D_{lumen,R}^{(m)} \left(G_x \cos 3\phi + G_y \sin 3\phi\right) \quad (33)$$

Derivatives and coordinate systems

We must have a coordinate system and representation. Calculations are most easily performed in a coordinate system in which the z-axis of a given cylinder lies along the z-axis and B_0 lies in the xz plane. The most appropriate coordinate representation is cylindrical. The diffusion tensors and gradient operator must therefore be represented using the cylindrical system. The gradient operator in cylindrical coordinates is given by

$$\nabla = \begin{bmatrix} \frac{\partial}{\partial r} \\ \frac{1}{r}\frac{\partial}{\partial \phi} \\ \frac{\partial}{\partial z} \end{bmatrix} \quad (34)$$

The transformation between Cartesian and cylindrical coordinates is made through

$$D^{cyl} = \hat{Q}^T D^{Car} \hat{Q} \quad (35)$$

Where the transformation tensor is given by

$$\hat{Q} = \begin{pmatrix} \cos\phi & -\sin\phi & 0 \\ \sin\phi & \cos\phi & 0 \\ 0 & 0 & 1 \end{pmatrix} \quad (36)$$

This transformation is useful, as it is more convenient in simulations to enter diffusion tensors for each cylinder wall/lumen in Cartesian for in the principal axis system of that cylinder.

The necessary derivatives, using the cylindrical gradient operator, are given for cylindrical perturbers with walls of finite thickness by:

$$\nabla\Delta\omega_{out}^{(m)} = \begin{bmatrix} -\omega_0\chi_m \sin^2\theta_m \cos 2\phi \dfrac{r_{cm}^2}{r^3} \\ -\omega_0\chi_m \sin^2\theta_m \sin 2\phi \dfrac{r_{cm}^2}{r^3} \\ 0 \end{bmatrix} \tag{37}$$

$$\nabla\Delta\omega_{wall}^{(m)} = \begin{bmatrix} -\omega_0\chi_m \sin^2\theta_m \cos 2\phi \dfrac{r_{cm}^2 - r_{Lm}^2}{r^3} \\ -\omega_0\chi_m \sin^2\theta_m \sin 2\phi \dfrac{r_{cm}^2 - r_{Lm}^2}{r^3} \\ 0 \end{bmatrix} \tag{38}$$

$$\nabla\Delta\omega_{lumen}^{(m)} = \begin{bmatrix} 0 \\ 0 \\ 0 \end{bmatrix} \tag{39}$$

For the PFG terms:

$$\nabla\omega_D = -\gamma \begin{bmatrix} G_x \cos\phi + G_y \sin\phi \\ G_x \cos\phi - G_y \sin\phi \\ G_z z \end{bmatrix} \tag{40}$$

All these terms are given in the PAS of a particular perturber. So, for example, the expression for *A* requires that the diffusion tensor field outside the perturber region be transformed into the PAS of each cylinder before application of that expression.

Extended methods

Simulations

The tensor calculations of the theory section were performed with the aid of the Matlab Symbolic Math Toolbox and simulations of diffusion-mediated dephasing performed using Matlab 2015b. A set of classes were written for this purpose, which could calculate all the terms given in the theory section as well as determine effective diffusion tensors and T_2 from simulations including applied filed gradients at sufficient orientations, for any geometry of cylindrical perturbers.

To examine the combined effects of susceptibility differences and applied field gradients, we performed simulations using a geometry of a single walled cylinder with an outer radius of 1.5 µm, an inner radius of 0.7 µm in system of overall dimensions 6 x 6 x 6 $µm^3$ with a spatial resolution of 100 x 100 x 100 points. We were then able to perform simulations of diffusion-mediated decoherence with and without applied field gradients. Without field gradients, the anisotropy of T_2 could be determined by assigning an isotropic T_2 of 100 ms across the entire simulation region. With applied field gradients, an effective diffusion tensor could be fitted to the data as in any ordinary diffusion-weighted MRI dataset, using b-values of 0 and 1000 mm^{-2} s at a gradient amplitude of 0.04 T/m, with 6 non-collinear gradient directions. Outside the perturber region, an isotropic diffusion tensor $D_{xx}=D_{yy}=D_{zz}=0.8x10^{-3}$ mm^2 s^{-1} was assigned. In the lumen, an axially symmetric diffusion tensor with a z-axis parallel to the z-axis of the cylinder was defined with $D_{xx}=D_{yy}=0.3x10^{-3}$ mm^2 s^{-1}, $D_{zz}=1.5x10^{-3}$ mm^2 s^{-1}. In the wall, an anisotropic diffusion tensor with a z-axis parallel to the z-axis of the cylinder was defined with $D_{xx}=D_{yy}=0.6x10^{-3}$ mm^2 s^{-1}, $D_{zz}=1.3x10^{-3}$ mm^2 s^{-1}.

To examine the effects that crossing-fibre populations have on anisotropy of T_2 and diffusion parameters in the presence of field inhomogeneities, we created a perturber geometry of 32 walled cylinders. Either all 32 were parallel, or 16 were grouped and parallel in one direction, and the other 16 were parallel but grouped at an orientation 90° to the first group. The size of the system was 13 x 6.5 x 6.5 $µm^3$ with a spatial resolution of 200 x 100 x 100 points. The outer radius of each cylinder was 0.6 µm, the inner radius 0.35 µm. For T_2 anisotropy simulations, the time-domain contained 50 points between 0 and 1 second. For diffusion anisotropy simulations, B-values of 0 and 1000 mm^{-2} s were used, with 6 non-collinear gradient directions, and a gradient amplitude of 0.04 T/m. Diffusion parameters were as given in the single cylinder case.

Imaging parameters

In all, the MRI protocol contained the following:

3D T_1-weighted MPRAGE, sagittal, matrix size 256x256x208, resolution 0.86x0.86x0.86 mm^3, time 5:07 minutes, TI 900 ms, 2200 ms, α 9°, GRAPPA factor 2, 24 integrated reference lines.

T2 mapping was performed using a 2D multi-echo spin-echo sequence with, axial, with acquisition parameters: matrix size 162x192, 54 slices, resolution 1.15x1.15x1.98 mm^3 (including 10% slice gap), 9:50 minutes, TE 12 ms, 10 echoes acquired, TR 7000 ms, GRAPPA factor 2, 24 integrated reference lines, partial Fourier factor 7/8.

Diffusion imaging was performed using a 2D diffusion-weighted EPI pulse sequence with the parameters: axial, matrix size 128X128, 54 slices, resolution 1.88x1.88x1.98 mm^3 (including 10% slice gap), time 3:15 minutes, TE 87.4 ms, TR 2600 ms, GRAPPA factor 2, 24 integrated reference lines, partial Fourier factor 6/8, 60 bipolar diffusion-sensitising gradient directions, b-values 0 and 1000 s mm^{-2}, multi-band factor 3(5), repeated with anterior-to-posterior and posterior-to-anterior phase encoding (total time 6:30). Oblique orientations were not allowed, to simplify certain post-processing steps. Multi-band pulses used the time-shifted and phase-scrambled methods to reduce peak RF amplitude.

Fitting to experimental T2 anisotropy surfaces

In the main text, we define in Equations 13-15 a simple model for the effect of T_2 anisotropy, derived from our theory. This may be expressed in terms of the parameters $T_2^{\parallel} = 1/R_2^{iso}$ and *A*, representing the T_2 parallel to B_0 and amplitude of anisotropy respectively. We may also define the quantities $T_2^{\perp}$, representing T_2 perpendicular to B_0 and $T_2^{\Delta} = T_2^{\parallel} - T_2^{\perp}$, the "peak-to-trough" distance. In the main text we fit $T_2^{\parallel}$ and *A*. From these, the uncertainties in $T_2^{\perp}$ and T_2^{Δ} may be approximated from the relation

$$\Delta f = \sqrt{\sum_j \left(\frac{\partial f}{\partial x_j} \Delta x_j \right)^2} \tag{41}$$

Where *f* is a function of the set of parameters x_j with uncertainties Δx_j. Then we have

$$\Delta T_2^{\Delta} = \sqrt{\frac{T_2^{\parallel 4}}{\left(1 + A T_2^{\parallel}\right)^4} \Delta A^2 + \frac{A^2 \left(2 T_2^{\parallel} + A T_2^{\parallel 2}\right)^2}{\left(1 + A T_2^{\parallel}\right)^4} \Delta A T_2^{\parallel 2}} \tag{42}$$

$$\Delta T_2^{\perp} = \sqrt{\frac{\Delta T_2^{\parallel 2}}{\left(1+AT_2^{\parallel}\right)^4} + \frac{T_2^{\parallel 4}}{\left(1+AT_2^{\parallel}\right)^4}\Delta A^2} \tag{43}$$

Regression models

The full regression model, use to determine the size of the T_2 anisotropy effect and its interaction with other terms, was

$$\begin{aligned} R_2 \approx\ & AGE + AGE^2 + \sin^4\theta + FA + FA^2 + MD + MD^2 \\ & + AGE \times \sin^4\theta + AGE \times FA + AGE \times MD \\ & + \sin^4\theta \times FA + \sin^4\theta \times MD \\ & + FA \times MD + Const \end{aligned} \tag{44}$$

The reduced regression model, which did not include any T_2 anisotropy terms, was

$$\begin{aligned} R_2 \approx\ & AGE + AGE^2 + FA + FA^2 + MD + MD^2 \\ & + AGE \times FA + AGE \times MD \\ & + FA \times MD + Const \end{aligned} \tag{45}$$

These two regression models were fitted to the total sets of data pooled over all 40 participants, limited to the TBSS skeleton and furthermore restricted to particular tracts or tract groups of interest.

Regression coefficients

The following tables give the regression coefficients and statistics for the models inclusive of an effect of anisotropy used to describe the R_2 data in the WM skeleton. The regression coefficients and their uncertainties are given for the z-scores due to the different scales of the data.

Table SI1 provides regression coefficients and statistics for the full regression model in the CST, SLoF, UF and entire WM skeleton, and corresponds to the models plotted in Figure 4.

Table SI1 provides regression coefficients and statistics for the full regression model in early and late-myelinating white matter and corresponds to the models plotted in Figure 5.

Table SI1: Regression output for the "full regression model" including anisotropy in the 3 selected WM tracts and the entire WM skeleton. The abbreviation sin4(theta) refers to the sine of the angle between the principle direction of diffusion and B_0 raised to 4th power, FA is fractional anisotropy of diffusion, MD is mean diffusivity of diffusion. Coeff is the label for the term in the full regression model, Beta the regression coefficient value, SE its fitted standard error.

Region	Coeff	Beta	SE	t-stat	p-value
CST	Const	0.010456	2.94E-06	3558.4	0
	FA	-0.00036257	1.64E-06	-220.64	0
	MD	-9.75E-05	1.72E-06	-56.803	0
	AGE	-0.00017853	1.54E-06	-116.14	0
	sin4(theta)	0.00025475	1.64E-06	155.43	0
	FA^2	-2.86E-05	1.49E-06	-19.211	3.34E-82
	FA:MD	4.01E-05	1.79E-06	22.449	1.61E-111
	MD^2	5.03E-05	1.33E-06	37.921	5.8283e-314
	FA:AGE	1.05E-05	1.65E-06	6.4118	1.44E-10
	MD:AGE	-1.31E-05	1.65E-06	-7.9588	1.74E-15
	AGE^2	-9.09E-05	1.76E-06	-51.602	0
	FA:sin4(theta)	3.11E-05	1.71E-06	18.14	1.68E-73
	MD:sin4(theta)	5.12E-05	1.70E-06	30.107	7.31E-199
	AGE:sin4(theta)	1.81E-05	1.60E-06	11.303	1.29E-29
SLoF	Const	0.011716	2.23E-06	5246	0
	FA	-0.00017143	1.26E-06	-135.98	0
	MD	-0.00015507	1.29E-06	-120.61	0
	AGE	-0.00028864	1.21E-06	-239.05	0
	sin4(theta)	0.00021174	1.22E-06	173.48	0
	FA^2	2.27E-05	9.70E-07	23.376	8.65E-121
	FA:MD	2.59E-05	1.33E-06	19.394	9.31E-84
	MD^2	-4.59E-05	1.00E-06	-45.887	0
	FA:AGE	-8.08E-05	1.26E-06	-64.266	0
	MD:AGE	-4.02E-05	1.26E-06	-31.991	2.52E-224
	AGE^2	-0.00013392	1.38E-06	-96.772	0
	FA:sin4(theta)	5.88E-05	1.30E-06	45.196	0
	MD:sin4(theta)	5.89E-06	1.30E-06	4.5436	5.53E-06
	AGE:sin4(theta)	-1.53E-06	1.21E-06	-1.2672	0.2051

UF	(Intercept)	0.01258	2.99E-06	4203.7	0
	FA	0.00013268	1.65E-06	80.266	0
	MD	-0.00015593	1.66E-06	-94.184	0
	AGE	-0.00030996	1.59E-06	-195.21	0
	sin4(theta)	0.00029578	1.61E-06	183.32	0
	FA^2	-6.42E-05	1.33E-06	-48.249	0
	FA:MD	5.18E-05	1.79E-06	28.833	2.11E-182
	MD^2	2.60E-06	1.42E-06	1.8318	0.066982
	FA:AGE	-3.23E-05	1.64E-06	-19.693	3.07E-86
	MD:AGE	-2.76E-05	1.68E-06	-16.464	7.32E-61
	AGE^2	-0.00014606	1.83E-06	-79.878	0
	FA:sin4(theta)	7.37E-05	1.72E-06	42.819	0
	MD:sin4(theta)	2.79E-05	1.72E-06	16.259	2.12E-59
	AGE:sin4(theta)	-4.47E-06	1.59E-06	-2.8152	0.0048754
All	Const	0.011899	9.43E-07	12625	0
	FA	-0.00022303	5.04E-07	-442.91	0
	MD	-0.00022945	5.15E-07	-445.38	0
	AGE	-0.00026989	4.86E-07	-554.82	0
	sin4(theta)	0.00035446	5.01E-07	706.95	0
	FA^2	-4.25E-05	4.35E-07	-97.815	0
	FA:MD	-3.39E-05	5.18E-07	-65.487	0
	MD^2	-3.34E-05	4.30E-07	-77.548	0
	FA:AGE	-5.45E-05	4.97E-07	-109.72	0
	MD:AGE	-2.88E-05	5.07E-07	-56.786	0
	AGE^2	-0.00012751	5.58E-07	-228.51	0
	FA:sin4(theta)	0.00018281	5.19E-07	352.05	0
	MD:sin4(theta)	2.05E-05	5.29E-07	38.815	0
	AGE:sin4(theta)	-9.87E-06	4.93E-07	-20.023	3.52E-89

Table SI2: Regression output for the full model including anisotropy in the WM skeleton classified into early-myelinating and late-myelinating voxels. The abbreviation sin4(theta) refers to the cosine of the angle between the principle direction of diffusion and B_0 raised to 4th power, FA is fractional anisotropy of diffusion, MD is mean diffusivity of diffusion. Coeff is the label for the term in the full regression model, Beta the regression coefficient value, SE its fitted standard error.

Region	Coeff	Beta	SE	t-stat	p-value
Early	Const	0.011354	2.05E-06	5540.2	0
	FA	-0.00043299	1.08E-06	-401.84	0
	MD	-0.00023383	1.12E-06	-208.86	0
	AGE	-0.00021561	1.04E-06	-208.28	0
	sin4(theta)	0.00051635	1.09E-06	473.81	0
	FA^2	1.53E-05	9.97E-07	15.325	5.32E-53
	FA:MD	-3.24E-05	1.14E-06	-28.429	1.06E-177
	MD^2	-3.21E-05	9.68E-07	-33.147	8.93E-241
	FA:AGE	-5.27E-06	1.06E-06	-4.9796	6.37E-07
	MD:AGE	-4.34E-05	1.10E-06	-39.447	0
	AGE^2	-0.00010866	1.19E-06	-91.602	0
	FA:sin4(theta)	0.00017927	1.11E-06	160.9	0

	MD:sin4(theta)	-5.01E-05	1.15E-06	-43.568	0
	AGE:sin4(theta)	-8.18E-06	1.08E-06	-7.5875	3.26E-14
Late	Const	0.012067	1.20E-06	10076	0
	FA	-9.56E-05	6.44E-07	-148.38	0
	MD	-0.00024269	6.51E-07	-372.78	0
	AGE	-0.00029601	6.28E-07	-471.08	0
	sin4(theta)	0.00023767	6.42E-07	370.41	0
	FA^2	1.77E-05	5.26E-07	33.565	6.47E-247
	FA:MD	1.15E-05	6.65E-07	17.247	1.18E-66
	MD^2	-1.97E-05	5.36E-07	-36.703	8.34E-295
	FA:AGE	-5.23E-05	6.41E-07	-81.508	0
	MD:AGE	-2.86E-05	6.50E-07	-43.987	0
	AGE^2	-0.00013155	7.22E-07	-182.29	0
	FA:sin4(theta)	8.25E-05	6.70E-07	123.06	0
	MD:sin4(theta)	4.33E-05	6.74E-07	64.238	0
	AGE:sin4(theta)	-1.20E-05	6.32E-07	-18.977	2.67E-80

Crossing fibres at different glancing angles

In the main text, we show how the T_2 and FA depend on the polar and azimuthal angles between a system of model axons and an applied magnetic field when there are mesoscopic magnetic field inhomogeneities arising due to the different magnetic susceptibility of the myelin sheath. However, the glancing angle between bundles of axons was either 0 or 90°. In Figure SI1, we include simulations at more fibre bundle glancing angles. The simulation time for Figure SI1, which includes 91 sets of polar and azimuthal angles relative to B_0 and 4 fibre glancing angles, was 14 hours on a PC with 16 GB RAM and a 4-core 3.1 GHz CPU, using Matlab 2015b.

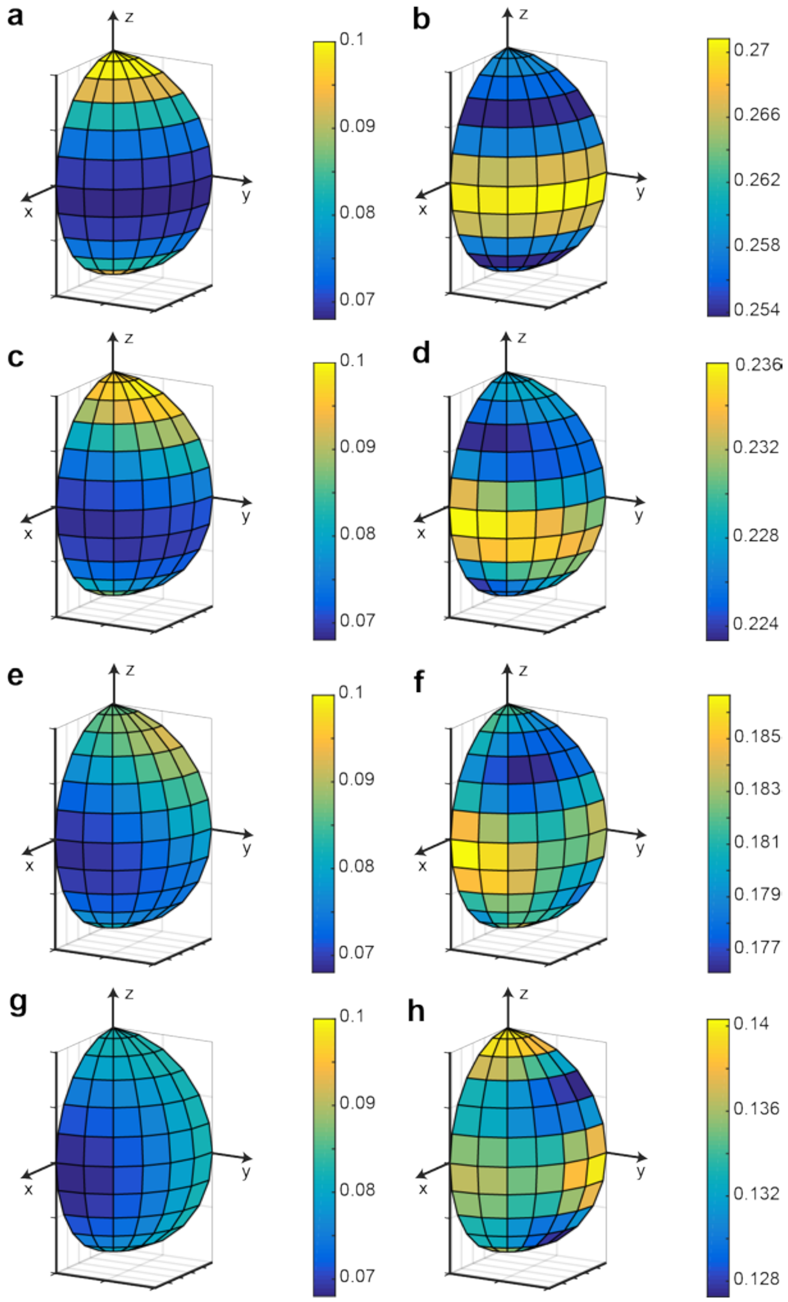

Figure SI1: T_2 and FA angular dependence relative to B_0 (parallel to z) for crossing fibre bundles with the bundles at different glancing angles. Panels a,c,e,g show the T_2, panels b,d,f,h show the FA of diffusion. The fibre glancing angles are (a,b) 0°, (c,d) 30°, (e,f) 60°, (g,h) 90°. Simulation parameters may be found in the main text. Note that the T_2 panels are all on the same colour scale, the FA panels are not due to the broader range of values.

References

1. Torrey HC (1956) Bloch Equations with Diffusion Terms. *Phys Rev* 104(3):563-565.
2. Stejskal EO & Tanner JE (1965) Spin Diffusion Measurements: Spin Echoes in the Presence of a Time-Dependent Field Gradient. *J. Chem. Phys.* 42(1):288-292.

3. Basser PJ, Mattiello J, & LeBihan D (1994) MR diffusion tensor spectroscopy and imaging. *Biophys. J.* 66(1):259-267.
4. Knight MJ & Kauppinen RA (2016) Diffusion-mediated nuclear spin phase decoherence in cylindrically porous materials. *J. Magn. Reson.* 269:1-12.
5. Feinberg DA*, et al.* (2010) Multiplexed echo planar imaging for sub-second whole brain FMRI and fast diffusion imaging. *PLoS One* 5(12):e15710.
