# Supplementary material for: Magnetic Resonance Relaxation Anisotropy: Physical Principles and Uses in Microstructure Imaging
Source: Biophys J. 2017 Apr 11;112(7):1517–28. doi: 10.1016/j.bpj.2017.02.026 (PMC5390049; doi:10.1016/j.bpj.2017.02.026)
Supplement: Document S2. Article plus Supporting Material [file mmc2.pdf]

Biophysical Journal
Article

Biophysical Society

Magnetic Resonance Relaxation Anisotropy: Physical Principles and Uses in Microstructure Imaging

Michael J. Knight,[1,*] Serena Dillon,[2] Lina Jarutyte,[1] and Risto A. Kauppinen[1,3]
[1]School of Experimental Psychology, [2]ReMemBr group, Institute for Clinical Neurosciences, and [3]Clinical Research and Imaging Centre, University of Bristol, Bristol, United Kingdom

ABSTRACT Magnetic resonance imaging (MRI) provides an excellent means of studying tissue microstructure noninvasively since the microscopic tissue environment is imprinted on the MRI signal even at macroscopic voxel level. Mesoscopic variations in magnetic field, created by microstructure, influence the transverse relaxation time (T_2) in an orientation-dependent fashion (T_2 is anisotropic). However, predicting the effects of microstructure upon MRI observables is challenging and requires theoretical insight. We provide a formalism for calculating the effects upon T_2 of tissue microstructure, using a model of cylindrical magnetic field perturbers. In a cohort of clinically healthy adults, we show that the angular information in spin-echo T_2 is consistent with this model. We show that T_2 in brain white matter of nondemented volunteers follows a U-shaped trajectory with age, passing its minimum at an age of ~30 but that this depends on the particular white matter tract. The anisotropy of T_2 also interacts with age and declines with increasing age. Late-myelinating white matter is more susceptible to age-related change than early-myelinating white matter, consistent with the retrogenesis hypothesis. T_2 mapping may therefore be incorporated into microstructural imaging.

INTRODUCTION

The power of magnetic resonance imaging (MRI) is in its capability to deliver information on microstructure, meaning that one may manipulate the signal and imprint the signature of microscopic structures upon it. By such means, the existence and nature of objects very much smaller than a voxel may be inferred. This is nonetheless an evolving technology and advances in hardware, software, and theoretical understanding continue to extend its utility.

Understanding the microstructural changes taking place in the human brain with age is of great importance in identifying and treating diseases associated with aging, such as various classes of dementia and stroke. The dementia challenge is a particularly large one because we are currently limited to making diagnoses only once clinical presentation is severe. However, the identification of pathology before potentially irreversible loss of tissue must identify the chemical or microstructural causes in treatable tissue. Here, the availability of methods sensitive to widespread but subtle changes is particularly important. Our objective in this article is to develop and apply the phenomenon of transverse relaxation time (T_2) anisotropy to reveal details of human white matter (WM).

Microstructure influences the range of resonance frequencies that a diffusing nuclear spin may sample, therefore influencing the coherence and decoherence of spin phase (thus signal amplitude) as well as total accumulated signal phase. The signature of microstructure is thereby imprinted on both MRI signal amplitude and phase. Several modalities exploit these two distinct phenomena, and in different ways. In diffusion imaging, external magnetic field gradients are applied. Coherence is lost more rapidly if an applied field gradient is parallel to a direction in which there is less restriction to translational diffusion on a micrometer scale such that a broad range of resonance frequencies is sampled (1). Therefore, one may infer the existence and nature of structures of micrometer size (2). In the absence of applied field gradients, microstructure nevertheless creates an inhomogeneous local magnetic field on a mesoscopic scale (3–7), due to differences in magnetic susceptibility within the microstructural components in the system. This influences the signal in gradient-echo and spin-echo imaging, as spins in a voxel sample a range of resonance frequencies—similarly to the

Submitted December 12, 2016, and accepted for publication February 21, 2017.

*Correspondence: mk13005@bristol.ac.uk

Editor: Francesca Marassi

http://dx.doi.org/10.1016/j.bpj.2017.02.026

© 2017 Biophysical Society.

This is an open access article under the CC BY license (http://creativecommons.org/licenses/by/4.0/).

CrossMark

application of a field gradient, but with the inhomogeneity arising from the system under study, rather than externally applied. In gradient-echo MRI, the signal accumulates phase, which has given rise to quantitative susceptibility mapping (QSM) (8,9) and susceptibility tensor imaging (STI) (10). In spin-echo MRI, translational diffusion through the inhomogeneous field labels the T_2 with the signature of the microstruturally induced local magnetic field (11). We have recently demonstrated this to be so in human WM, in which the spin-echo T_2 of human WM shows a pattern of anisotropy by which its maximum occurs when an ordered system is parallel to the applied field B_0 and minimized when perpendicular (12). We have also provided a theoretical framework by which it may be explained (13)—opening the door to applications of T_2 anisotropy. However, relating microstructure to the perturbations to magnetic fields resulting from it, and thus to measurements of coherence lifetimes and diffusion-mediated dephasing, remains challenging. The link between microstructure and its influence on most MRI-observable quantities remains a challenging one to make especially in systems such as the brain.

In this article, we develop the principles of spin-echo T_2 anisotropy and apply it to reveal details of human WM aging. By doing so, we reveal that, in a cohort of healthy persons, T_2 anisotropy is sensitive not just to the particular WM tract, but the regional age effects. In particular, late-myelinating WM has markedly lower anisotropy and loses its anisotropy more rapidly in later life than early-myelinating WM. These findings are consistent with the retrogenesis theory (14). As such, we seek to establish relaxation anisotropy as a tool in the arsenal of microstructural imaging modalities.

Theory section: the *b*-tensor field and coherence lifetime anisotropy

In a spin-echo experiment, where phase terms are entirely refocused, if there exists a resonance frequency inhomogeneity $\omega_I(\mathbf{x})$, due to mesoscopic susceptibility differences, applied field gradients or other sources, the signal amplitude evolves according to (13)

$$S(t) = \left| \int A_0(\mathbf{x})\exp(-\mathbf{b}(\mathbf{x})\cdot\mathbf{D}(\mathbf{x}))\exp\left(-R_2^{iso}(\mathbf{x})t\right)d\mathbf{x} \right|, \quad (1)$$

where A_0 is the signal amplitude at (time) $t = 0$, $\mathbf{D}(\mathbf{x})$ is the translational diffusion tensor field, $R_2^{iso}(\mathbf{x})$ is the (isotropic) transverse relaxation rate coefficient scalar field, and we have introduced $\mathbf{b}(\mathbf{x})$ as the *b*-tensor field. The elements of the *b*-tensor field are defined as

$$b_{jk}(\mathbf{x}) = \frac{\rho^2 t^3}{3}\frac{\partial\omega_I(\mathbf{x})}{\partial x_j}\frac{\partial\omega_I(\mathbf{x})}{\partial x_k}, \quad (2)$$

where ρ is the coherence order (15). This is analogous to the theory common in diffusion imaging and replaces the *b*-value, to which it reduces if the frequency inhomogeneity $\omega_I(\mathbf{x})$ is linear (such as due solely to applied field gradients). We have also defined, in admittedly flexible notation:

$$\mathbf{b}(\mathbf{x})\cdot\mathbf{D}(\mathbf{x}) = \sum_{jk} b_{jk}(\mathbf{x})D_{jk}(\mathbf{x}). \quad (3)$$

The fact that we have a *b*-tensor field implies that, provided $\omega_I(\mathbf{x})$ exists, diffusion-mediated decoherence, and therefore T_2, is anisotropic. That is, the magnetic field created by tissue microstructure in response to the applied field transforms with orientation relative to the applied field, and the form of the spin phase decoherence transforms with it.

The frequency inhomogeneity function, $\omega_I(\mathbf{x})$, may be decomposed into a sum of terms representing the response of the system under observation to the applied field, and any deliberate inhomogeneity due to the use of applied field gradients:

$$\begin{aligned}\omega_I(\mathbf{x}) &= \Delta\omega(\mathbf{x}) + \gamma\mathbf{G}\cdot\mathbf{x} \\ &= \Delta\omega(\mathbf{x}) + \omega_D(\mathbf{x})\end{aligned}, \quad (4)$$

where $\Delta\omega(\mathbf{x})$ is the frequency difference from the Larmor frequency arising due to magnetic susceptibility differences within the system, $\mathbf{G}$ is a field gradient, and $\omega_D(\mathbf{x})$ is the linear frequency shift arising due to applied (typically pulsed) field gradients. There is the following interaction between the effects of the frequency difference function and applied field gradients:

$$\begin{aligned}\mathbf{b}\cdot\mathbf{D} &= \frac{\rho^2 t^3}{3}\sum_{j,k}\frac{\partial(\Delta\omega+\omega_D)}{\partial x_j}\frac{\partial(\Delta\omega+\omega_D)}{\partial x_k}D_{jk} \\ &= \frac{\rho^2 t^3}{3}\left[\begin{array}{l}[\nabla\Delta\omega]^T\cdot\mathbf{D}\nabla\Delta\omega \\ +[\nabla\omega_D]^T\cdot\mathbf{D}\nabla\omega_D \\ +\left([\nabla\Delta\omega]^T\cdot\mathbf{D}\nabla\omega_D + [\nabla\omega_D]^T\cdot\mathbf{D}\nabla\Delta\omega\right)\end{array}\right].\end{aligned} \quad (5)$$

On the second line of this equation, from top to bottom, the three terms represent dephasing due to the system's response to the applied field only, dephasing due to the applied field gradients only, and dephasing due to the interaction between those effects. The superscript T represents the transpose operation. Additional details on this expansion are provided in the Supporting Material.

Walled cylinder model

For the exploration of the effects of susceptibility differences, a model is useful. With a view to understanding the effects of myelinated axons upon diffusion-mediated decoherence in MRI of the human brain, we use a model of cylindrical field perturbers whose walls contain a material with a different magnetic susceptibility from their surroundings. The system-induced frequency difference $\Delta\omega(\mathbf{x})$ may be

calculated for any geometry of a set of cylindrical field perturbers (5) as

$$\Delta\omega(\mathbf{x}) = \sum_m \begin{cases} \frac{\omega_0\chi_m}{2}\sin^2\theta_m\cos 2\phi\left(\frac{r_{cm}^2}{r^2}\right), & r \geq r_{cm} \\ \frac{\omega_0\chi_m}{2}\left(\cos^2\theta_m - \frac{1}{3} - \sin^2\theta_m\cos 2\phi\left(\frac{r_{cm}^2 - r_{Lm}^2}{r^2}\right)\right), & r_{Lm} \leq r < r_{cm} \\ 0, & r < r_{Lm} \end{cases}, \tag{6}$$

where ω_0 is the Larmor frequency, θ_m is the polar angle between the long axis of the cylinder m and B_0, and the coordinates ϕ, r represent position in a cylindrical system with the z-axis parallel to the cylinder long axis and B_0 defined in the xz plane. χ_m is the susceptibility difference (with the susceptibility tensor assumed isotropic) between the wall of the cylinder and outside, r_{cm} is the cylinder outer radius, and r_{Lm} the lumen radius of cylinder m. The summation is taken over all cylinders, each being indexed by m.

The diffusion tensor field is treated such that each cylinder lumen has its own diffusion tensor, each cylinder wall has its own diffusion tensor and the surroundings have a unique diffusion tensor. The b-tensor field may be represented as a sum over perturbers for each region as

$$\begin{aligned}\mathbf{b} &= \sum_m \mathbf{b}^{(m)} \\ &= \mathbf{b}_A + \mathbf{b}_B + \mathbf{b}_C + \mathbf{b}_E + \mathbf{b}_F + \mathbf{b}_G.\end{aligned} \tag{7}$$

In this "alphabet" of terms, we recognize A and B as the dephasing due to the system's response to the applied field only (due to susceptibility differences), C, E, and F as the dephasing due to the applied field gradients, and G and H due to the interaction between those two phenomena. To simplify proceedings, we impose the condition that the diffusion tensor outside the perturbers is isotropic, and that the diffusion tensor for each perturber wall and lumen is axially symmetric with its unique axis parallel to that particular perturber's axis. Then we obtain

$$\mathbf{b}_A^{(m)} \cdot \mathbf{D}_{out}^{(m)} = \frac{\rho^2 t^3}{3}\frac{\chi_m^2\omega_0^2 r_{cm}^4 \sin^4\theta_m}{r^6} D_{out}, \tag{8}$$

where D_{out} is the isotropic diffusion coefficient of the space outside any perturbers. We also obtain

$$\mathbf{b}_B^{(m)} \cdot \mathbf{D}_{out}^{(m)} = \frac{\rho^2 t^3}{3}\frac{\chi_m^2\omega_0^2\left(r_{cm}^2 - r_{Lm}^2\right)^2\sin^4\theta_m}{r^6} D_{wall,R}^{(m)}, \tag{9}$$

where $D_{wall,R}^{(m)}$ is the radial diffusivity in the wall of perturber m, and

$$\begin{aligned}\mathbf{b}_G^{(m)} \cdot \mathbf{D}_{out}^{(m)} &= \frac{2\rho^2 t^3\gamma\omega_0}{3}\frac{\chi_m r_{cm}^2\sin^2\theta_m}{r^3} \\ &\quad \times D_{out}\left(G_x\cos 3\phi + G_y\sin 3\phi\right),\end{aligned} \tag{10}$$

$$\begin{aligned}\mathbf{b}_H^{(m)} \cdot \mathbf{D}_{lumen}^{(m)} &= \frac{2\rho^2 t^3\gamma\omega_0}{3}\frac{\chi_m\left(r_{cm}^2 - r_{Lm}^2\right)\sin^2\theta_m}{r^3} \\ &\quad \times D_{lumen,R}^{(m)}\left(G_x\cos 3\phi + G_y\sin 3\phi\right).\end{aligned} \tag{11}$$

The C, E, and F terms are the same as in conventional treatments (applied field gradients only) and may be found in the Supporting Material, as well as more general expressions.

This theory predicts a $\sin^4\theta$ dependence for diffusion-mediated decoherence due to susceptibility differences and a $\sin^2\theta$ dependence for the interaction between susceptibility differences and applied field gradients. For a voxel in which perturbers share a common axis of alignment (such as through which a single WM fiber tract passes), and in the absence of "significant" effects of applied field gradients, we can therefore anticipate an anisotropy of spin-echo R_2, scaled simply by $\sin^4\theta$, with θ the common angle between fiber and B_0 as

$$S(t) \approx \int A_0 \exp\left(-\alpha t^3\sin^4\theta\right)\exp\left(-R_2^{iso}t\right)d\mathbf{x}. \tag{12}$$

Therefore we arrive at the following simple "semi-heuristic" expression for spin-echo R_2:

$$R_2 = R_2^{iso} + A\sin^4\theta, \tag{13}$$

where the "amplitude of anisotropy" A depends on the set of echo times at which the signal is sampled (due to the cubic time dependence of signal decay). A is also scaled by the square of susceptibility differences between cylinder walls and surroundings, and the square of Larmor frequency (therefore applied field). $\sin^4\theta$ may be approximated by calculating the angle between the principal eigenvector of the diffusion tensor and the applied magnetic field. We can equivalently express this in terms of T_2 as

$$T_2 = \frac{T_2^{\parallel}}{1 + AT_2^{\parallel}\sin^4\theta}, \tag{14}$$

where $T_2^{\parallel} = 1/R_2^{iso}$ is the T_2 parallel to B_0. When $\theta = 90°$ we obtain the definition of the quantity $T_2^{\perp}$, from which we can define the "peak-to-trough" distance in T_2 between parallel and perpendicular orientations as

$$\begin{aligned} T_2^{\Delta} &= T_2^{\parallel} - T_2^{\perp} \\ &= \frac{AT_2^{\parallel 2}}{1 + AT_2^{\parallel}} \end{aligned}. \tag{15}$$

MATERIALS AND METHODS

Simulations

A set of classes were written to perform simulations of diffusion-mediated decoherence using MATLAB 2015b (The MathWorks, Natick, MA). To examine the combined effects of susceptibility differences and applied field gradients, we performed simulations using a geometry of a single walled cylinder. To examine the effects that crossing-fiber populations have on anisotropy of T_2 and diffusion parameters in the presence of field inhomogeneities, we created a perturber geometry of 32 walled cylinders. Either all 32 were parallel, or 16 were grouped and parallel in one direction, and the other 16 were parallel but grouped at an orientation 90° to the first group. In all cases, simulations were performed without applied field gradients (to determine T_2 anisotropy) and with six noncollinear gradients to explore the effect on diffusion tensor parameters. Complete simulation parameters are available in the Supporting Material.

Experimental MRI

A total of 40 participants were recruited for this study (25 females, aged 23–71). They were required to have no known neurological disorder, past or present. All participants gave informed consent, and ethical approval was granted by the University of Bristol Faculty of Science Research Ethics Committee. All data were acquired using a Siemens Magnetom Skyra 3T system (Siemens Healthcare, Erlangen, Germany) equipped with a 32-channel head coil 2-channel parallel transmit body coil. The acquisition included a three-dimensional (3D) T_1-weighted MPRAGE (sagittal, 0.86 × 0.86 × 0.86 mm^3), two-dimensional (2D) multiecho spin-echo (axial, 1.15 × 1.15 × 1.98 mm^3) and 2D multiband diffusion tensor imaging (DTI) (16) (axial, 1.88 × 1.88 × 1.98 mm^3). Complete acquisition parameters are listed in the Supporting Material.

T_2 maps were computed by a voxel-wise fit of a monoexponential function in a logarithmic space, excluding the first echo. This was done since the pulse sequence allows the passage of both spin and stimulated echoes due to the use of identical crusher gradients astride each refocusing pulse, though the first echo contains only spin echo contributions.

Diffusion tensor images were computed using FMRIB Software Library (FSL). Distortions caused by eddy currents were minimized using the program eddy (17), and gross distortions due to interfaces between materials with different magnetic susceptibility corrected with the program topup (18), before fitting diffusion tensors with dtifit. A single effective diffusion tensor was assumed for each voxel.

For the determination of age-dependent effects in the major WM tracts, the tract-based spatial statistics (TBSS) framework was used to identify a WM skeleton, implemented in FSL (19,20). Fractional anisotropy (FA) images were registered to the FMRIB58_FA standard template and the FA skeleton determined at a threshold of 0.2 after which the radial diffusivity (RD), mean diffusivity (MD), axial diffusivity (AxD), and T_2 maps were also skeletonized using the tbss_non_FA command.

Analysis of T_2 anisotropy

Anisotropy of T_2 was examined by two approaches. First, we used the method we have previously published to provide a heuristic demonstration as a surface plot of T_2 as a function of FA and the angle θ (between the principal direction of diffusion and B_0). In this method, FA and θ are bin-ranged to create 2D bins. All T_2 observations falling into a bin are averaged. A surface plot may be thereby produced. Data are required in a common space, chosen for each participant as that of their DTI data, resampled to 1 mm isotropic resolution.

In the second approach, we created a regression model that could be fitted to the data, motivated by the theory presented in this article and our recent work. It was more practical to work with R_2 than T_2, as R_2 terms are effectively additive and linear in the anisotropy effect. A "full" model was constructed, modeling the effects of FA, MD, and age upon R_2 up to second-order polynomials and anisotropy to first-order ones (the latter as per the theory section). All interaction terms were retained. A "reduced" model was also used, which did not include any T_2 anisotropy terms, and compared with the full model. The full model was also used to examine differences between early-myelinating and late-myelinating WM fiber tracts. Tracts of the Johns Hopkins University (JHU) WM atlas (21,22) were classified simply as "late-myelinating" or "early-myelinating" according to whether they have detectable levels of myelination at birth (23). Each voxel of the TBSS skeleton was therefore given such a label according to the most probable tract as identified by the JHU atlas, and the regression model applied separately for the two groups of data. Before fitting, data were demeaned and normalized by standard deviation (converted to z-scores), as the variables are on different scales. The regression analyses used the LinearModel class of MATLAB 2015b. Full expressions are given in the Supporting Material.

RESULTS

Interaction between system interactions and applied field gradients

The results of simulations for a single "thick-walled" cylinder are shown in Fig. 1. In Fig. 1, *a–c*, the T_2 as a function of orientation are shown. The T_2 expresses the anticipated orientation dependence, with its minimum when the cylinder is perpendicular to B_0, for at such an orientation the magnetic field is rendered most inhomogeneous if the wall has a different susceptibility from the surroundings. Accordingly, the broadest distribution of resonance frequencies is sampled by each spin, and so decoherence most severe. The observable FA for the system is reduced perpendicular to B_0 (Fig. 1 *b*), while MD is increased (Fig. 1 *c*). This is because dephasing in the vicinity of the wall is increased when perpendicular to B_0, giving the impression of increased diffusivity in all directions. This, of course, increases observable MD, but also decreases normalized differences between eigenvalues of the diffusion tensor, and therefore decreases the observable FA. The interaction terms between the applied field gradients and diffusion-mediated decoherence due to susceptibility differences have only a small influence, slightly reducing the overall rate of decoherence. By inspection of Fig. 1 *e*, such terms may be positive or negative, so once averaged over the domain of simulation, the effects are (somewhat) suppressed.

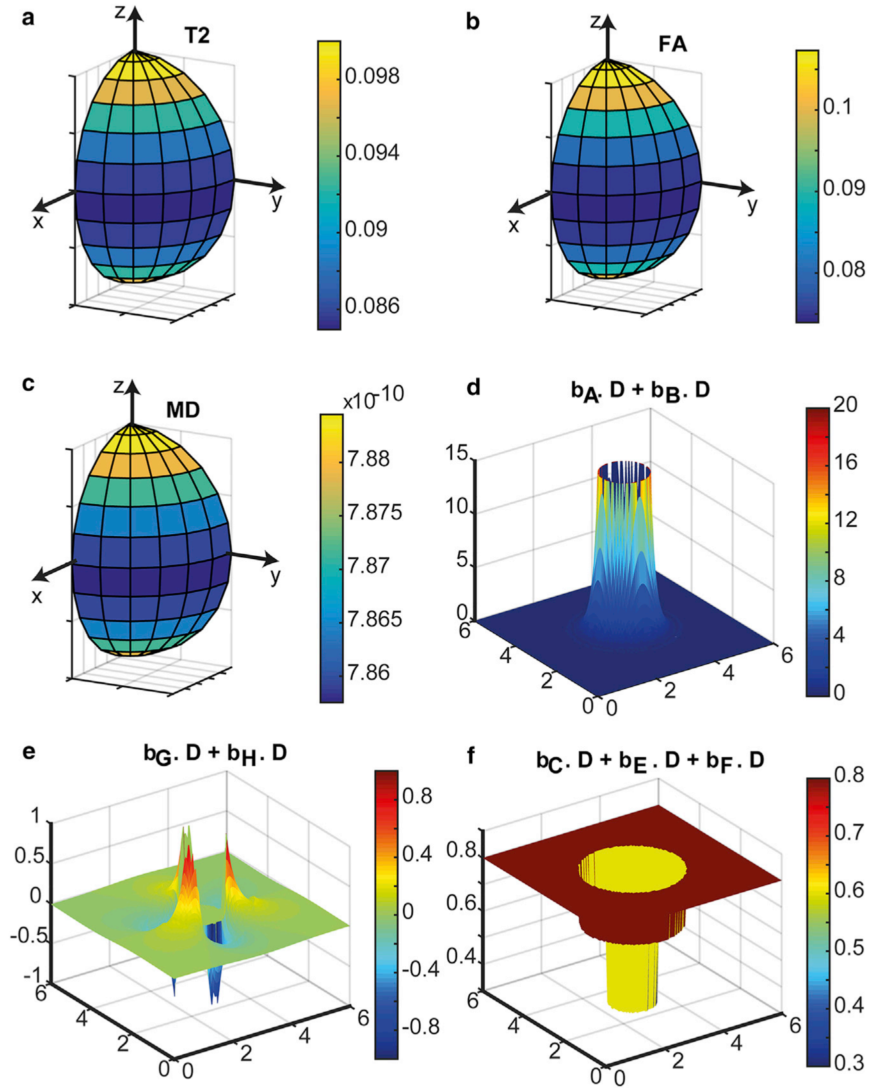

FIGURE 1 The effects of diffusion-mediated dephasing in the presence of susceptibility differences and applied field gradients for a single cylinder parallel to the z axis. (*a*)–(*c*) show the T_2 (scale bar units s), FA (scale bar unitless), and MD (scale bar $m^2 s^{-1}$) respectively simulated for various polar and azimuthal angles relative to B_0. (*d*)–(*f*) show the products of the *b*-tensor field and the diffusion tensor field for the three categories of dephasing. (*d*) shows the effects of susceptibility differences only, (*e*) shows the interaction between susceptibility differences and an applied field gradient parallel to the x axis, and (*f*) shows the effects of the applied field gradient parallel to x only. Note that different scales are used in each panel. To see this figure in color, go online.

The effects of crossing fibers on T_2 anisotropy

We compare the results of simulating diffusion-mediated decoherence for a single-fiber population and crossing-fiber system in Fig. 2. For the single-fiber population, the anisotropy of T_2 follows the familiar pattern of depending only on the angle between the longitudinal axis of the perturbers and B_0 (Fig. 1 *c*). For the crossing-fiber system, both the polar and azimuthal angle between the system of perturbers and B_0 contribute to the *b*-tensor field. The T_2 is minimized when both sets of perturbers are perpendicular to B_0, which occurs at $\theta = 90^\circ$, $\phi = 0^\circ$. T_2 is maximized when either population is parallel to B_0, but the other is then perpendicular so T_2 remains lower than the single-fiber system. The maximum T_2 in the crossing-fiber system is therefore lower than in the single-fiber system.

Examining the FA in the single-fiber case, it is maximized when the perturbers are perpendicular to B_0 (Fig. 2 *c*), though its minimum is not at the parallel orientation. Examining MD (Fig. 2 *e*), it follows a similar anisotropy to T_2 (in the noncrossing case). This is for the same reasons as in the single-cylinder case. The main reason for which FA is reduced in the crossing fiber relative to the single-fiber case is of course the lack of a unique axis of order. The anisotropy of FA in this case is rather complicated, but again we see FA maximized (raised above the "true" value) if one or the other set of perturbers is perpendicular to B_0 (Fig. 2 *d*). The MD anisotropy (Fig. 2 *f*) is very similar to the T_2 anisotropy, the MD being reduced from its true value when the contribution to dephasing from all contributions to the *b*-tensor field is maximized. This is at $\theta = 90^\circ$, $\phi = 0^\circ$. In the Supporting Material, we provide similar plots to Fig. 2, for fiber crossing angles other than 0° and 90°.

Experimental demonstration of T_2 anisotropy

In Fig. 3, the results of a heuristic approach to extracting the effect of T_2 anisotropy are shown, along with the fit of

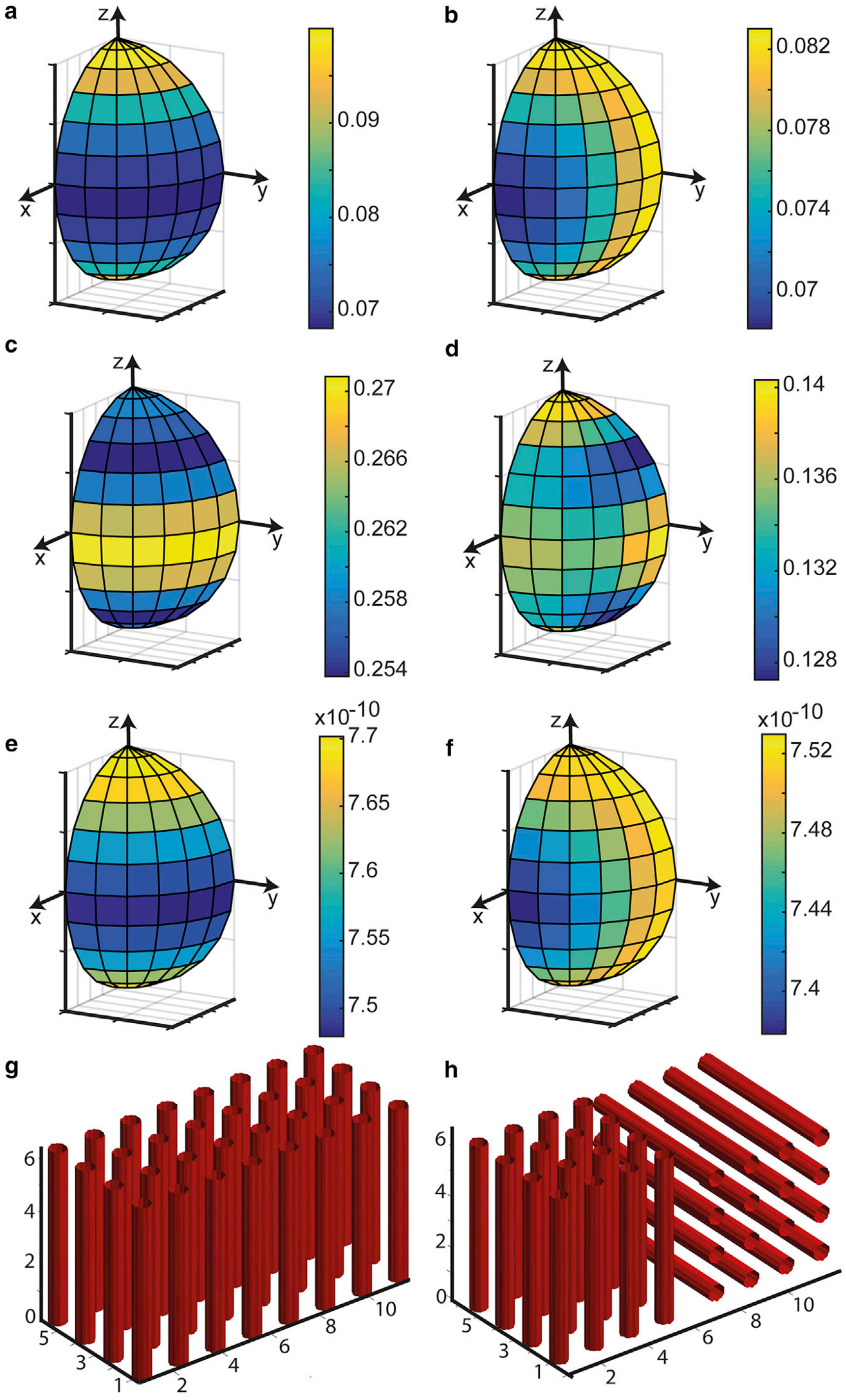

FIGURE 2 Simulations of diffusion-mediated decoherence, and its effects on T_2 and diffusion tensor parameters for a single-fiber population and crossing fibers at 90°. (*a*), (*c*), and (*e*) show simulations in the single-fiber case; (*b*), (*d*), and (*f*) show simulations in the crossing-fiber case. (*a*) and (*b*) show T_2 on a sphere (scale bar units: s), (*c*) and (*d*) show FA on spheres (scale bar unitless), (*e*) and (*f*) show MD on spheres (scale bar units: m^2 s^{-1}), and (*g*) and (*h*) show the perturber geometry. In (*g*) and (*h*), coordinates are given in units of micrometer. To see this figure in color, go online.

Eq. 13. In this analysis, participants were grouped into four age ranges with 10 participants in each subgroup. Therefore, any variance in the data because of factors such as age range and MD is absorbed by averaging over many observations at each selected 2D bin of θ and FA. There is a clear effect of anisotropy, consistent with our previous findings, with high FA (a high degree of order) corresponding to a high degree of T_2 anisotropy. We can also see that the entire surface plot shifts up with age (T_2 increases generally with age in WM), though the "peak-to-trough" (effect of anisotropy) decreases with increasing age, implying an increase in the isotropic T_2 with age. The peak-to-trough distance is quantifiable, giving a convenient parameterization of the overall "effect of anisotropy." This quantity, T_2^{Δ}, is plotted in Fig. 1 *e*. In the youngest age group of 23.1–32.6 years, for the FA bin range 0.379–0.421, it has the value

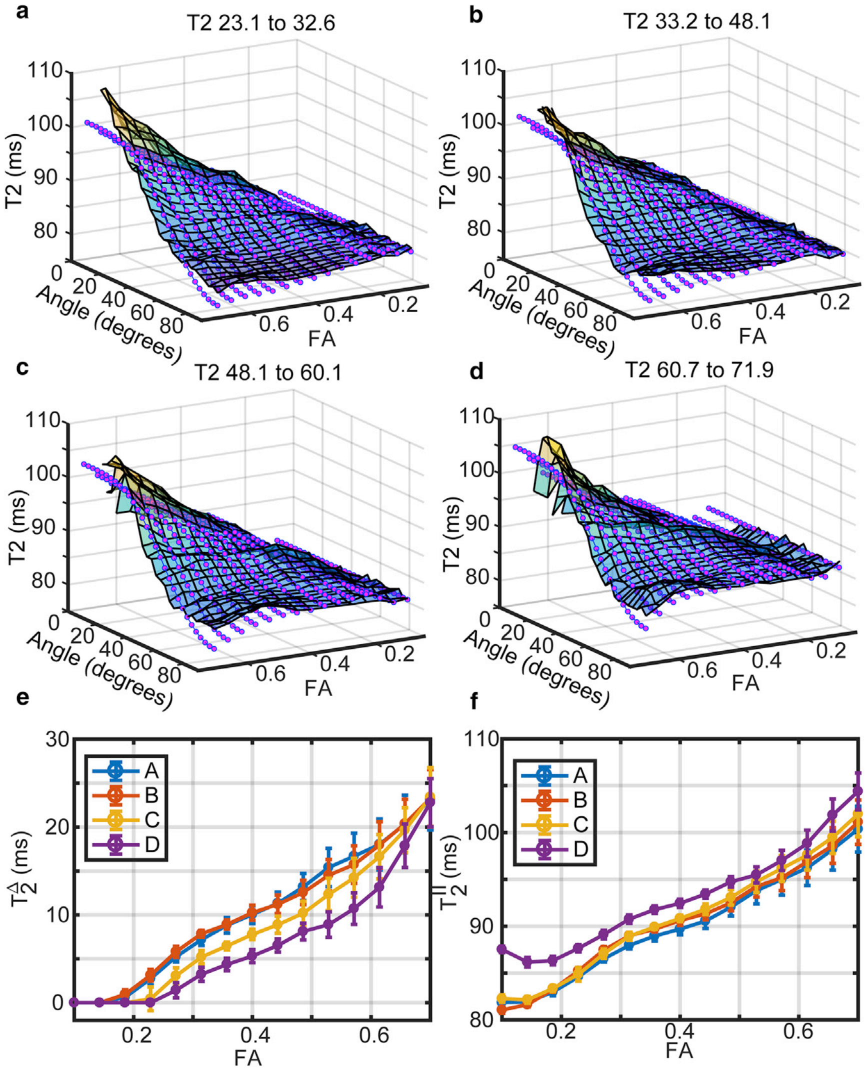

FIGURE 3 Experimental demonstration of T_2 anisotropy in human brain white matter. (*a*)–(*d*) show surfaces of the average T_2 in 2D bins according to FA and the angle between the principal axis of the diffusion tensor and B_0 in four age groups indicated above the panels. The opaque surfaces are the experimental observations; the dots the fit of Eq. 13. A general tendency toward increased T_2 and a decreased effect of anisotropy with increasing age is visible. (*e*) and (*f*) show the fitted T_2^{Δ} and parallel (isotropic) $T_2^{\parallel}$ derived from fitting Eq. 13 at each FA bin-range center value. Error bars are the 95% confidence intervals for the fit. The legend entries A, B, C, and D correspond to the respective age groups of (*a*)–(*d*). To see this figure in color, go online.

10.0 $\pm$ 1.02 ms, whereas in the oldest group of 60.7–71.9 years, it is 5.31 $\pm$ 0.75 ms. In the FA bin range 0.592–0.654, it has the value 18.02 $\pm$ 2.91 ms in the youngest age group and 13.14 $\pm$ 2.24 in the oldest. The isotropic (parallel) $T_2^{\parallel}$ is plotted as a function of FA in Fig. 3 *f*, showing its increase with age and FA. It is seen that in total, at high FA in particular, T_2 varies by up to 20 ms with θ; the angle between the principal direction of translation diffusion and B_0. The effect of anisotropy may therefore explain a significant amount of variance in the overall distribution of T_2 for WM.

A regression model for T_2: Demonstration of anisotropy

To more thoroughly examine interactions between factors such as age, MD, etc., and perform a statistical test of whether anisotropy contributes to our data we focused our attention on three major WM tracts: the corticospinal tracts (CSTs), which are early-myelinating association fibers; the superior longitudinal fasciculus (SLoF), containing later-myelinating fibers (but close to the CST), and the uncinate fasciculus (UF), containing late-myelinating fibers but in an anatomically distinct region and consistently implicated as suffering early change in dementia and cognitive decline (24). In all cases, a model including an effect of T_2 anisotropy was better able to describe the data than a model without, with the *p*-values for the amplitude of anisotropy and many interaction terms involving it zero to the limit of machine precision. Summary statistics are tabulated in the Supporting Material (Table S1). A key result is that T_2 anisotropy declines with increasing age and increases with increasing FA, which is consistent with Fig. 3. An exception, however, was the UF, in which an interaction between age and anisotropy could not be detected ($p > 0.05$ for the interaction term), such that in this tract T_2 anisotropy is less affected by age. Therefore, T_2 anisotropy also changes differently with age depending on brain region or

WM tract. A visualization of the model is provided in Fig. 4. The effect of anisotropy is clear, reflected in the high statistical significance for its inclusion and regression coefficients for the amplitude of anisotropy shown in Fig. 4 *i*. For example, the model describes the data for the entire WM skeleton with an R_2 of 0.27, compared with 0.14 without an effect of anisotropy modeled, with the regression coefficient for the amplitude of anisotropy (shown in Fig. 4 *i*) the largest of all terms (because the data were transformed to *z*-scores, coefficients are on a comparable scale). Like the experimental form (averaging over MD and bin-ranged over FA), the effect increases with increasing FA and decreases with increasing age. It is clear that the variation in T_2 explained by anisotropy is similarly significant to that explained by age. The minimum T_2 is typically passed at age ~30 (though varies a little according to interaction effects).

Demonstration of faster aging and lower anisotropy in late-myelinating tracts

Each voxel of the WM skeleton was labeled as early-myelinating or late-myelinating, to create two data sets. By fitting the regression model including the effect of T_2 anisotropy to these two data sets separately, we were able to examine differences between the aging characteristics of the two classes of WM. The results are shown in Fig. 5. From these models, and in particular inspecting the regression coefficients in Fig. 5 *e*, we can make several observations. First, although in both WM groups the T_2 increases with age, the effect of age is greater in the late-myelinating WM. This is so for the first- and second-order coefficients. Therefore, T_2 increases with age more rapidly in late-myelinating WM, especially in later life. Second, the effect of anisotropy is markedly larger in the early-myelinating WM. As such, the microstructure conferring the property of anisotropy upon T_2 is more prevalent or better-preserved with age in early-myelinating WM.

DISCUSSION

We have developed a formalism of nuclear spin phase decoherence due to mesoscopic magnetic field inhomogeneities and explored the effects on measurements of diffusion and relaxation anisotropy.

Relaxometry in microstructure imaging

The fact that T_2 is influenced by fiber orientation and the presence of crossing fibers may provide another domain in which relaxometry can contribute to microstructure imaging. Knowledge of the response of T_2 (or other relaxometric parameters) to applied field gradients and its dependence on microstructure and orientation may provide the basis for novel microstructural imaging modalities and restraints in testing experimental models. Recent work has already begun to explore the utility of other relaxometry modalities in microstructure imaging. In particular, it has been shown that T_1 relaxometry is able to quantify differential WM tract characteristics (25), including unique T_1 values for each fiber of crossing fiber populations (26).

We showed by two means that there is an effect of orientation on a voxel's T_2 in human WM, and that the effect is consistent with the theory provided. Therefore, a model of cylindrical field perturbers creating mesoscopic magnetic field inhomogeneities appears to be suitable for describing T_2 measured using a multiecho spin-echo pulse sequence in the human brain in vivo. The use of such a model extends our previous observation (12), and extends the use of a cylindrical field perturber model in frequency difference mapping (5) and QSM (6).

We have also shown an interaction between age and anisotropy. As we age, the extent to which anisotropy influences T_2 decreases. Therefore, we anticipate that contributors to mesoscopic magnetic field inhomogeneities are removed with age. Exactly what those contributors are is a largely unexplored field. The effects of age and anisotropy upon T_2 are not just tract-specific but depend on myelogenesis. We have shown that early-myelinating WM, at least within the major fiber bundles, is more "robust" to aging than late-myelinating WM when parameterized by T_2. Specifically, T_2 increases more rapidly in this cross-sectional cohort with age in late-myelinating WM, accelerating even more with age, while its anisotropy is less than that of early-myelinating WM. There is also a weaker interaction between age and T_2 anisotropy in early than late-myelinating WM. This is suggestive that T_2 mapping and the development of modalities for the mapping of its anisotropy may be powerful means of examining subtle differences in the "types" of aging that distinct categories of WM undergo. This is significant in the context of the retrogenesis hypothesis, which posits that late-myelinating WM is the most susceptible to damage in later life. There is evidence from a number of studies using DTI scalars and tractography that this is the case, and the hypothesis may also explain the disproportionate damage to WM observed in Alzheimer's disease, which may precede significant loss of gray matter. The observation of an increased rate of age-related change in T_2 of late-myelinating WM in a healthy population may therefore suggest a capacity to detect "silent" pathology before clinical presentation. The same may be true of the detection of larger interactions between age and anisotropy (driven by microstructure) in late-myelinating WM.

Brain aging at the microstructural level is not well understood (27). A number of studies have used DTI to monitor the changes in DTI scalars with age (28). Overall, DTI data converge on widespread decreases in FA in WM with age and widespread increases in diffusivities, after a peak is passed in the third or fourth decade of life, and with intertract differences (29–31). Consistent with the theory of retrogenesis (24), there is some evidence that

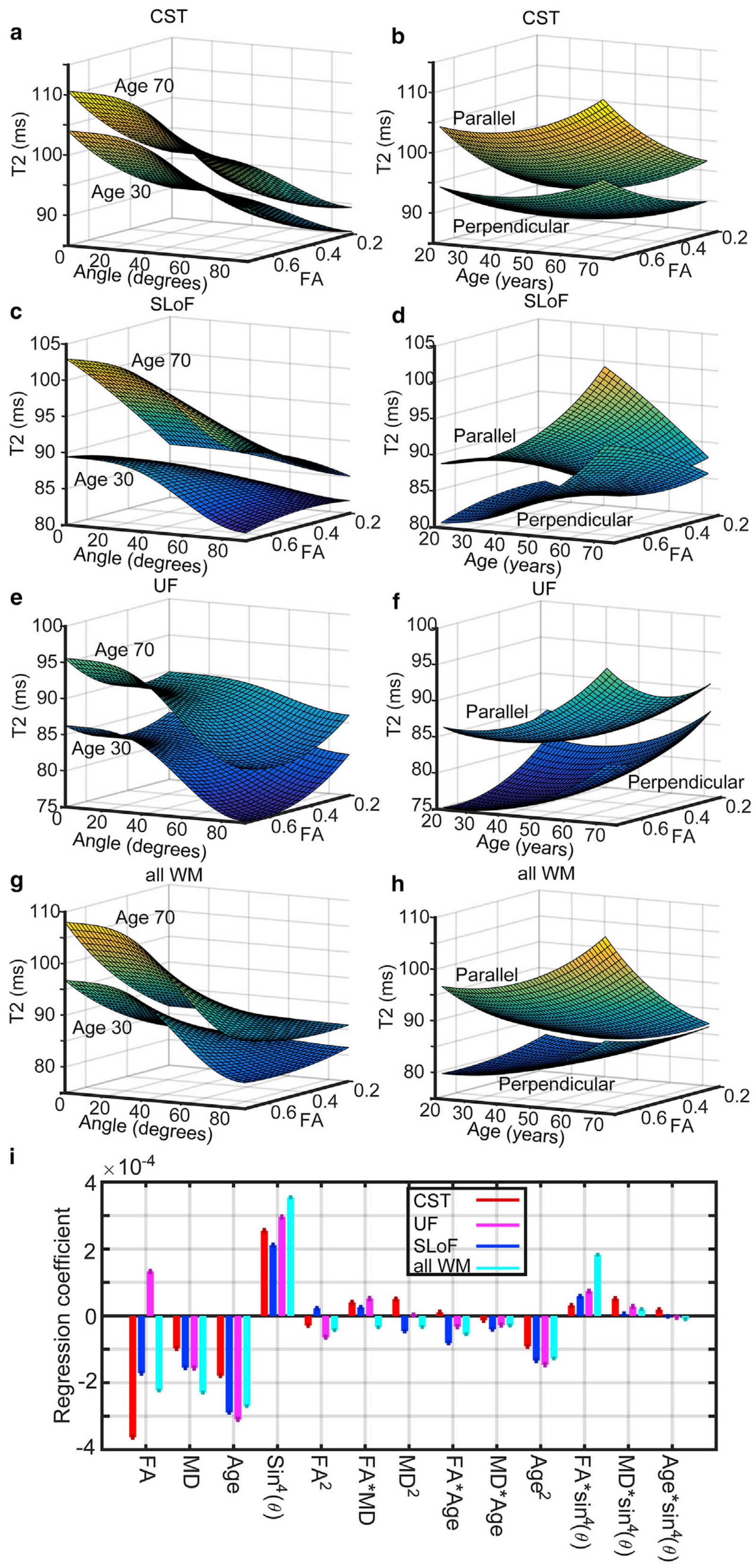

FIGURE 4 A regression model for T_2 in a cohort of healthy persons. The model used FA, MD, age, and $\sin^4\theta$ as explanatory variables of R_2 but is plotted in terms of T_2. The model shows predictions of T_2 for a fixed value of MD of 0.78×10^{-9} m^2 s^{-1} (the median across the data set). (*a*), (*c*), (*e*), and (*g*) show the dependence on FA and θ (angle), the upper surface at an age of 70, and the lower at an age of 30 (as indicated on each panel). (*b*), (*d*), (*f*), and (*h*) show the dependence of T_2 on FA and age at angles of 0 (*top surface*) and 90° (*lower surface*), labeled parallel and perpendicular, respectively. (*a*) and (*b*) are for the CST, (*c*) and (*d*) are for the SLoF, (*e*) and (*f*) are for the UF, and (*g*) and (*h*) are for anything simultaneously within the WM skeleton and JHU WM atlas. (*i*) shows the regression coefficients (fitted as *z*-scores) for each term in the model and for each region. To see this figure in color, go online.

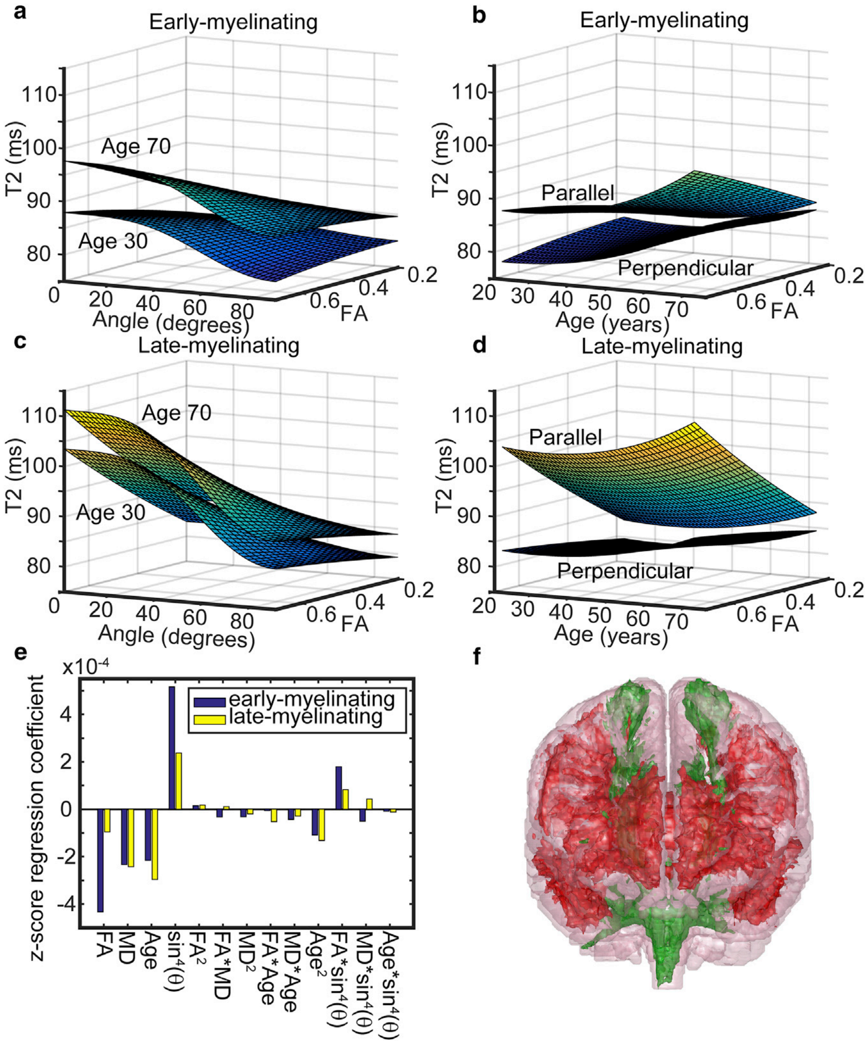

FIGURE 5 Regression model for T_2 separated into late-myelinating and early-myelinating regions of the TBSS-identified WM skeleton. The form of the fitted model is plotted at a constant MD of 0.78 × 10^{-3} mm^2 s^{-1} (the median across the data set). (*a*) and (*c*) show the dependence on FA and θ (angle), the upper surface at an age of 70 years, and the lower at an age of 30 years for early- and late-myelinating WM, respectively. (*b*) and (*d*) show the dependence of T_2 on FA and age at angles of 0 (*top surface*) and 90° (*lower surface*) for early- and late-myelinating WM, respectively. (*e*) shows fitted regression coefficients (the data were first demeaned and scaled by standard deviation). (*f*) shows the locations of the early- (*green* in online version) and late-myelinating (*red* in online version) regions, as modeled for the analysis. To see this figure in color, go online.

early-myelinating WM shows slower rates of decline when parameterized by DTI scalars. Longitudinal data similarly demonstrate widespread but subtle WM microstructural changes with age, and not explicable by loss of cortical gray matter (GM) (32,33). This is significant, for despite a shortage of empirical data (34) it has been suggested that GM loss may be causative of WM microstructural change (35), such as through Wallerian degeneration. T_2* relaxometry has also been applied to characterize age-related changes in the brain, showing decreases with age in various subcortical gray matter structures likely to generally increase in iron content with age, and limited WM regions (36). In another study, focal T_2* increases and decreases were seen in various WM regions (37).

Relation to T_2* and other literature

The effect of anisotropy is a weak one, scaling with the square of B_0, and requires some level of care to measure. Either many observations must be averaged as in the surface plots of Fig. 3 or a regression model must be fitted to a data set of appreciable size. In addition, it is likely that poor B_1 and B_0 homogeneity will confound its measurement. With these considerations, we might account for why the effect has received scant attention. A previous study sought to detect anisotropy in ex vivo bovine optic nerve at 1.5 T but reported no anisotropy (38). They did, however, report that decoherence was more rapid at early times, as predicted by our model if anisotropy be present, but used a multiexponential fit rather than Eq. 12.

Future applications

We have sought primarily to describe and explain the biophysical phenomenon of coherence lifetime anisotropy due to restricted translational diffusion through inhomogeneous magnetic fields created by biological tissues on mesoscopic (cellular) scales. There are several opportunities for exploiting such a phenomenon in both basic research and clinical applications, if routine measurement can be

brought into reality. The key applications we foresee are those involving "widespread but subtle" change on a cellular scale, in which pathology is not highly localized, or does not perturb some MR-observable parameter to such an extent as to place it within the detection limit of the human visual system. Examples may include various classes of dementia, or recovery/adaptation following stroke. We may realistically hope to provide quantitative markers of cellular-level tissue change in advance of tissue death, thus bringing forward the window of opportunity for detecting disease pathology. It may also be possible to refine estimates of quantities such as axonal packing or diameters, thereby monitoring both generation and degeneration of WM. This may be useful in determining the efficacy of some treatment (and thus guiding treatment on an individualized basis) as well as providing quantitative biomarkers useful in the development of new therapeutics aimed at preventing axonal degeneration or promoting axonal generation. This, we hope, will follow a similar pathway to application as other phenomena occurring in, or measurable by, magnetic resonance, such as diffusion anisotropy, magnetic susceptibility (and its anisotropy) and perfusion.

Limitations

Constructing a meaningful microstructure-driven model for T_2 is similarly challenging to doing so for any other parameter observable by MRI. The model is surely incomplete. However, there have been relatively few attempts at determining the effects of microstructure upon spin-echo T_2, which justifies to some extent the choice of a simple model. We were limited in experimental data by using a cross-sectional cohort. However, seeking to determine the effects of age across a broad range (49 years) does not lend itself easily to longitudinal studies, which will be necessary to fully test the predictions emerging from this work. The data acquisition and processing also suffered imperfections. The T_2 mapping was monoexponential and only made use of echoes recorded from 24 to 120 ms, the first at 12 ms being discarded since the pulse sequence used the same crushers astride each refocusing pulse, the first echo therefore being a "pure" spin echo and disproportionately low in intensity. However, this means that we are sensitive only to relatively slow-decaying coherence. This means we could not fit Eq. 12 directly to data. Neither could we fit models in which the effects of susceptibility differences are assumed not to contribute but the isotropic T_2 is assumed to be different in the vicinity of the myelin sheath, such as multiexponential decoherence models. We have yet to address experimentally the issue of crossing fibers, instead simply limiting the analysis to the major fiber bundles identified by TBSS (in which crossing fibers are still likely to be a confound).

CONCLUSIONS

In conclusion, it is most likely that the MRI T_2 is influenced by microstructure, and we have proposed a means to make that influence tractable, adding T_2 mapping to the range of microstructure imaging modalities. By understanding the physical basis of microstructural modulation of the MRI signal, we hope to make challenging matters of human health and disease tractable. As a demonstration, we have shown that the T_2 and its anisotropy are differently affected by age, and that late-myelinating WM is more susceptible to the effects of age.

SUPPORTING MATERIAL

Supporting Materials and Methods, one figure, and two tables are available at http://www.biophysj.org/biophysj/supplemental/S0006-3495(17)30240-0.

AUTHOR CONTRIBUTIONS

M.J.K. designed the experiments, collected and analyzed data, and wrote the manuscript. S.D. and L.J. collected the data. R.A.K. wrote the manuscript.

ACKNOWLEDGMENTS

M.J.K. is funded by the Elizabeth Blackwell Institute and by the Wellcome Trustinternational strategic support fund (ISSF2: 105612/Z/14/Z).

REFERENCES

1. Basser, P. J., J. Mattiello, and D. LeBihan. 1994. MR diffusion tensor spectroscopy and imaging. *Biophys. J.* 66:259–267.
2. Jones, D. K. 2011. Diffusion MRI. Oxford University Press, Oxford, UK.
3. Majumdar, S., and J. C. Gore. 1988. Studies of diffusion in random fields produced by variations in susceptibility. *J. Magn. Reson.* 78:41–55.
4. Yablonskiy, D. A., and E. M. Haacke. 1994. Theory of NMR signal behavior in magnetically inhomogeneous tissues: the static dephasing regime. *Magn. Reson. Med.* 32:749–763.
5. Wharton, S., and R. Bowtell. 2012. Fiber orientation-dependent white matter contrast in gradient echo MRI. *Proc. Natl. Acad. Sci. USA.* 109:18559–18564.
6. Wharton, S., and R. Bowtell. 2015. Effects of white matter microstructure on phase and susceptibility maps. *Magn. Reson. Med.* 73:1258–1269.
7. Buschle, L. R., F. T. Kurz, ..., C. H. Ziener. 2015. Diffusion-mediated dephasing in the dipole field around a single spherical magnetic object. *Magn. Reson. Imaging.* 33:1126–1145.
8. Haacke, E. M., S. Liu, ..., Y. Ye. 2015. Quantitative susceptibility mapping: current status and future directions. *Magn. Reson. Imaging.* 33:1–25.
9. Reichenbach, J. R., F. Schweser, ..., A. Deistung. 2015. Quantitative susceptibility mapping: concepts and applications. *Clin. Neuroradiol.* 25 (*Suppl. 2*):225–230.
10. Liu, C. 2010. Susceptibility tensor imaging. *Magn. Reson. Med.* 63:1471–1477.

11. Li, W., B. Wu, …, C. Liu. 2012. Magnetic susceptibility anisotropy of human brain in vivo and its molecular underpinnings. *Neuroimage.* 59:2088–2097.

12. Knight, M. J., B. Wood, …, R. A. Kauppinen. 2015. Anisotropy of spin-echo T2 relaxation by magnetic resonance imaging in the human brain in vivo. *Biomed. Spectrosc. Imaging.* 4:299–310.

13. Knight, M. J., and R. A. Kauppinen. 2016. Diffusion-mediated nuclear spin phase decoherence in cylindrically porous materials. *J. Magn. Reson.* 269:1–12.

14. Reisberg, B., E. H. Franssen, …, S. Kenowsky. 2002. Evidence and mechanisms of retrogenesis in Alzheimer's and other dementias: management and treatment import. *Am. J. Alzheimers Dis. Other Demen.* 17:202–212.

15. Kuchel, P. W., G. Pagès, …, K. H. Chuang. 2012. Stejskal–tanner equation derived in full. *Concepts Magn. Reson. Part A.* 40A:205–214.

16. Feinberg, D. A., S. Moeller, …, E. Yacoub. 2010. Multiplexed echo planar imaging for sub-second whole brain FMRI and fast diffusion imaging. *PLoS One.* 5:e15710.

17. Andersson, J. L., and S. N. Sotiropoulos. 2016. An integrated approach to correction for off-resonance effects and subject movement in diffusion MR imaging. *Neuroimage.* 125:1063–1078.

18. Andersson, J. L., S. Skare, and J. Ashburner. 2003. How to correct susceptibility distortions in spin-echo echo-planar images: application to diffusion tensor imaging. *Neuroimage.* 20:870–888.

19. Smith, S. M., M. Jenkinson, …, T. E. Behrens. 2006. Tract-based spatial statistics: voxelwise analysis of multi-subject diffusion data. *Neuroimage.* 31:1487–1505.

20. Smith, S. M., M. Jenkinson, …, P. M. Matthews. 2004. Advances in functional and structural MR image analysis and implementation as FSL. *Neuroimage.* 23 (*Suppl. 1*):S208–S219.

21. Wakana, S., A. Caprihan, …, S. Mori. 2007. Reproducibility of quantitative tractography methods applied to cerebral white matter. *Neuroimage.* 36:630–644.

22. Hua, K., J. Zhang, …, S. Mori. 2008. Tract probability maps in stereotaxic spaces: analyses of white matter anatomy and tract-specific quantification. *Neuroimage.* 39:336–347.

23. Kinney, H. C., B. A. Brody, …, F. H. Gilles. 1988. Sequence of central nervous system myelination in human infancy. II. Patterns of myelination in autopsied infants. *J. Neuropathol. Exp. Neurol.* 47:217–234.

24. Alves, G. S., V. Oertel Knöchel, …, J. Laks. 2015. Integrating retrogenesis theory to Alzheimer's disease pathology: insight from DTI-TBSS investigation of the white matter microstructural integrity. *BioMed Res. Int.* 2015:291658.

25. De Santis, S., Y. Assaf, …, A. Roebroeck. 2016. T1 relaxometry of crossing fibres in the human brain. *Neuroimage.* 141:133–142.

26. De Santis, S., D. Barazany, …, Y. Assaf. 2016. Resolving relaxometry and diffusion properties within the same voxel in the presence of crossing fibres by combining inversion recovery and diffusion-weighted acquisitions. *Magn. Reson. Med.* 75:372–380.

27. Bennett, I. J., and D. J. Madden. 2014. Disconnected aging: cerebral white matter integrity and age-related differences in cognition. *Neuroscience.* 276:187–205.

28. Yap, Q. J., I. Teh, …, K. Sim. 2013. Tracking cerebral white matter changes across the lifespan: insights from diffusion tensor imaging studies. *J. Neural. Transm. (Vienna).* 120:1369–1395.

29. Miller, K. L., F. Alfaro-Almagro, …, S. M. Smith. 2016. Multimodal population brain imaging in the UK Biobank prospective epidemiological study. *Nat. Neurosci.* 19:1523–1536.

30. Kochunov, P., D. E. Williamson, …, D. C. Glahn. 2012. Fractional anisotropy of water diffusion in cerebral white matter across the lifespan. *Neurobiol. Aging.* 33:9–20.

31. Westlye, L. T., K. B. Walhovd, …, A. M. Fjell. 2010. Life-span changes of the human brain white matter: diffusion tensor imaging (DTI) and volumetry. *Cereb. Cortex.* 20:2055–2068.

32. Storsve, A. B., A. M. Fjell, …, K. B. Walhovd. 2016. Longitudinal changes in white matter tract integrity across the adult lifespan and its relation to cortical thinning. *PLoS One.* 11:e0156770.

33. Sexton, C. E., K. B. Walhovd, …, A. M. Fjell. 2014. Accelerated changes in white matter microstructure during aging: a longitudinal diffusion tensor imaging study. *J. Neurosci.* 34:15425–15436.

34. Mesulam, M. 2012. The evolving landscape of human cortical connectivity: facts and inferences. *Neuroimage.* 62:2182–2189.

35. Conforti, L., J. Gilley, and M. P. Coleman. 2014. Wallerian degeneration: an emerging axon death pathway linking injury and disease. *Nat. Rev. Neurosci.* 15:394–409.

36. Callaghan, M. F., P. Freund, …, N. Weiskopf. 2014. Widespread age-related differences in the human brain microstructure revealed by quantitative magnetic resonance imaging. *Neurobiol. Aging.* 35:1862–1872.

37. Draganski, B., J. Ashburner, …, N. Weiskopf. 2011. Regional specificity of MRI contrast parameter changes in normal ageing revealed by voxel-based quantification (VBQ). *Neuroimage.* 55:1423–1434.

38. Henkelman, R. M., G. J. Stanisz, …, M. J. Bronskill. 1994. Anisotropy of NMR properties of tissues. *Magn. Reson. Med.* 32:592–601.

Biophysical Journal, Volume *112*

Supplemental Information

Magnetic Resonance Relaxation Anisotropy: Physical Principles and Uses in Microstructure Imaging

Michael J. Knight, Serena Dillon, Lina Jarutyte, and Risto A. Kauppinen

Supplementary information

Mathematical details

We wish to calculate the form of the time-dependent spin phase decoherence due to generalised but small mesoscopic magnetic field inhomogeneities. The master equation is the Bloch-Torrey equation (1-3) for transverse magnetisation with a generalised but small magnetic field inhomogeneity(4):

$$\frac{\partial}{\partial t}M^{+}(t,\mathbf{x})=\left(-i\omega_0-i\omega_{cs}-i\omega_I(\mathbf{x})-R_2(\mathbf{x})+\nabla\cdot\mathbf{D}(\mathbf{x})\nabla\right)M^{+}(t,\mathbf{x}) \quad (1)$$

Here, $M^{+}(t,\mathbf{x})$ is the complex-valued transverse magnetisation as a function of time *t* and spatial coordinate **x**, ω_0 is the Larmor frequency, ω_{cs} is the isotropic part of the chemical shift anisotropy tensor, $\omega_I(\mathbf{x})$ is a frequency inhomogeneity function, $R_2(\mathbf{x})$ is the (isotropic) transverse relaxation rate coefficient scalar field, ∇ is the gradient operator, and $\mathbf{D}(\mathbf{x})$ the translational diffusion tensor field. In our original paper, we showed that the signal, in a demodulated frame rotated with the chemically shifted Larmor frequency, would evolve according to (2)

$$S(t)=\left|\int A_0(\mathbf{x})\exp\left\{\frac{i\rho t^2}{2}\left[\nabla\cdot\mathbf{D}(\mathbf{x})\nabla\right]\omega_I(\mathbf{x})\right\}\exp\left\{-\frac{\rho^2t^3}{3}\left[\nabla\omega_I\cdot\mathbf{D}(\mathbf{x})\nabla\right]\omega_I(\mathbf{x})\right\}\exp\left(-R_2(\mathbf{x})t\right)d\mathbf{x}\right|$$

If we are performing a spin-echo experiment, assuming that phase terms may be entirely refocussed, this reduces to

$$S(t)=\left|\int A_0(\mathbf{x})\exp\left\{-\frac{\rho^2t^3}{3}\left[\nabla\omega_I(\mathbf{x})\cdot\mathbf{D}(\mathbf{x})\nabla\right]\omega_I(\mathbf{x})\right\}\exp\left(-R_2(\mathbf{x})t\right)d\mathbf{x}\right| \quad (3)$$

Where, in both cases, ρ is the coherence order and A_0 the signal amplitude at t=0. We will henceforth restrict the discussion to spin-echo experiments and neglect phase terms.

We can express this more simply as

$$S(t)=\left|\int A_0\exp\left(-\mathbf{b}\cdot\mathbf{D}\right)\exp\left(-R_2t\right)d\mathbf{x}\right| \quad (4)$$

In which the b-tensor field **b** has been introduced. The quantity **b.D** represents the sum of element-wise products evaluated at coordinate **x**. The b-tensor field is defined as

$$b_{jk}\left(\mathbf{x}\right)=\frac{\rho^2 t^3}{3}\frac{\partial\omega_I\left(\mathbf{x}\right)}{\partial x_j}\frac{\partial\omega_I\left(\mathbf{x}\right)}{\partial x_k} \tag{5}$$

In which $\omega_I\left(\mathbf{x}\right)$ is an inhomogeneous contribution to the resonance frequency experienced by the nuclear species under observation at coordinate **x**. This generalises the b-value used in diffusion imaging if the frequency inhomogeneity is linear:

$$b_{jk}=\frac{t^3}{3}\gamma^2 G_j G_k \tag{6}$$

With G_j, G_k elements of an applied (linear) magnetic field gradient.

Elements of the b-tensor field

The b-tensor expansion may be equivalently expressed:

$$\begin{aligned}\frac{\rho^2 t^3}{3}\left[\nabla\omega_I\left(\mathbf{x}\right)\cdot\mathbf{D}\left(\mathbf{x}\right)\nabla\right]\omega_I\left(\mathbf{x}\right)&=\frac{\rho^2 t^3}{3}\sum_m\sum_{j,k}\frac{\partial\omega_I^{(m)}\left(\mathbf{x}\right)}{\partial x_j}\frac{\partial\omega_I^{(m)}\left(\mathbf{x}\right)}{\partial x_k}D_{jk}^{(m)}\left(\mathbf{x}\right)\\&=\sum_m\sum_{j,k}b_{jk}^{(m)}\left(\mathbf{x}\right)D_{jk}^{(m)}\left(\mathbf{x}\right)\\&=\sum_m\mathbf{b}^{(m)}\cdot\mathbf{D}^{(m)}\\&=\mathbf{b}\cdot\mathbf{D}\end{aligned} \tag{7}$$

Or, as an expansion into the contributions of the susceptibility and applied field gradient effects:

$$\begin{aligned}\mathbf{b}\cdot\mathbf{D}&=\frac{\rho^2 t^3}{3}\sum_{j,k}\frac{\partial\left(\Delta\omega+\omega_D\right)}{\partial x_j}\frac{\partial\left(\Delta\omega+\omega_D\right)}{\partial x_k}D_{jk}\\&=\frac{\rho^2 t^3}{3}\sum_{j,k}\left[\frac{\partial\Delta\omega}{\partial x_j}\frac{\partial\Delta\omega}{\partial x_k}+\frac{\partial\omega_D}{\partial x_j}\frac{\partial\omega_D}{\partial x_k}+\frac{\partial\Delta\omega}{\partial x_j}\frac{\partial\omega_D}{\partial x_k}+\frac{\partial\omega_D}{\partial x_j}\frac{\partial\Delta\omega}{\partial x_k}\right]D_{jk}\\&=\frac{\rho^2 t^3}{3}\begin{bmatrix}\left[\nabla\Delta\omega\right]^T\cdot\mathbf{D}\nabla\Delta\omega\\+\left[\nabla\omega_D\right]^T\mathbf{D}\nabla\omega_D\\+\left(\left[\nabla\Delta\omega\right]^T\cdot\mathbf{D}\nabla\omega_D+\left[\nabla\omega_D\right]^T\cdot\mathbf{D}\nabla\Delta\omega\right)\end{bmatrix}\end{aligned} \tag{8}$$

Where the superscript *T* represents the transpose operation.

The cylindrical model

In the walled cylinder model, the frequency inhomogeneity function takes the form

$$\Delta\omega(\mathbf{x}) = \sum_m \begin{cases} \frac{\omega_0\chi_m}{2}\sin^2\theta_m \cos 2\phi\left(\frac{r_{cm}^2}{r^2}\right), & r \ge r_{cm} \\ \frac{\omega_0\chi_m}{2}\left(\cos^2\theta_m - \frac{1}{3} - \sin^2\theta_m \cos 2\phi\left(\frac{r_{cm}^2 - r_{Lm}^2}{r^2}\right)\right), & r_{Lm} \le r < r_{cm} \\ 0, & r < r_{Lm} \end{cases} \tag{9}$$

Where ω_0 is the Larmor frequency, θ is the polar angle between the long axis of the cylinder *j* and B_0, and the coordinates ϕ,*r* represent position in a cylindrical system with the z-axis parallel to the cylinder long axis and B_0 defined in the *xz* plane. This is the cylinder principal axis system (PAS). χ is the susceptibility difference (with the susceptibility tensor assumed isotropic) between the wall of the cylinder and outside, r_c is the cylinder outer radius radius, r_L the lumen radius. This frequency difference is present as long as the system is subject to an applied magnetic field B_0. The diffusion tensor field is treated such that each cylinder lumen has its own diffusion tensor, each cylinder wall (if applicable) has its own diffusion tensor and the surroundings have a unique diffusion tensor. We can therefore write

$$D_{jk}^{(m)} = \begin{cases} D_{jk}^{(m,out)}, & r \ge r_{cj} \\ D_{jk}^{(m,wall)}, & r_{Lj} \le r < r_{cj} \\ D_{jk}^{(m,lumen)}, & r < r_{Lj} \end{cases} \tag{10}$$

Although we treat the region outside the cylinders as having a single diffusion tensor, it may be that the cylinders have different orientations, such that the mth representation is in a different frame (PAS of cylinder m). For the *b*-tensor field, this leads to the “alphabet” of terms:

$$\begin{aligned} \mathbf{b} &= \sum_m \mathbf{b}^{(m)} \\ &= \mathbf{b}_A + \mathbf{b}_B + \mathbf{b}_C + \mathbf{b}_E + \mathbf{b}_F + \mathbf{b}_G \end{aligned} \tag{11}$$

Where

$$\mathbf{b}_A = \frac{\rho^2 t^3}{3} \sum_m \left[\nabla \Delta \omega_{out}^{(m)} \right]^T \nabla \Delta \omega_{out}^{(m)}$$

$$\mathbf{b}_B = \frac{\rho^2 t^3}{3} \sum_m \left[\nabla \Delta \omega_{wall}^{(m)} \right]^T \nabla \Delta \omega_{wall}^{(m)}$$

$$\mathbf{b}_{C,E,F} = \frac{\rho^2 t^3}{3} \sum_m \left[\nabla \omega_D^{(m)} \right]^T \nabla \omega_D^{(m)} \tag{12}$$

$$\mathbf{b}_G = \frac{\rho^2 t^3}{3} \sum_m \left(\left[\nabla \Delta \omega_{out}^{(m)} \right]^T \nabla \omega_D^{(m)} + \left[\nabla \omega_D^{(m)} \right]^T \nabla \Delta \omega_{out}^{(m)} \right)$$

$$\mathbf{b}_H = \frac{\rho^2 t^3}{3} \sum_m \left(\left[\nabla \Delta \omega_{wall}^{(m)} \right]^T \nabla \omega_D^{(m)} + \left[\nabla \omega_D^{(m)} \right]^T \nabla \Delta \omega_{wall}^{(m)} \right)$$

The full forms for the cylindrical model are:

$$\mathbf{b}_A = \frac{\rho^2 t^3}{3} \sum_m \frac{\chi_m^2 \omega_0^2 r_{cm}^4 \sin^4 \theta_m}{r^6} \begin{bmatrix} \cos 2\phi & \cos 2\phi \sin 2\phi & 0 \\ \cos 2\phi \sin 2\phi & \sin 2\phi & 0 \\ 0 & 0 & 0 \end{bmatrix} \tag{13}$$

$$\mathbf{b}_B = \frac{\rho^2 t^3}{3} \sum_m \frac{\chi_m^2 \omega_0^2 \left(r_{cm}^2 - r_{Lm}^2 \right)^2 \sin^4 \theta_m}{r^6} \begin{bmatrix} \cos 2\phi & \cos 2\phi \sin 2\phi & 0 \\ \cos 2\phi \sin 2\phi & \sin 2\phi & 0 \\ 0 & 0 & 0 \end{bmatrix} \tag{14}$$

The b-tensor field for C,E,F is simply the b-value as used in the ordinary theory of diffusion-weighted imaging with the diffusion tensor represented for the appropriate compartment, so expressions may be found elsewhere.

$$\mathbf{b}_G = \frac{\rho^2 t^3 \gamma \omega_0}{3} \sum_m \frac{\chi_m r_{cm}^2 \sin^2 \theta_m}{r^3} \mathbf{W} \tag{15}$$

$$\mathbf{b}_H = \frac{\rho^2 t^3 \gamma \omega_0}{3} \sum_m \frac{\chi_m \left(r_{cm}^2 - r_{Lm}^2 \right) \sin^2 \theta_m}{r^3} \mathbf{W} \tag{16}$$

Where

$$\mathbf{W} = \begin{bmatrix} 2\cos 2\phi \left(G_x \cos\phi + G_y \sin\phi \right) & -\left(G_x \sin\phi + G_y \cos\phi \right) & G_z \cos 2\phi \\ -\left(G_x \sin\phi + G_y \cos\phi \right) & 2\sin 2\phi \left(-G_x \sin\phi + G_y \cos\phi \right) & G_z \sin 2\phi \\ G_z \cos 2\phi & G_z \sin 2\phi & 0 \end{bmatrix} \tag{17}$$

These expressions are for cylindrical perturber geometry, but valid for any diffusion tensor. In the main text, we restricted the discussion to axially symmetric diffusion tensors with their unique axis parallel to that of the perturber to which they correspond, and isotropic diffusion outside the perturber region.

For computational efficiency, our simulator calculates the terms:

$$\begin{aligned}\frac{\rho^2 t^3}{3}\left[\nabla\omega_I \cdot \mathbf{D}\nabla\right]\omega_I &= \mathbf{b}\cdot\mathbf{D} \\ &= \frac{\rho^2 t^3}{3}\left(A+B+C+E+F+G+H\right)\end{aligned} \tag{18}$$

Where

$$\begin{aligned}
A &= \sum_m \nabla\Delta\omega_{out}^{(m)}\mathbf{D}_{out}^{(m)}\nabla\Delta\omega_{out}^{(m)} \\
B &= \sum_m \nabla\Delta\omega_{wall}^{(m)}\mathbf{D}_{wall}^{(m)}\nabla\Delta\omega_{wall}^{(m)} \\
C &= \nabla\omega_D \mathbf{D}_{out}\nabla\omega_D \\
E &= \sum_m \nabla\omega_D^{(m)}\mathbf{D}_{wall}^{(m)}\nabla\omega_D^{(m)} \\
F &= \sum_m \nabla\omega_D^{(m)}\mathbf{D}_{lumen}^{(m)}\nabla\omega_D^{(m)} \\
G &= \sum_m \left(\nabla\Delta\omega_{out}^{(m)}\mathbf{D}_{out}^{(m)}\nabla\omega_D^{(m)} + \nabla\omega_D^{(m)}\mathbf{D}_{out}^{(m)}\nabla\Delta\omega_{out}^{(m)}\right) \\
H &= \sum_m \left(\nabla\Delta\omega_{wall}^{(m)}\mathbf{D}_{wall}^{(m)}\nabla\omega_D^{(m)} + \nabla\omega_D^{(m)}\mathbf{D}_{wall}^{(m)}\nabla\Delta\omega_{wall}^{(m)}\right)
\end{aligned} \tag{19}$$

These correspond to $\mathbf{b}_{\boldsymbol{A}}$ etc from the main text. Thus equipped, we may write out all the above dephasing terms fully as:

$$A = \sum_m \frac{\chi_m^2\omega_0^2 r_{cm}^4 \sin^4\theta_m}{r^6}\left[\begin{array}{l}\left(2\cos^2\phi - 1\right)^2\left(D_{11}^{(m)}\cos^2\phi + D_{22}^{(m)}\sin^2\phi + 2D_{12}^{(m)}\sin\phi\cos\phi\right) + \\ 4\cos^2\phi\sin^2\phi\left(D_{11}^{(m)}\sin^2\phi + D_{22}^{(m)}\cos^2\phi - 2D_{12}^{(m)}\sin\phi\cos\phi\right) + \\ 2\cos\phi\sin\phi\left(-D_{11}^{(m)}\frac{\sin 2\phi}{2} + D_{22}^{(m)}\frac{\cos 2\phi}{2} + D_{12}^{(m)}\cos 2\phi\right)\end{array}\right] \tag{20}$$

Where the elements $D_{jk}^{(m)}$ are of the diffusion tensor for the region outside the perturbers in Cartesian representation transformed into in the “cylinder PAS” of perturber *m*.

$$B=\sum_m \frac{\chi_m^2\omega_0^2\left(r_{cm}^2-r_{Lm}^2\right)^2\sin^4\theta_m}{r^6}\left[\begin{array}{l}\left(2\cos^2\phi-1\right)^2\left(D_{11}^{(m)}\cos^2\phi+D_{22}^{(m)}\sin^2\phi+2D_{12}^{(m)}\sin\phi\cos\phi\right)+\\ 4\cos^2\phi\sin^2\phi\left(D_{11}^{(m)}\sin^2\phi+D_{22}^{(m)}\cos^2\phi-2D_{12}^{(m)}\sin\phi\cos\phi\right)+\\ 2\cos\phi\sin\phi\left(-D_{11}^{(m)}\frac{\sin 2\phi}{2}+D_{22}^{(m)}\frac{\cos 2\phi}{2}+D_{12}^{(m)}\cos 2\phi\right)\end{array}\right]$$

(21)

Where the elements $D_{jk}^{(m)}$ are of the diffusion tensor for the wall of perturber *m* in the cylinder PAS of perturber *m*, in Cartesian representation.

$$C=\gamma^2\left(D_{11}G_x^2+D_{22}G_y^2+D_{33}G_z^2+2D_{12}G_xG_y+2D_{13}G_xG_z+2D_{23}G_yG_z\right) \quad (22)$$

Where the diffusion tensor is for the region outside the perturbers, may have a single reference frame provided the pulsed field gradient G is also in that frame and is in Cartesian representation.

$$E=\gamma^2\sum_m\left(D_{11}^{(m)}G_x^2+D_{22}^{(m)}G_y^2+D_{33}^{(m)}G_z^2+2D_{12}^{(m)}G_xG_y+2D_{13}^{(m)}G_xG_z+2D_{23}^{(m)}G_yG_z\right) \quad (23)$$

Where the elements $D_{jk}^{(m)}$ are of the diffusion tensor for the wall of perturber *m* in the cylinder PAS of perturber *m*, in Cartesian representation. The pulsed field gradient **G** should also be represented in that frame.

$$F=\gamma^2\sum_m\left(D_{11}^{(m)}G_x^2+D_{22}^{(m)}G_y^2+D_{33}^{(m)}G_z^2+2D_{12}^{(m)}G_xG_y+2D_{13}^{(m)}G_xG_z+2D_{23}^{(m)}G_yG_z\right) \quad (24)$$

Where the elements $D_{jk}^{(m)}$ are of the diffusion tensor for the lumen of perturber *m* in the cylinder PAS of perturber *m*, in Cartesian representation. The pulsed field gradient **G** should also be represented in that frame.

$$G=2\gamma\omega_0\sum_m\frac{\chi_m r_{cm}^2}{r^3}\sin^2\theta_m\left(\begin{array}{l}\cos 3\phi\left(G_xD_{11}^{(m)}+G_yD_{12}^{(m)}+G_zD_{13}^{(m)}\right)+\\ \sin 3\phi\left(G_xD_{12}^{(m)}+G_yD_{22}^{(m)}+G_zD_{23}^{(m)}\right)\end{array}\right) \quad (25)$$

Where the elements $D_{jk}^{(m)}$ are of the diffusion tensor for the region outside the perturbers in Cartesian representation transformed into in the “cylinder PAS” of perturber *m*. The elements of pulsed field gradient **G** must be represented in the same frame.

$$H = 2\gamma\omega_0 \sum_m \frac{\chi_m \left(r_{cm}^2 - r_{Lm}^2\right)}{r^3} \sin^2\theta_m \begin{pmatrix} \cos 3\phi \left(G_x D_{11}^{(m)} + G_y D_{12}^{(m)} + G_z D_{13}^{(m)}\right) + \\ \sin 3\phi \left(G_x D_{12}^{(m)} + G_y D_{22}^{(m)} + G_z D_{23}^{(m)}\right) \end{pmatrix} \tag{26}$$

Where the elements $D_{jk}^{(m)}$ are of the diffusion tensor for the wall of perturber *m* in the cylinder PAS of perturber *m*, in Cartesian representation. The pulsed field gradient **G** should also be represented in that frame.

Simplified model

In the main text we use a simplified model in which diffusion outside the perturber region is isotropic, and in any perturber wall or lumen is axially symmetric with the unique axis parallel to the cylinder axis. The reduced expressions for the *b*-tensor alphabet, multiplied by the diffusion tensor, then become:

$$\mathbf{b}_A^{(m)} \cdot \mathbf{D}_{out}^{(m)} = \frac{\rho^2 t^3}{3} \frac{\chi_m^2 \omega_0^2 r_{cm}^4 \sin^4\theta_m}{r^6} D_{out} \tag{27}$$

where D_{out} is the isotropic diffusion coefficient of the space outside any perturbers.

$$\mathbf{b}_B^{(m)} \cdot \mathbf{D}_{out}^{(m)} = \frac{\rho^2 t^3}{3} \frac{\chi_m^2 \omega_0^2 \left(r_{cm}^2 - r_{Lm}^2\right)^2 \sin^4\theta_m}{r^6} D_{wall,R}^{(m)} \tag{28}$$

where $D_{wall,R}^{(m)}$ is the radial diffusivity in the wall of perturber *m*.

$$\mathbf{b}_C^{(m)} \cdot \mathbf{D}_{out}^{(m)} = \frac{\rho^2 t^3 \gamma^2}{3} D_{out} \left(G_x^2 + G_y^2 + G_z^2\right) \tag{29}$$

$$\mathbf{b}_E^{(m)} \cdot \mathbf{D}_{wall}^{(m)} = \frac{\rho^2 t^3 \gamma^2}{3} \left(D_{wall,R}^{(m)} \left(G_x^2 + G_y^2\right) + D_{wall,A}^{(m)} G_z^2\right) \tag{30}$$

Where $D_{wall,A}^{(m)}$ is the axial diffusivity of the wall of perturber *m*.

$$\mathbf{b}_F^{(m)} \cdot \mathbf{D}_{lumen}^{(m)} = \frac{\rho^2 t^3 \gamma^2}{3} \left(D_{lumen,R}^{(m)} \left(G_x^2 + G_y^2\right) + D_{lumen,A}^{(m)} G_z^2\right) \tag{31}$$

Where $D_{lumen,R}^{(m)}$ and $D_{lumen,A}^{(m)}$ are the radial and axial diffusivities in the lumen of perturber *m*.

$$\mathbf{b}_G^{(m)} \cdot \mathbf{D}_{out}^{(m)} = \frac{2\rho^2 t^3 \gamma\omega_0}{3} \frac{\chi_m r_{cm}^2 \sin^2\theta_m}{r^3} D_{out} \left(G_x \cos 3\phi + G_y \sin 3\phi\right) \tag{32}$$

$$\mathbf{b}_H^{(m)} \cdot \mathbf{D}_{lumen}^{(m)} = \frac{2\rho^2 t^3 \gamma\omega_0}{3} \frac{\chi_m \left(r_{cm}^2 - r_{Lm}^2\right)\sin^2\theta_m}{r^3} D_{lumen,R}^{(m)} \left(G_x \cos 3\phi + G_y \sin 3\phi\right) \tag{33}$$

Derivatives and coordinate systems

We must have a coordinate system and representation. Calculations are most easily performed in a coordinate system in which the z-axis of a given cylinder lies along the z-axis and B_0 lies in the xz plane. The most appropriate coordinate representation is cylindrical. The diffusion tensors and gradient operator must therefore be represented using the cylindrical system. The gradient operator in cylindrical coordinates is given by

$$\nabla = \begin{bmatrix} \frac{\partial}{\partial r} \\ \frac{1}{r}\frac{\partial}{\partial \phi} \\ \frac{\partial}{\partial z} \end{bmatrix} \tag{34}$$

The transformation between Cartesian and cylindrical coordinates is made through

$$D^{cyl} = \hat{Q}^T D^{Car} \hat{Q} \tag{35}$$

Where the transformation tensor is given by

$$\hat{Q} = \begin{pmatrix} \cos\phi & -\sin\phi & 0 \\ \sin\phi & \cos\phi & 0 \\ 0 & 0 & 1 \end{pmatrix} \tag{36}$$

This transformation is useful, as it is more convenient in simulations to enter diffusion tensors for each cylinder wall/lumen in Cartesian for in the principal axis system of that cylinder.

The necessary derivatives, using the cylindrical gradient operator, are given for cylindrical perturbers with walls of finite thickness by:

$$\nabla\Delta\omega_{out}^{(m)} = \begin{bmatrix} -\omega_0 \chi_m \sin^2\theta_m \cos 2\phi \dfrac{r_{cm}^2}{r^3} \\ -\omega_0 \chi_m \sin^2\theta_m \sin 2\phi \dfrac{r_{cm}^2}{r^3} \\ 0 \end{bmatrix} \tag{37}$$

$$\nabla\Delta\omega_{wall}^{(m)} = \begin{bmatrix} -\omega_0 \chi_m \sin^2\theta_m \cos 2\phi \dfrac{r_{cm}^2 - r_{Lm}^2}{r^3} \\ -\omega_0 \chi_m \sin^2\theta_m \sin 2\phi \dfrac{r_{cm}^2 - r_{Lm}^2}{r^3} \\ 0 \end{bmatrix} \tag{38}$$

$$\nabla\Delta\omega_{lumen}^{(m)} = \begin{bmatrix} 0 \\ 0 \\ 0 \end{bmatrix} \tag{39}$$

For the PFG terms:

$$\nabla\omega_D = -\gamma \begin{bmatrix} G_x \cos\phi + G_y \sin\phi \\ G_x \cos\phi - G_y \sin\phi \\ G_z z \end{bmatrix} \tag{40}$$

All these terms are given in the PAS of a particular perturber. So, for example, the expression for *A* requires that the diffusion tensor field outside the perturber region be transformed into the PAS of each cylinder before application of that expression.

Extended methods

Simulations

The tensor calculations of the theory section were performed with the aid of the Matlab Symbolic Math Toolbox and simulations of diffusion-mediated dephasing performed using Matlab 2015b. A set of classes were written for this purpose, which could calculate all the terms given in the theory section as well as determine effective diffusion tensors and T_2 from simulations including applied filed gradients at sufficient orientations, for any geometry of cylindrical perturbers.

To examine the combined effects of susceptibility differences and applied field gradients, we performed simulations using a geometry of a single walled cylinder with an outer radius of 1.5 µm, an inner radius of 0.7 µm in system of overall dimensions 6 x 6 x 6 μm^3 with a spatial resolution of 100 x 100 x 100 points. We were then able to perform simulations of diffusion-mediated decoherence with and without applied field gradients. Without field gradients, the anisotropy of T_2 could be determined by assigning an isotropic T_2 of 100 ms across the entire simulation region. With applied field gradients, an effective diffusion tensor could be fitted to the data as in any ordinary diffusion-weighted MRI dataset, using b-values of 0 and 1000 mm^{-2} s at a gradient amplitude of 0.04 T/m, with 6 non-collinear gradient directions. Outside the perturber region, an isotropic diffusion tensor $D_{xx}=D_{yy}=D_{zz}=0.8x10^{-3}$ mm^2 s^{-1} was assigned. In the lumen, an axially symmetric diffusion tensor with a z-axis parallel to the z-axis of the cylinder was defined with $D_{xx}=D_{yy}=0.3x10^{-3}$ mm^2 s^{-1}, $D_{zz}=1.5x10^{-3}$ mm^2 s^{-1}. In the wall, an anisotropic diffusion tensor with a z-axis parallel to the z-axis of the cylinder was defined with $D_{xx}=D_{yy}=0.6x10^{-3}$ mm^2 s^{-1}, $D_{zz}=1.3x10^{-3}$ mm^2 s^{-1}.

To examine the effects that crossing-fibre populations have on anisotropy of T_2 and diffusion parameters in the presence of field inhomogeneities, we created a perturber geometry of 32 walled cylinders. Either all 32 were parallel, or 16 were grouped and parallel in one direction, and the other 16 were parallel but grouped at an orientation 90° to the first group. The size of the system was 13 x 6.5 x 6.5 μm^3 with a spatial resolution of 200 x 100 x 100 points. The outer radius of each cylinder was 0.6 µm, the inner radius 0.35 µm. For T_2 anisotropy simulations, the time-domain contained 50 points between 0 and 1 second. For diffusion anisotropy simulations, B-values of 0 and 1000 mm^{-2} s were used, with 6 non-collinear gradient directions, and a gradient amplitude of 0.04 T/m. Diffusion parameters were as given in the single cylinder case.

Imaging parameters

In all, the MRI protocol contained the following:

3D T_1-weighted MPRAGE, sagittal, matrix size 256x256x208, resolution 0.86x0.86x0.86 mm^3, time 5:07 minutes, TI 900 ms, 2200 ms, α 9°, GRAPPA factor 2, 24 integrated reference lines.

T2 mapping was performed using a 2D multi-echo spin-echo sequence with, axial, with acquisition parameters: matrix size 162x192, 54 slices, resolution 1.15x1.15x1.98 mm^3 (including 10% slice gap), 9:50 minutes, TE 12 ms, 10 echoes acquired, TR 7000 ms, GRAPPA factor 2, 24 integrated reference lines, partial Fourier factor 7/8.

Diffusion imaging was performed using a 2D diffusion-weighted EPI pulse sequence with the parameters: axial, matrix size 128X128, 54 slices, resolution 1.88x1.88x1.98 mm^3 (including 10% slice gap), time 3:15 minutes, TE 87.4 ms, TR 2600 ms, GRAPPA factor 2, 24 integrated reference lines, partial Fourier factor 6/8, 60 bipolar diffusion-sensitising gradient directions, b-values 0 and 1000 s mm^{-2}, multi-band factor 3(5), repeated with anterior-to-posterior and posterior-to-anterior phase encoding (total time 6:30). Oblique orientations were not allowed, to simplify certain post-processing steps. Multi-band pulses used the time-shifted and phase-scrambled methods to reduce peak RF amplitude.

Fitting to experimental T2 anisotropy surfaces

In the main text, we define in Equations 13-15 a simple model for the effect of T_2 anisotropy, derived from our theory. This may be expressed in terms of the parameters $T_2^{\parallel} = 1/R_2^{iso}$ and *A*, representing the T_2 parallel to B_0 and amplitude of anisotropy respectively. We may also define the quantities $T_2^{\perp}$, representing T_2 perpendicular to B_0 and $T_2^{\Delta} = T_2^{\parallel} - T_2^{\perp}$, the "peak-to-trough" distance. In the main text we fit $T_2^{\parallel}$ and *A*. From these, the uncertainties in $T_2^{\perp}$ and T_2^{Δ} may be approximated from the relation

$$\Delta f = \sqrt{\sum_j \left(\frac{\partial f}{\partial x_j} \Delta x_j \right)^2} \qquad (41)$$

Where *f* is a function of the set of parameters x_j with uncertainties Δx_j. Then we have

$$\Delta T_2^{\Delta} = \sqrt{\frac{T_2^{\parallel 4}}{\left(1 + A T_2^{\parallel}\right)^4} \Delta A^2 + \frac{A^2 \left(2 T_2^{\parallel} + A T_2^{\parallel 2}\right)^2}{\left(1 + A T_2^{\parallel}\right)^4} \Delta A T_2^{\parallel 2}} \qquad (42)$$

$$\Delta T_2^{\perp} = \sqrt{\frac{\Delta T_2^{\parallel 2}}{\left(1 + AT_2^{\parallel}\right)^4} + \frac{T_2^{\parallel 4}}{\left(1 + AT_2^{\parallel}\right)^4} \Delta A^2} \qquad (43)$$

Regression models

The full regression model, use to determine the size of the T_2 anisotropy effect and its interaction with other terms, was

$$\begin{aligned} R_2 \approx\ & AGE + AGE^2 + \sin^4\theta + FA + FA^2 + MD + MD^2 \\ & + AGE \times \sin^4\theta + AGE \times FA + AGE \times MD \\ & + \sin^4\theta \times FA + \sin^4\theta \times MD \\ & + FA \times MD + Const \end{aligned} \qquad (44)$$

The reduced regression model, which did not include any T_2 anisotropy terms, was

$$\begin{aligned} R_2 \approx\ & AGE + AGE^2 + FA + FA^2 + MD + MD^2 \\ & + AGE \times FA + AGE \times MD \\ & + FA \times MD + Const \end{aligned} \qquad (45)$$

These two regression models were fitted to the total sets of data pooled over all 40 participants, limited to the TBSS skeleton and furthermore restricted to particular tracts or tract groups of interest.

Regression coefficients

The following tables give the regression coefficients and statistics for the models inclusive of an effect of anisotropy used to describe the R_2 data in the WM skeleton. The regression coefficients and their uncertainties are given for the z-scores due to the different scales of the data.

Table SI1 provides regression coefficients and statistics for the full regression model in the CST, SLoF, UF and entire WM skeleton, and corresponds to the models plotted in Figure 4.

Table SI1 provides regression coefficients and statistics for the full regression model in early and late-myelinating white matter and corresponds to the models plotted in Figure 5.

Table SI1: Regression output for the "full regression model" including anisotropy in the 3 selected WM tracts and the entire WM skeleton. The abbreviation sin4(theta) refers to the sine of the angle between the principle direction of diffusion and B_0 raised to 4th power, FA is fractional anisotropy of diffusion, MD is mean diffusivity of diffusion. Coeff is the label for the term in the full regression model, Beta the regression coefficient value, SE its fitted standard error.

Region	Coeff	Beta	SE	t-stat	p-value
CST	Const	0.010456	2.94E-06	3558.4	0
	FA	-0.00036257	1.64E-06	-220.64	0
	MD	-9.75E-05	1.72E-06	-56.803	0
	AGE	-0.00017853	1.54E-06	-116.14	0
	sin4(theta)	0.00025475	1.64E-06	155.43	0
	FA^2	-2.86E-05	1.49E-06	-19.211	3.34E-82
	FA:MD	4.01E-05	1.79E-06	22.449	1.61E-111
	MD^2	5.03E-05	1.33E-06	37.921	5.8283e-314
	FA:AGE	1.05E-05	1.65E-06	6.4118	1.44E-10
	MD:AGE	-1.31E-05	1.65E-06	-7.9588	1.74E-15
	AGE^2	-9.09E-05	1.76E-06	-51.602	0
	FA:sin4(theta)	3.11E-05	1.71E-06	18.14	1.68E-73
	MD:sin4(theta)	5.12E-05	1.70E-06	30.107	7.31E-199
	AGE:sin4(theta)	1.81E-05	1.60E-06	11.303	1.29E-29
SLoF	Const	0.011716	2.23E-06	5246	0
	FA	-0.00017143	1.26E-06	-135.98	0
	MD	-0.00015507	1.29E-06	-120.61	0
	AGE	-0.00028864	1.21E-06	-239.05	0
	sin4(theta)	0.00021174	1.22E-06	173.48	0
	FA^2	2.27E-05	9.70E-07	23.376	8.65E-121
	FA:MD	2.59E-05	1.33E-06	19.394	9.31E-84
	MD^2	-4.59E-05	1.00E-06	-45.887	0
	FA:AGE	-8.08E-05	1.26E-06	-64.266	0
	MD:AGE	-4.02E-05	1.26E-06	-31.991	2.52E-224
	AGE^2	-0.00013392	1.38E-06	-96.772	0
	FA:sin4(theta)	5.88E-05	1.30E-06	45.196	0
	MD:sin4(theta)	5.89E-06	1.30E-06	4.5436	5.53E-06
	AGE:sin4(theta)	-1.53E-06	1.21E-06	-1.2672	0.2051

UF	(Intercept)	0.01258	2.99E-06	4203.7	0
	FA	0.00013268	1.65E-06	80.266	0
	MD	-0.00015593	1.66E-06	-94.184	0
	AGE	-0.00030996	1.59E-06	-195.21	0
	sin4(theta)	0.00029578	1.61E-06	183.32	0
	FA^2	-6.42E-05	1.33E-06	-48.249	0
	FA:MD	5.18E-05	1.79E-06	28.833	2.11E-182
	MD^2	2.60E-06	1.42E-06	1.8318	0.066982
	FA:AGE	-3.23E-05	1.64E-06	-19.693	3.07E-86
	MD:AGE	-2.76E-05	1.68E-06	-16.464	7.32E-61
	AGE^2	-0.00014606	1.83E-06	-79.878	0
	FA:sin4(theta)	7.37E-05	1.72E-06	42.819	0
	MD:sin4(theta)	2.79E-05	1.72E-06	16.259	2.12E-59
	AGE:sin4(theta)	-4.47E-06	1.59E-06	-2.8152	0.0048754
All	Const	0.011899	9.43E-07	12625	0
	FA	-0.00022303	5.04E-07	-442.91	0
	MD	-0.00022945	5.15E-07	-445.38	0
	AGE	-0.00026989	4.86E-07	-554.82	0
	sin4(theta)	0.00035446	5.01E-07	706.95	0
	FA^2	-4.25E-05	4.35E-07	-97.815	0
	FA:MD	-3.39E-05	5.18E-07	-65.487	0
	MD^2	-3.34E-05	4.30E-07	-77.548	0
	FA:AGE	-5.45E-05	4.97E-07	-109.72	0
	MD:AGE	-2.88E-05	5.07E-07	-56.786	0
	AGE^2	-0.00012751	5.58E-07	-228.51	0
	FA:sin4(theta)	0.00018281	5.19E-07	352.05	0
	MD:sin4(theta)	2.05E-05	5.29E-07	38.815	0
	AGE:sin4(theta)	-9.87E-06	4.93E-07	-20.023	3.52E-89

Table SI2: Regression output for the full model including anisotropy in the WM skeleton classified into early-myelinating and late-myelinating voxels. The abbreviation sin4(theta) refers to the cosine of the angle between the principle direction of diffusion and B_0 raised to 4th power, FA is fractional anisotropy of diffusion, MD is mean diffusivity of diffusion. Coeff is the label for the term in the full regression model, Beta the regression coefficient value, SE its fitted standard error.

Region	Coeff	Beta	SE	t-stat	p-value
Early	Const	0.011354	2.05E-06	5540.2	0
	FA	-0.00043299	1.08E-06	-401.84	0
	MD	-0.00023383	1.12E-06	-208.86	0
	AGE	-0.00021561	1.04E-06	-208.28	0
	sin4(theta)	0.00051635	1.09E-06	473.81	0
	FA^2	1.53E-05	9.97E-07	15.325	5.32E-53
	FA:MD	-3.24E-05	1.14E-06	-28.429	1.06E-177
	MD^2	-3.21E-05	9.68E-07	-33.147	8.93E-241
	FA:AGE	-5.27E-06	1.06E-06	-4.9796	6.37E-07
	MD:AGE	-4.34E-05	1.10E-06	-39.447	0
	AGE^2	-0.00010866	1.19E-06	-91.602	0
	FA:sin4(theta)	0.00017927	1.11E-06	160.9	0

	MD:sin4(theta)	-5.01E-05	1.15E-06	-43.568	0
	AGE:sin4(theta)	-8.18E-06	1.08E-06	-7.5875	3.26E-14
Late	Const	0.012067	1.20E-06	10076	0
	FA	-9.56E-05	6.44E-07	-148.38	0
	MD	-0.00024269	6.51E-07	-372.78	0
	AGE	-0.00029601	6.28E-07	-471.08	0
	sin4(theta)	0.00023767	6.42E-07	370.41	0
	FA^2	1.77E-05	5.26E-07	33.565	6.47E-247
	FA:MD	1.15E-05	6.65E-07	17.247	1.18E-66
	MD^2	-1.97E-05	5.36E-07	-36.703	8.34E-295
	FA:AGE	-5.23E-05	6.41E-07	-81.508	0
	MD:AGE	-2.86E-05	6.50E-07	-43.987	0
	AGE^2	-0.00013155	7.22E-07	-182.29	0
	FA:sin4(theta)	8.25E-05	6.70E-07	123.06	0
	MD:sin4(theta)	4.33E-05	6.74E-07	64.238	0
	AGE:sin4(theta)	-1.20E-05	6.32E-07	-18.977	2.67E-80

Crossing fibres at different glancing angles

In the main text, we show how the T_2 and FA depend on the polar and azimuthal angles between a system of model axons and an applied magnetic field when there are mesoscopic magnetic field inhomogeneities arising due to the different magnetic susceptibility of the myelin sheath. However, the glancing angle between bundles of axons was either 0 or 90°. In Figure SI1, we include simulations at more fibre bundle glancing angles. The simulation time for Figure SI1, which includes 91 sets of polar and azimuthal angles relative to B_0 and 4 fibre glancing angles, was 14 hours on a PC with 16 GB RAM and a 4-core 3.1 GHz CPU, using Matlab 2015b.

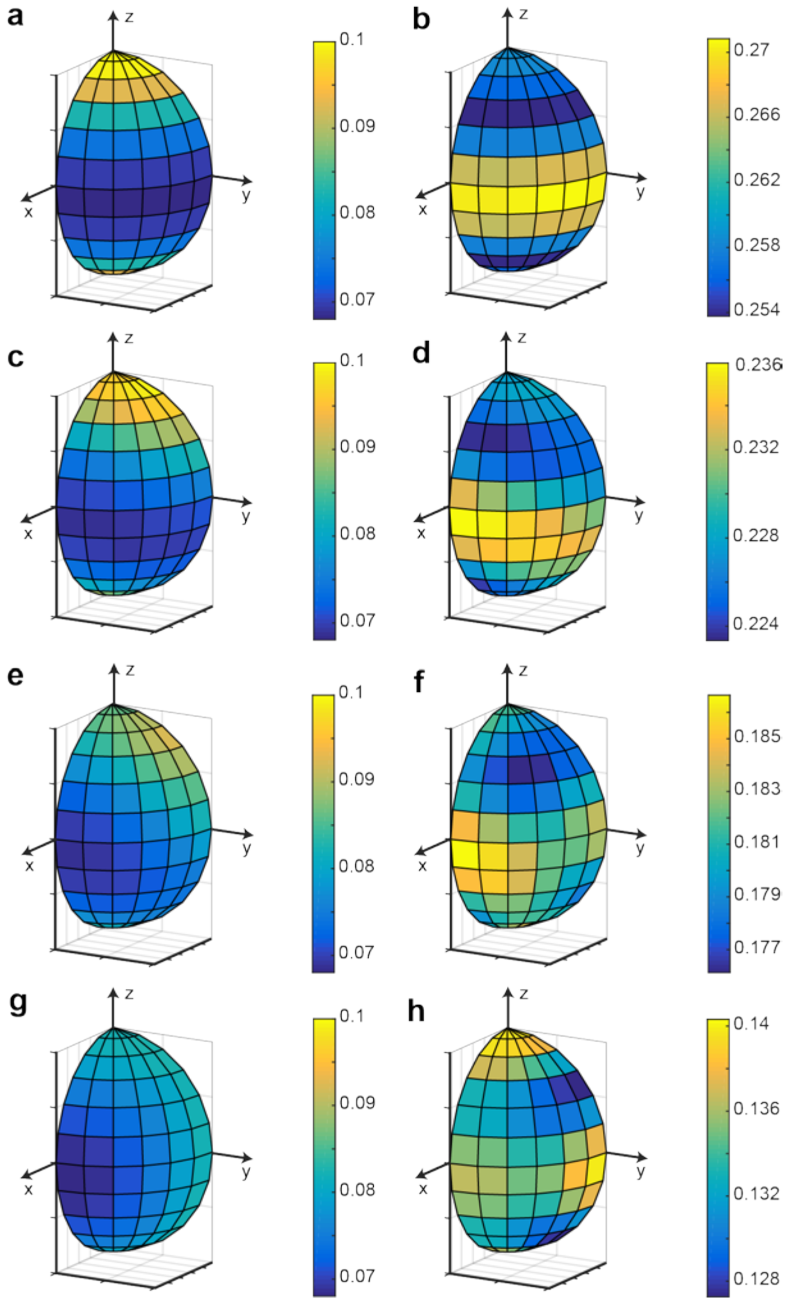

Figure SI1: T_2 and FA angular dependence relative to B_0 (parallel to z) for crossing fibre bundles with the bundles at different glancing angles. Panels a,c,e,g show the T_2, panels b,d,f,h show the FA of diffusion. The fibre glancing angles are (a,b) 0°, (c,d) 30°, (e,f) 60°, (g,h) 90°. Simulation parameters may be found in the main text. Note that the T_2 panels are all on the same colour scale, the FA panels are not due to the broader range of values.

References

1. Torrey HC (1956) Bloch Equations with Diffusion Terms. *Phys Rev* 104(3):563-565.
2. Stejskal EO & Tanner JE (1965) Spin Diffusion Measurements: Spin Echoes in the Presence of a Time-Dependent Field Gradient. *J. Chem. Phys.* 42(1):288-292.

3. Basser PJ, Mattiello J, & LeBihan D (1994) MR diffusion tensor spectroscopy and imaging. *Biophys. J.* 66(1):259-267.
4. Knight MJ & Kauppinen RA (2016) Diffusion-mediated nuclear spin phase decoherence in cylindrically porous materials. *J. Magn. Reson.* 269:1-12.
5. Feinberg DA*, et al.* (2010) Multiplexed echo planar imaging for sub-second whole brain FMRI and fast diffusion imaging. *PLoS One* 5(12):e15710.
